# Supplementary material for: Lifetime alcohol use and overall and cause-specific mortality in the European Prospective Investigation into Cancer and nutrition (EPIC) study
Source: BMJ Open. 2014 Jul 2;4(7):e005245. doi: 10.1136/bmjopen-2014-005245 (PMC4091394; doi:10.1136/bmjopen-2014-005245)
Supplement: Author's manuscript [file bmjopen-2014-005245.draft_revisions.pdf]

Lifetime alcohol use and overall and cause-specific mortality in the European Prospective Investigation into Cancer and nutrition (EPIC) study.

Journal:	*BMJ Open*
Manuscript ID:	bmjopen-2014-005245
Article Type:	Research
Date Submitted by the Author:	11-Mar-2014
Complete List of Authors:	Ferrari, Pietro; International Agency for Research on Cancer, Nutrition and Metabolism<br>Licaj, Idlir; International Agency for Research on Cancer,<br>Muller, David; International Agency for Research on Cancer,<br>Kragh-Andersen, Per; University of Copenhagen, Department of Biostatistics<br>Johansson, Mattias; International Agency for Research on Cancer,<br>Boeing, Heiner; German Institute of Human Nutrition Potsdam-Rehbruecke,<br>Weiderpass, Elisabete; University of Tromso, Department of Community Medicine, Faculty of Health Sciences<br>Dossus, Laure; INSERM, Nutrition, Hormones and Women's Health, U1018<br>Dartois, Laureen; Inserm, Center for Research in Epidemiology and Population Health<br>Fagherazzi, Guy; Inserm, U1018 Team 9<br>Bradbury, Kathryn; University of Oxford, Cancer Epidemiology Unit, Nuffield Department of Population Health<br>Wareham, Nicholas; Institute of Metabolic Science, Addenbrooke's Hospital, the Medical Research Council Epidemiology Unit<br>Duell, Eric; Catalan Institute of Oncology (ICO-IDIBELL), Unit of Nutrition, Environment and Cancer<br>Gurrea, Aurelio; Navarra Public Health Institute,<br>MOLINA-MONTES, Esther; Andalusian School of Public Health,<br>Navarro, Carmen; Murcia Regional Health Authority, Murcia,<br>Wallström, Peter; Lund University, Clinical Sciences, Malmö<br>Tjønneland, Anne; The Institute of cancer epidemiology, Danish Cancer society,<br>Olsen, Anja; Danish Cancer Society, Institute of Cancer Epidemiology<br>Trichopoulou, Antonia; Hellenic Health Foundation,,<br>Benetou, Vasiliki; University of Athens, Medical School, clinical neurophysiology<br>Trichopoulos, Dimitrios; Hellenic Health Foundation,, ; Harvard School of Public Health, Department of Epidemiology<br>Tumino, Rosario; "Civic- M.P.Arezzo" Hospital, Cancer Registry and Histopathology Unit<br>Agnoli, Claudia; Fondazione IRCCS Istituto Nazionale dei Tumori,<br>Sacerdote, Carlotta; CPO-Piemonte, ; HuGeF Foundation,<br>Palli, Domenico; CSPO-Scientific Institute of Tuscany, Molecular and Nutritional Epidemiology Unit<br>Li, Kuanrong; Division of Clinical Epidemiology, German Cancer Research Center,<br>Kaaks, Rudolf; Deutsches Krebsforschungszentrum<br>Peeters, Petra; University Medical Center Utrecht, Department of

	Epidemiology<br>Beulens, Joline; University Medical Center Utrecht, Julius Center<br>Nunes, Luciana; International Agency for Research on Cancer, Nutrition and Metabolism<br>Gunter, Marc; Imperial College, London, School of Public Health<br>Norat, Teresa; Imperial College London<br>Brennan, Paul; International Agency for Research on Cancer,<br>Overvad, Kim; Aarhus University, Department of Public Health<br>Riboli, Elio; Imperial College London, Department of Epidemiology and Biostatistics, School of Public Health<br>Romieu, Isabelle; IARC,

**Primary Subject Heading**:	Epidemiology
Secondary Subject Heading:	Public health
Keywords:	NUTRITION & DIETETICS, Epidemiology < ONCOLOGY, CARDIOLOGY

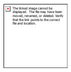

For peer review only

Lifetime alcohol use and overall and cause-specific mortality in the European Prospective Investigation into Cancer and nutrition (EPIC) study.

Pietro FERRARI[1], Idlir LICAJ[1], David C MULLER[1], Per Kragh ANDERSEN[2], Mattias JOHANSSON[1], Heiner BOEING[3], Elisabete WEIDERPASS[4,5,6,7], Laure DOSSUS[8,9,10], Laureen DARTOIS[8,9,10], Guy FAGHERAZZI[8,9,10], Kathryn E BRADBURY[11], Kay-Tee KHAW[12], Nick WAREHAM[13], Eric J DUELL[14], Aurelio BARRICARTE[15,16], Esther MOLINA-MONTES[17,18], Carmen NAVARRO SANCHEZ[18,19,20], Larraitz ARRIOLA[16,21], Peter WALLSTRÖM[22], Anne TJØNNELAND[23], Anja OLSEN[23], Antonia TRICHOPOULOU[24,25], Vasiliki BENETOU[24], Dimitrios TRICHOPOULOS[25,26,27], Rosario TUMINO[28], Claudia AGNOLI[29], Carlotta SACERDOTE[30,31], Domenico PALLI[32], Kuanrong LI[33], Rudolf KAAKS[33], Petra PEETERS[34], Joline WJ BEULENS[34], Luciana NUNES[1,35], Marc GUNTER[36], Teresa NORAT[36], Kim OVERVAD[37,38], Paul BRENNAN[1], Elio RIBOLI[36], Isabelle ROMIEU[1].

Author Affiliations:

[1]International Agency for Research on Cancer, Lyon, France.

[2]Department of Biostatistics, University of Copenhagen, Copenhagen, Denmark.

[3]German Institute of Human Nutrition, Potsdam-Rehbrücke Department of Epidemiology, Nuthetal, Germany.

[4]Department of Community Medicine, Faculty of Health Sciences, University of Tromso, The Arctic University of Norway, Tromsø, Norway.

[5]Department of Research, Cancer Registry of Norway, Oslo, Norway.

[6]Department of Medical Epidemiology and Biostatistics, Karolinska Institutet, Stockholm, Sweden.

[7]Samfundet Folkhälsan, Helsinki, Finland.

[8]Inserm, Centre for research in Epidemiology and Population Health (CESP), U1018, Nutrition, Hormones and Women's Health team, F-94805, Villejuif, France.

[9]Université Paris Sud, UMRS 1018, F-94805, Villejuif, France.

[10]IGR, F-94805, Villejuif, France.

[11]Cancer Epidemiology Unit, Nuffield Department of Population Health, University of Oxford, UK.

For peer review only

[12]the Department of Public Health and Primary Care, University of Cambridge Addenbrooke's Hospital, Cambridge, United Kingdom.

[13]the Medical Research Council Epidemiology Unit, Institute of Metabolic Science, Addenbrooke's Hospital, Cambridge, United Kingdom;

[14]Unit of Nutrition, Environment and Cancer, Cancer Epidemiology Research Program Bellvitge Biomedical Research Institute (IDIBELL), Catalan Institute of Oncology (ICO), Barcelona, Spain.

[15]Navarre Public Health Institute, Pamplona, Spain.

[16]Consortium for Biomedical Research in Epidemiology and Public Health (CIBER Epidemiología y Salud Pública-CIBERESP), Spain.

[17]Escuela Andaluza de Salud Pública. Instituto de Investigación Biosanitaria de Granada, Granada, Spain.

[18]CIBER Epidemiología y Salud Pública (CIBERESP), Spain.

[19]Department of Epidemiology, Murcia Regional Health Council, Murcia, Spain.

[20]Department of Health and Social Sciences, Universidad de Murcia, Spain.

[21]Public Health Division of Gipuzkoa, Instituto BIO-Donostia, Basque Government. Spain.

[22]Nutrition Epidemiology Research Group, Department of Clinical Sciences, Lund University, Malmö, Sweden.

[23]Danish Cancer Society Research Center, Copenhagen, Denmark.

[24]WHO Collaborating Center for Food and Nutrition Policies, Department of Hygiene, Epidemiology and Medical Statistics, University of Athens Medical School, Athens, Greece.

[25]Hellenic Health Foundation, Athens, Greece.

[26]Department of Epidemiology, Harvard School of Public Health, Boston, MA, USA.

[27]Bureau of Epidemiologic Research, Academy of Athens, Athens, Greece.

[28]Cancer Registry and Histopathology Unit, "Civic- M.P.Arezzo" Hospital, ASP Ragusa, Italy.

[29]Epidemiology and Prevention Unit, Fondazione IRCCS Istituto Nazionale dei Tumori, Milan, Via Venezian, 1, 20133 Milan, Italy.

[30]Unit of Cancer Epidemiology, AO Citta' della Salute e della Scienza-University of Turin and Center for Cancer Prevention (CPO-Piemonte), Via Santena 7, 10126 Turin, Italy.

[31]Human Genetics Foundation (HuGeF), Via Nizza 52, 10126 Turin, Italy.

[32]Molecular and Nutritional Epidemiology Unit, Cancer Research and Prevention Institute – ISPO, Florence, Italy.

For peer review only

[33]the Department of Cancer Epidemiology, German Cancer Research Centre, Heidelberg, Germany.

[34]Department of Epidemiology, Julius Center for Health Sciences and Primary Care, University Medical Center, Utrecht, The Netherlands.

[35]Department of Statistics, Federal University of Rio Grande do Sul, Porto Alegre, Brazil.

[36]Department of Epidemiology & Biostatistics, School of Public Health, Imperial College London, London, United Kingdom.

[37]Department of Public Health, Section for Epidemiology, Aarhus University, Aarhus, Denmark.

[38]Department of Cardiology, Aalborg University Hospital, Aalborg, Denmark.

Corresponding Author:

Pietro Ferrari, PhD

Nutrition and Metabolism Section

International Agency for Research on Cancer

150, cours Albert Thomas, Lyon, France

Tel +33 472728031

Email: ferrarip@iarc.fr

For peer review only

Abbreviations:

ARC: alcohol related cancers

CVD: cardiovascular diseases

CHD: coronary heart disease

HR: Hazard ratio

CI: Confidence Intervals

EPIC: European Prospective Investigation into Cancer and nutrition

Keywords: lifetime alcohol, smoking status, overall mortality.

Abstract (300 words)

Objectives. To investigate the role of factors that modulate the association between alcohol and mortality, and to provide estimates of absolute risk of death.

Design. The European Prospective Investigation into Cancer and Nutrition (EPIC).

Setting. 23 centres in 10 countries.

Participants. 380,395 men and women, free of cancer, diabetes, heart attack or stroke at enrolment, followed up for 12.6 years on average.

Main outcome measures. 20,453 fatal events, of which 2,053 alcohol related cancers (ARC, including cancers of upper aero-digestive tract, liver, colorectal and female breast), 4,187 cardiovascular diseases (CVD/CHD), 856 violent deaths and injuries. Lifetime alcohol use was assessed at recruitment.

Results. Hazard ratios comparing extreme drinkers (≥30 g/day in women and ≥60 g/day in men) to moderate drinkers (0.1-4.9 g/day) were 1.27 (95%CI: 1.13,1.43) in women and 1.53 (1.39,1.68) in men. Strong associations were observed for ARC mortality, in men particularly, and to violent deaths and injuries, in men only. No associations were observed for CVD/CHD mortality among drinkers, whereby hazard ratios were higher in never compared to moderate drinkers. Overall mortality seemed to be more strongly related to beer than wine use, particularly in men. The 10-year risks of overall death for women aged 60 years drinking more than 30 g/day was 5% and 7%, for never and current smokers, respectively. Corresponding figures in men consuming more than 60 g/day were 11% and 18%, in never and current smokers respectively. In competing risks analyses, mortality due to CVD/CHD was more pronounced than ARC in men, while CVD/CHD and ARC mortality were of similar magnitude in women.

Conclusions. In this large European cohort, alcohol use was positively associated with overall mortality, ARC and violent death and injuries, but marginally to CVD/CHD. Absolute risks of death observed in EPIC suggest that alcohol is an important determinant of total mortality.

For peer review only

Article summary

Strengths and limitations of this study

- This study was based on information on dietary and lifestyle exposure collected in a large prospective investigation of European adults.
- Findings are based on 380,395 men and women (among whom 20,453 fatal events occurred) for which iinformation on lifetime alcohol use was available, allowing separate consideration of former drinkers and lifetime abstainers.
- Exclusion of study participants reporting a morbid condition at baseline, and sensitivity analyses excluding the first three years of follow-up limited the chance that reverse causality affected the findings.
- Although statistical models included many potentially relevant adjustment factors, residual confounding might partially account for the observed associations.
- Average lifetime alcohol consumption was evaluated in this study, whereas it is possible that specific drinking patterns in particular phases of life, as well as the effect of binge drinking or drinking during meals, may also be of particular relevance for mortality.

For peer review only

Introduction

Alcohol intake has been associated with an increased risk of death from a large list of morbid conditions, including digestive tract conditions, liver cirrhosis, chronic pancreatitis, hypertension, injuries and violence (1-3). In contrast, moderate alcohol drinking was suggested to be associated with a reduction in cardiovascular disease (CVD) mortality (4;5). As for cancer, the International Agency for Research on Cancer (IARC) and the World Cancer Research Fund/American Institute for Cancer Research (WCRF/AICR) concluded that alcohol use is associated with an increased risk to develop cancers of the upper aero-digestive tract, liver, colorectal and female breast (6;7). It has been estimated that alcohol accounted for about 2.7 million annual deaths and 3.8% of all deaths worldwide (8;9), but the impact of alcohol on mortality is differential with respect to specific causes of diseases (3).

Within the European Prospective Investigation into Cancer and Nutrition (EPIC), a recent work showed that heavy alcohol use was associated to a higher risk of death from alcohol-related cancer, external causes and 'other causes', while no associations were observed for coronary heart disease and other cardiovascular diseases (10).

In this work, associations between alcohol use and overall and cause-specific mortality were further investigated. Notably, potential variability of the relationships with respect to smoking habits, the type of alcoholic beverages, and country was explored. The cumulative probabilities of death were estimated for overall mortality and, in a competing risks framework, for specific mortality causes with respect to levels of alcohol, separately in men and women. Further, the burden of alcohol use in relation to a broad group of causes of deaths was evaluated by means of overall estimates of rate advancement periods, with respect to two alternative scenarios.

For peer review only

Methods

Study Population. EPIC is an on-going multi-centre study that has been described in detail previously (11). From 1992 to 2000, 521,448 individuals, aged 25 to 70 years were recruited in the surroundings of 23 centres in 10 European countries. Most of the participants were recruited from the general population residing in a given geographic area, a town, or a province. Exceptions were the cohorts of France (female members of a health insurance for school employees), Utrecht (breast cancer screening attendees), Ragusa (blood donors and their spouses), and Oxford (mainly vegetarian and healthy eaters). Some characteristics of the study population in the different participating countries are reported in Table 1. Study participants provided informed consent and completed questionnaires on their diet, lifestyle, and medical history. The study was approved by the relevant ethical review boards of each centre and the International Agency of Research on Cancer in Lyon, France (11).

Dietary and lifestyle assessment. Diet was assessed at enrolment using validated country- or center-specific dietary questionnaires (DQ) designed to capture habitual consumption over the preceding year. Lifetime alcohol consumption was assessed based on self-reported weekly consumption of wine, beer, and liquor at ages 20, 30, 40, 50 years in the lifestyle questionnaire. Information on lifetime alcohol consumption was available for approximately 76% of EPIC participants (12). Information on smoking status and duration, physical activity during leisure time, prevalent conditions at baseline, educational attainment, anthropometric measures and reproductive history was obtained using lifestyle questionnaires.

Assessment of causes of death. Vital status and information on cause and date of death were ascertained using record linkage with cancer registries, boards of health, and death registries (Denmark, Italy, The Netherlands, Spain, the United Kingdom), or by active follow-up (France Germany, Greece). Data were coded using the 10th revision of the International

For peer review only

Statistical Classification of Diseases, Injuries and Causes of Death (ICD-10) where the underlying cause is the official cause of death.

In this work, six different causes of deaths were selected: cardiovascular disease (CVD) [I00-I99 excluding I20-I25] and coronary heart disease (CHD) [I20-I25], alcohol related cancer (ARC), including colorectal cancer [C18- C20], female breast cancer [C50], upper aero-digestive cancers (UADT, including cancer of the mouth [C01 to C10 without C08=salivary gland], larynx [C21], pharynx [C11 to C14], oesophagus [C15]), violent deaths and injuries (Injury, poisoning and certain other consequences of external causes [S00-T98]; deaths due to respiratory diseases [J00-J99]; a group for all other causes (including external causes of morbidity and mortality [V01-Y98], unknown causes [R96-R99]).

Statistical analyses. Participants from Denmark (Aarhus, Copenhagen), France, Germany (Heidelberg, Potsdam), Greece, Italy (Florence, Varese, Ragusa, Turin), the Netherlands (Utrecht), Spain (Asturias, Granada, Murcia, Navarra, San Sebastian), and the United Kingdom (Cambridge, Oxford), were eligible for this analysis. We excluded the entire cohorts of Naples (Italy), Bilthoven (The Netherlands), Sweden and Norway because no information on past alcohol use was collected (n=118,082). Further exclusions concerned participants with incomplete vital status information (n=928), who had not filled out the dietary or lifestyle questionnaires (n=11,411), and participants whose ratio of energy intake to estimated energy requirement (n=7,592), calculated in terms of gender, body weight, height, and age, was in the top or bottom 1% in order to partially reduce the impact of outlier values (13). Participants that at recruitment reported a cancer (n=13,283), diabetes (n=11,240), myocardial infarction or heart disease (n=5,266) or stroke (n=3,246) were excluded from the analyses (n= 30,665 in total).

Cox proportional hazards models were used to compute mortality hazard ratios (HR), and 95% confidence intervals, for categories of average lifetime alcohol use; never drinkers, 1–4.9

For peer review only

g/day (reference category), 5–14.9, 15–29.9, 30–59.9, ≥60 g/day. In women, the last two alcohol categories were collapsed into a ≥30 g/day group. Time in the study up to death, loss or end of follow-up, whichever came first was the primary time variable. The Breslow method was adopted for handling ties. Models were stratified by center to control for differences in questionnaire design, follow-up procedures, and other center-specific effects (13). Systematic adjustments were undertaken for age at recruitment, and other lifestyle factors Systematic adjustments were undertaken for age at recruitment, BMI and height (continuous), an indicator for participants that quitted alcohol drinking, time since alcohol quitting (continuous), smoking (never, current with 1 to 15 cigarettes/day, current with more than 15 cigarettes/day, former smoker that quitted less than 10 years before recruitment, former smoker that quitted more than 10 years before recruitment, current smoker of other than cigarettes, unknown [n=8,819]), duration of smoking (continuous), age at start smoking (less than 19 years, more than 19 years, unknown [n=39,041]), educational attainment (five categories of level of schooling: none, primary, technical, or degree or more, unknown [n=14,223]) as a proxy variable for socioeconomic status, physical activity (inactive, moderately inactive, moderately active, active, unknown [n=328]), and energy intake (continuous). In women the models were further adjusted for menopausal status (dichotomized as natural postmenopausal or surgical versus pre- or peri-menopausal, as assessed at baseline), ever use of replacement hormones, and number of full term pregnancies (nulliparous, 1 or 2 children, more than three, unknown [n=6,482]). Indicator variables specific to some of the confounding factors were used to model missing values, after checking that the parameters associated with these indicators were not statistically significantly associated with risk of death.

Models for overall and cause-specific mortality were fitted, separately for men and women. An overall test of significance of HRs related to alcohol use was determined by computing p-

For peer review only

values (p_{Wald}) for Wald test statistics compared to a chi-square distribution with degrees of freedom equal to the number of alcohol categories minus one. The proportional hazards assumption in the Cox model was satisfied and evaluated via inclusion into the disease model of interaction terms between lifetime alcohol and follow-up time. To reduce the chance of reverse causality, sensitivity analyses were run excluding the first three years of follow-up. As results were not different from those using the entire cohort, they were not shown. Analyses excluding former drinkers (4% and 5% of the study populations, in men and women respectively) provided very similar results (results not shown).

Evaluating heterogeneity. Effect modification in the relations between alcohol and mortality by smoking status (never, ever), smoking status (never and current smokers), and recruitment country was assessed. Models with main effects and interaction terms were fitted and compared to models with main effects only. The difference in log-likelihood (likelihood ratio test statistics) was compared to a chi-square distribution with degrees of freedom equal to the number of interaction terms. HRs for alcohol categories across levels of interacting variables were computed as linear combinations of main effects and interactions. Associations with wine and beer uses (each grouped as never, 0.1-2.9 g/day (reference), 3-9.9, 10-19.9, 20-39.9, ≥40 g/day, ≥20 g/day in women) and total mortality were assessed in mutually adjusted models. The difference of association for wine and beer use in relation to overall mortality was assessed by inspecting the significance of the parameter related to the arithmetic difference of wine and beer use (expressed on the log-scale plus 1 to deal with abstainers) in a model that also included their arithmetic sum. When assessing the association for wine (beer) intake, analyses were restricted to moderate lifetime drinkers of beer (wine) and spirits (below 3 g/day).

Flexible parametric survival models (14) with age as the time-scale were used to evaluate whether the association between alcohol intake and mortality rate varied by attained age. The

For peer review only

origin of the time-scale was set to 30 years as the hazard of death is essentially zero prior to that age. The baseline cumulative hazard was modeled using restricted cubic splines with three internal knots placed at evenly spaced centiles of the uncensored log-survival times in order to ensure that an equivalent number of deaths occurred between each knot (15). Interactions between alcohol intake and the time scale were modeled using restricted cubic splines with one knot placed at the median of uncensored log-survival times. HRs and differences in survival functions were calculated from the fitted models and plotted against attained age, along with confidence intervals calculated based on delta method variance estimates.

Possible departures from linearity in the association between average lifetime alcohol use and total mortality were assessed using fractional polynomials (16), a subset of generalized linear models in which various powers (−2, −1, −0.5, 0, 0.5, 1, 2, 3) of the covariate(s) of interest are entered into the linear predictor. Fractional polynomials of order two were consistently used in this work for lifetime alcohol use (17). Non-linearity was tested comparing the difference in log-likelihood of a model with the fractional polynomials with a model with a linear term only to a chi-square distribution with three degrees of freedom (16).

Absolute risks. An extension of the Cox proportional hazards model was employed to fit cause- specific associations between lifetime alcohol use and cause-specific mortality in a competing risks framework (18). An augmented dataset was created where the initial dataset is replicated a number of times equal to the different causes of death. In each replicated dataset, competing causes of deaths were set to censored observations, and the analyses were stratified by the event type (19). The relationship between each confounder variables and cause-specific mortality was assumed homogenous across causes of death. In this way competing risks was accounted for, and cumulative cause-specific and overall mortality curves were estimated for heavy (greater than 30 and 60 g/day, in women and men

For peer review only

respectively) and moderate (0.1 to 4.9 g/day) drinkers, separately for never and current smokers (20). Cumulative mortality curves were obtained for participants aged 60 years, using mean values for continuous confounding factors, and average frequencies for categorical confounders.

Quantifying the alcohol burden. The burden of alcohol on mortality was quantified with estimates of the rate advancement period (RAP) (21), according to two scenarios with threshold levels equal to 5 and 15 g/day. For overall and cause-specific risk of death, RAP were computed, dividing the log(HR) estimate comparing alcohol users above and the threshold with alcohol drinkers between 0.1 g/day and the threshold, by the log of the parameter associated with age. Never alcohol users did not enter into the estimation. Associated 95% confidence intervals were also determined. RAP estimates express the impact of a given exposure on the risk of death, by determining the time (in years) by which the risk of death is anticipated for exposed study participants compared to non-exposed.

Statistical tests were two-sided, and p-values <0.05 were considered statistically significant. All analyses were performed using SAS 9.2 (22) and Stata 12.1 (23).

For peer review only

Results

Baseline characteristics. The current analysis was based on 247,795 female and 101,935 male study participants. The median age at enrolment was 52 years for women and 53 years for men. Study participants were foll owed on average 12.6 years, accumulating 4,800,585 person-years, during which a total of 20,453 fatal events were recorded (Table 1).

Drinking patterns differed substantially between men and women (Table 2). In women, 10% (n=25,146) of participants were lifetime never drinkers, while 45% (n=112,281) and 2% (n=6,042) were moderate (0.1 to 4.9 g/day) and heavy users (>30g/day), respectively. Conversely, only 1.5% (n=1,600) of men reported having never consumed alcohol, 14%

(n=14,287) were moderate drinkers, whilst 29% (n=29,124) were heavy or extreme drinkers (30 to 59.9 and ≥60 g/day). Furthermore, the vast majority of women who were regular drinkers (total alcohol intake ≥10g/day) drank predominantly wine (91%), rather than beer (9%), whilst regular drinkers in men drank beer (46%) and wine (54%) in similar proportion.

Compared to never and moderate drinkers, women with higher alcohol use had higher levels of education and physical activity, and were more likely to be current smokers, or pre-/peri-menopausal. Never alcohol users were less likely to have used hormonal replacement therapy than alcohol drinkers. In men, the trends were somewhat less apparent. Heavy and extreme alcohol users (≥30 g/day) were more often current smokers, attained lower educational level, and had higher energy intake levels, compared to moderate drinkers. Never drinkers were physically less active than alcohol drinkers.

Lifetime alcohol and total mortality. Lifetime average alcohol use was strongly associated with total mortality, in that both never and heavy drinkers (≥30 g/day) had notably higher mortality rates than did light to moderate drinkers (0.1-4.9 g/day), a pattern that was consistently apparent among female and male study participants (Figure 1). The HR comparing never and heavy drinkers to moderate drinkers in women was 1.26 (95% CI: 1.18-1.35) and 1.27 (1.13-1.43), respectively. The corresponding HRs among men were 1.29 (1.10-1.51) for never drinkers, 1.15 (1.06-1.24) for heavy drinkers and 1.53 (1.39-1.68) for extreme drinkers (≥60 g/day).

Lifetime alcohol and cause specific mortality. In men, extreme alcohol use was associated with mortality due to alcohol related cancers ($HR_{\geq 60 \text{ vs ref}}$ = 2.62: 1.90, 3.62), other cancers (1.34: 1.13, 1.59), violent deaths and injuries (1.93: 1.27, 2.91), and other causes (1.98: 1.67, 2.34). With the exception of the category for never drinkers, alcohol intake was not associated with CVD or CHD mortality, both in women and men. Among women, heavy drinkers displayed $HR_{\geq 30 \text{ vs ref}}$ equal to 1.49 (1.07, 2.06) for alcohol related cancers. Respiratory

For peer review only

diseases were not associated with lifetime alcohol in women, while results were suggestive of an increased risk in extreme alcohol users compared to moderate users in men (Supplementary Figure 1). Dose–response relationships evaluated with fractional polynomials are displayed in Supplementary Figures 2 and 3, for women and men respectively.

Evaluating heterogeneity. In both sexes, alcohol related HRs for overall mortality were of similar magnitude in never and current smokers (Table 3). Analyses conducted by smoking intensity (never vs. heavy smokers, i.e. more than 15 cigarettes/day) produced very similar evidence (results not shown). Cause-specific analyses showed mostly homogeneous alcohol-related HRs by smoking status (results not shown). In women, beer use was more strongly related than wine to overall mortality for amount greater than 3g/day compared to the reference category (0.1 to 2.9 g/day). Lifetime never wine and beer users displayed higher risks than moderate drinkers. The associations between lifetime alcohol and overall risk of mortality were differential across country of origin in men ($p_{heterogeneity}$=0.012) but not in women ($p_{heterogeneity}$=0.511), as reported in Supplementary Figures 4 and 5, with stronger relationships observed in Northern European countries compared to Southern European countries.

The $HR_{\geq 60 \text{ vs ref}}$ for men was more pronounced at earlier ages, and approached one as attained age approached 90 years ($p_{likelihood\text{-}ratio}$ for age-varying versus age invariant parameterization=0.003), however extreme male drinkers exhibited lower cumulative survival probability than the reference group throughout the lifespan (Supplementary Figure 6). No such age varying association was apparent for women ($p_{likelihood\text{-}ratio}$=0.80).

Absolute risks. The 10-year risk of death at the age of 60 years for heavy drinkers was 5% and 7% in women (≥30 g/day), and 11% and 18% in men (≥60 g/day), for never and current smokers, respectively (Figure 2). Corresponding figures in moderate drinkers (0.1 to 4.9 g/day) were 3% and 4% in women, and 5% and 8% in men. Based on a competing risks

For peer review only - http://bmjopen.bmj.com/site/about/guidelines.xhtml

analysis, it was estimated that, at the age of 60 years, a female lifetime heavy alcohol drinker and smoker had a 10-years risk of death of 1.0% for ARC, 1.2% for CVD/CHD, and 0.2% for violent death and injuries, as displayed in Figure 3. Corresponding figures for males (≥60 g/day) were 2.2% (ARC), 5.0% (CVD/CHD) and 1% (violent death and injuries). Risks for moderate drinkers for ARC, CVD/CHD and violent death and injuries were 0.8%, 1.2%, 0.2% and 0.9%, 4.0%, 0.3%, in women and men, respectively. Consistently lower risks were observed for never smoker individuals, with estimates equal to 0.5%, 0.7% and 0.1% in women, and 1%, 2.1% and 0.3% in men.

Rate advancement period. The impact of lifetime alcohol on overall and cause-specific mortality was estimated with RAP values (Table 4). In women, RAP for overall mortality were equal to 0.36 years (95%CI: -0.05, 0.76) and 0.83 (0.26, 1.39), for the 5 and 15 g/day scenario, respectively. In men, RAP values were equal to 0.15 (-0.48, 0.76) and 1.42 (0.96, 1.89), for 5 and 15 g/day respectively. RAP values were sizeable for mortality due to alcohol related cancer (5.03: 3.07, 7.00) and violent death and injuries (11.83: 3.92, 18.17) in the second scenario.

Discussion

In this large European prospective study, the association between alcohol use and overall and cause-specific risk of death was evaluated in eight European populations. When accounting for potential confounding factors, average lifetime alcohol use was strongly associated with overall mortality, whereas lifetime never alcohol users consistently displayed a higher risk of death compared to moderate drinkers. These results are in agreement with a recent evaluation of alcohol and cause-specific mortality in EPIC (10).

This study has several strengths. It was conducted using a large prospective cohort, where dietary and lifestyle exposure information were collected on disease-free individuals.

For peer review only

Information on lifetime alcohol use was available on 76% of the cohort, allowing separate consideration of former drinkers and lifetime abstainers. Further, exclusion of study participants reporting a morbid condition at baseline, and sensitivity analyses excluding the first three years of follow-up suggest that reverse causality is unlikely to have affected the results. One potential weakness of this study is that, although statistical models included many potentially relevant adjustment factors, residual confounding might partially account for the observed associations. In addition, average lifetime alcohol consumption was used throughout this study, whereby it is possible that specific drinking patterns in particular phases of life, as well as the effect of binge drinking or drinking during meals may be of particular relevance for mortality.

A recent Russian study found that a strong relationship between vodka and risk of death (24). While an apparent J-shaped relationship between alcohol use and mortality has been reported (25;26), the interpretation of this pattern is the subject of some controversy. It has been suggested that alcohol abstinence does not truly entail greater risk of death than moderate use, and that misclassification of alcohol quantity and lack of accuracy in reporting prevalent morbid conditions at baseline in the group of never drinkers (27). This reasoning motivated our choice of considering moderate alcohol drinkers, as the reference category throughout this work. Moreover, residual and unmeasured confounding are plausible drivers of the association (28). These suggestions are supported by our findings that never drinkers are at increased risk of death due to violence and injury. This implausible association casts considerable doubt on the veracity of the apparent increased risk of death among never drinkers.

The overall mortality hazard ratio for men with extreme versus moderate alcohol use was greater at earlier ages, and approached unity as age advances towards 90 years. This result reflects the comparatively low incidence of death through middle age. Consideration of the

For peer review only

absolute risk of death, however, suggests that moderate drinkers have substantial cumulative survival advantage over extreme drinkers throughout the adult lifespan. It has been suggested that wine drinking could be more favourably associated than other alcoholic beverages to the risk of CHD and some cancers (29-32). In this study beer use displayed more apparent risk patterns than wine intake, particularly in men. Although we believe that this finding is relevant, we call for cautious interpretations of these results, as the lifestyle profile of wine and beer drinkers is profoundly different. The associations between alcohol and mortality were heterogeneous across countries in men, but not in women. This could be due to the larger amount of alcohol consumed in men than in women, naturally increasing the variability of exposure and the statistical power to detect associations, and of the larger variability characterizing drinking habits in men, such as binge drinking, drinking during meals and other societal aspects. Although no heterogeneity was observed by smoking status, at all levels of alcohol intake the mortality among smokers was higher than the mortality among non-smokers, so the absolute increase in risk associated with alcohol intake is more extreme among smokers. This differential increase in cumulative probability of death emphasises the central role of tobacco as a risk factor for mortality, as well as the potential extra harm of increased alcohol consumption.

In the EPIC study, although associations with alcohol were mostly apparent for ARC and violent death and injuries, absolute risks were highest for CVD/CHD in men, while CVD/CHD and ARC risks were of similar magnitude in women. In general, as individuals reporting a prevalent condition at recruitment (either cancer, diabetes, heart attack or stroke) were excluded from the analysis in an effort to minimize reverse causality, our estimates of absolute risks of death are possibly underestimated. RAP values were estimated to appreciate the risk benefit of alcohol drinkers if they were to modify their exposure, according to a counterfactual scenario (21;33). Consistently in men and women, RAP values for overall

For peer review only

mortality were larger when the reference category was set to 0.1 to 15 g/day than when using a threshold of 5 g/day, thus indicating that, based on the EPIC study, the benefit for drinkers could be largest if they reduced their intake to levels below 15 g/day. These results are in line with findings of a recent work in the UK population, where the reduction of overall mortality was estimated to be optimized for alcohol reduction up to a median population level of 5 g/day (34).

In a large prospective study in Europe, lifetime alcohol intake was significantly associated with both overall and ARC specific mortality. In men, positive associations were observed for violent deaths and injuries, while CVD and CHD deaths were not associated with alcohol use among drinkers. Our findings suggest that these associations do not differ between never and current smokers, and were stronger for beer than for wine use.

For peer review only

Funding statement

This work was supported by la Direction Générale de la Santé (French Ministry of Health), with contract (GR-IARC-2003-09-12-01), by the European Commission (Directorate General for Health and Consumer Affairs) and the International Agency for Research on Cancer. The national cohorts are supported by the Danish Cancer Society (Denmark); the Ligue Contre le Cancer, the Institut Gustave Roussy, the Mutuelle Générale de l'Education Nationale, and the Institut National de la Santé et de la Recherche Médicale (France); the Deutsche Krebshilfe, the Deutsches Krebsforschungszentrum, and the Federal Ministry of Education and Research (Germany); the Hellenic Health Foundation, the Stavros Niarchos Foundation, and the Hellenic Ministry of Health and Social Solidarity (Greece); the Italian Association for Research on Cancer and the National Research Council (Italy); the Dutch Ministry of Public Health, Welfare and Sports, the Netherlands Cancer Registry, LK Research Funds, Dutch Prevention Funds, the Dutch Zorg Onderzoek Nederland, the World Cancer Research Fund, and Statistics Netherlands (Netherlands); European Research Council-2009-AdG 232997 and the Nordforsk, Nordic Centre of Excellence program on Food, Nutrition and Health (Norway); the Health Research Fund, Regional Governments of Andalucýa, Asturias, Basque Country, Murcia (project 6236) and Navarra, Instituto de Salud Carlos III, Redes de Investigacion Cooperativa (RD06/0020) (Spain); the Swedish Cancer Society, the Swedish Scientific Council, and the Regional Government of Skåne (Sweden); Cancer Research UK, the Medical Research Council, the Stroke Association, the British Heart Foundation, the Department of Health, the Food Standards Agency, and the Wellcome Trust (United Kingdom). The work undertaken by David C Muller was done during the tenure of an IARC, Australia postdoctoral fellowship, supported by the Cancer Council Australia.

Contributorship statement

Pietro FERRARI, Idlir LICAJ, Isabelle ROMIEU, Paul BRENNAN, Heiner BOEING conceptualized the study and defined the analytical strategy. Pietro FERRARI and Idlir LICAJ had full access to all the data in the study and take responsibility for the integrity of the data and the accuracy of the data analysis. Pietro FERRARI, Idlir LICAJ, David C MULLER, Per Kragh ANDERSEN, Luciana NUNES performed statistical analyses and provided preliminary interpretation of findings. Pietro FERRARI, David C MULLER, Mattias JOHANSSON, Idlir LICAJ, Paul BRENNAN contributed to draft the manuscript. Elisabete WEIDERPASS, Laure DOSSUS, Laureen DARTOIS, Guy FAGHERAZZI,

For peer review only

Kathryn Erica BRADBURY, Kay-Tee KHAW, Eric Jeffrey DUELL, Aurelio BARRICARTE, Esther MOLINA-MONTES, Carmen NAVARRO SANCHEZ, Larraitz ARRIOLA, Peter WALLSTRÖM, Anne TJØNNELAND, Anja OLSEN, Antonia TRICHOPOULOU, Vasiliki BENETOU, Dimitrios TRICHOPOULOS, Rosario TUMINO, Claudia AGNOLI, Carlotta SACERDOTE, Domenico PALLI, Kuanrong LI, Rudolf KAAKS, Petra PEETERS, Joline Wilhelma Johanna BEULENE, Marc GUNTER, Teresa NORAT, Kim OVERVAD, Paul BRENNAN, Elio RIBOLI, Isabelle ROMIEU critically revised the manuscript and provided relevant intellectual input.

Competing Interest Declaration

All authors have completed the Unified Competing Interest form at www.icmje.org/coi_disclosure.pdf (available on request from the corresponding author). Dr. Kathryn E BRADBURY reports grants from Cancer Research UK, during the conduct of the study. The rest of the authors declared no support from any organisation for the submitted work, no financial relationships with any organisations that might have an interest in the submitted work in the previous three years, no other relationships or activities that could appear to have influenced the submitted work.

Sharing statement

Statistical code is available from the corresponding author by emailing ferrarip@iarc.fr.

For peer review only

Reference List

(1) Thun MJ, Peto R, Lopez AD, Monaco JH, Henley SJ, Heath CW, Jr., et al. Alcohol consumption and mortality among middle-aged and elderly U.S. adults. N Engl J Med 1997 Dec 11;337(24):1705-14.

(2) Bagnardi V, Blangiardo M, La Vecchia C., Corrao G. A meta-analysis of alcohol drinking and cancer risk. Br J Cancer 2001 Nov 30;85(11):1700-5.

(3) Rehm J, Baliunas D, Borges GL, Graham K, Irving H, Kehoe T, et al. The relation between different dimensions of alcohol consumption and burden of disease: an overview. Addiction 2010 May;105(5):817-43.

(4) Corrao G, Bagnardi V, Zambon A, La VC. A meta-analysis of alcohol consumption and the risk of 15 diseases. Prev Med 2004 May;38(5):613-9.

(5) Ronksley PE, Brien SE, Turner BJ, Mukamal KJ, Ghali WA. Association of alcohol consumption with selected cardiovascular disease outcomes: a systematic review and meta-analysis. BMJ 2011;342:d671.

(6) IARC. Alcohol consumption and ethyl carbamate. 96 ed. IARC; 2010.

(7) WCRF/AICR. Food, nutrition, physical activity and the prevention of cancer: a global perspective. Washington DC: AICR; 2007.

(8) Rehm J, Mathers C, Popova S, Thavorncharoensap M, Teerawattananon Y, Patra J. Global burden of disease and injury and economic cost attributable to alcohol use and alcohol-use disorders. Lancet 2009 Jun 27;373(9682):2223-33.

(9) Lim SS, Vos T, Flaxman AD, Danaei G, Shibuya K, Adair-Rohani H, et al. A comparative risk assessment of burden of disease and injury attributable to 67 risk factors and risk factor clusters in 21 regions, 1990-2010: a systematic analysis for the Global Burden of Disease Study 2010. Lancet 2012 Dec 15;380(9859):2224-60.

(10) Bergmann MM, Rehm J, Klipstein-Grobusch K, Boeing H, Schutze M, Drogan D, et al. The association of pattern of lifetime alcohol use and cause of death in the European Prospective Investigation into Cancer and Nutrition (EPIC) study. Int J Epidemiol 2013 Dec;42(6):1772-90.

(11) Riboli E, Hunt KJ, Slimani N, Ferrari P, Norat T, Fahey M, et al. European Prospective Investigation into Cancer and Nutrition (EPIC): study populations and data collection. Public Health Nutr 2002 Dec;5(6B):1113-24.

(12) Ferrari P, Jenab M, Norat T, Moskal A, Slimani N, Olsen A, et al. Lifetime and baseline alcohol intake and risk of colon and rectal cancers in the European prospective investigation into cancer and nutrition (EPIC). Int J Cancer 2007 Nov 1;121(9):2065-72.

(13) Ferrari P, Day NE, Boshuizen HC, Roddam A, Hoffmann K, Thiebaut A, et al. The evaluation of the diet/disease relation in the EPIC study: considerations for the calibration and the disease models. Int J Epidemiol 2008 Apr;37(2):368-78.

For peer review only

(14) Royston P, Parmar MK. The use of restricted mean survival time to estimate the treatment effect in randomized clinical trials when the proportional hazards assumption is in doubt. Stat Med 2011 Aug 30;30(19):2409-21.

(15) Heinzl H, Kaider A. Gaining more flexibility in Cox proportional hazards regression models with cubic spline functions. Comput Methods Programs Biomed 1997 Nov;54(3):201-8.

(16) Sauerbrei W, Royston P. Building multivariable prognostic and diagnostic models: Transformation of the predictors by using fractional polynomials. Journal ofthe Royal Statistical Society, Series A 1999;62:71-94.

(17) Royston P, Ambler G, Sauerbrei W. The use of fractional polynomials to model continuous risk variables in epidemiology. Int J Epidemiol 1999 Oct;28(5):964-74.

(18) Andersen PK, Geskus RB, de WT, Putter H. Competing risks in epidemiology: possibilities and pitfalls. Int J Epidemiol 2012 Jun;41(3):861-70.

(19) Putter H, Fiocco M, Geskus RB. Tutorial in biostatistics: competing risks and multi-state models. Stat Med 2007 May 20;26(11):2389-430.

(20) Rosthoj S, Andersen PK, Abildstrom SZ. SAS macros for estimation of the cumulative incidence functions based on a Cox regression model for competing risks survival data. Comput Methods Programs Biomed 2004 Apr;74(1):69-75.

(21) Brenner H, Gefeller O, Greenland S. Risk and rate advancement periods as measures of exposure impact on the occurrence of chronic diseases. Epidemiology 1993 May;4(3):229-36.

(22) SAS Institute Inc. 2002. NC, USA, Cary.

(23) Stata Statistical Software: *Release 12*. Version StataCorp. College Station, TX: StataCorp LP.: 2011.

(24) Zaridze D, Lewington S, Boroda A, Scelo G, Karpov R, Lazarev A, et al. Alcohol and mortality in Russia: prospective observational study of 151 000 adults. Lancet 2014 Jan 30;(13):10-6736.

(25) Thun MJ, Peto R, Lopez AD, Monaco JH, Henley SJ, Heath CW, Jr., et al. Alcohol consumption and mortality among middle-aged and elderly U.S. adults. N Engl J Med 1997 Dec 11;337(24):1705-14.

(26) Lin Y, Kikuchi S, Tamakoshi A, Wakai K, Kawamura T, Iso H, et al. Alcohol consumption and mortality among middle-aged and elderly Japanese men and women. Ann Epidemiol 2005 Sep;15(8):590-7.

(27) Fillmore KM, Stockwell T, Chikritzhs T, Bostrom A, Kerr W. Moderate alcohol use and reduced mortality risk: systematic error in prospective studies and new hypotheses. Ann Epidemiol 2007 May;17(5 Suppl):S16-S23.

For peer review only

(28) Naimi TS, Brown DW, Brewer RD, Giles WH, Mensah G, Serdula MK, et al. Cardiovascular risk factors and confounders among nondrinking and moderate-drinking U.S. adults. Am J Prev Med 2005 May;28(4):369-73.

(29) Klatsky AL, Friedman GD, Armstrong MA, Kipp H. Wine, liquor, beer, and mortality. Am J Epidemiol 2003 Sep 15;158(6):585-95.

(30) Maskarinec G, Meng L, Kolonel LN. Alcohol intake, body weight, and mortality in a multiethnic prospective cohort. Epidemiology 1998 Nov;9(6):654-61.

(31) Gronbaek M, Becker U, Johansen D, Gottschau A, Schnohr P, Hein HO, et al. Type of alcohol consumed and mortality from all causes, coronary heart disease, and cancer. Ann Intern Med 2000 Sep;19;133(6):411-9.

(32) Renaud SC, Gueguen R, Siest G, Salamon R. Wine, beer, and mortality in middle-aged men from eastern France. Arch Intern Med 1999 Sep 13;159(16):1865-70.

(33) Gellert C, Schottker B, Holleczek B, Stegmaier C, Muller H, Brenner H. Using rate advancement periods for communicating the benefits of quitting smoking to older smokers. Tob Control 2013 Jul;22(4):227-30.

(34) Nichols M, Scarborough P, Allender S, Rayner M. What is the optimal level of population alcohol consumption for chronic disease prevention in England? Modelling the impact of changes in average consumption levels. BMJ Open 2012 May 30;2(3):e000957.

For peer review only

Table 1. Country and sex-specific number of participants (N), person-years (PY), cause-specific and overall number of deaths.

Country	N	PY	CHD[1]	CVD[2]	Cancers					Other cancers[5]	Violent & Injuries[6]	Resp[7]	Other causes[8]	Total
					Breast	UADT[3]	Liver	Colon-rectum	Total[4]					
Women														
France	65,127	971,127	45	202	62	8	4	27	101	678	115	73	1,619	2,833
Italy	24,956	306,244	26	87	71	6	12	53	142	293	30	12	120	710
Spain	23,616	323,027	41	50	51	6	9	47	113	243	46	9	119	621
UK	50,251	651,640	387	320	160	30	15	127	332	716	85	161	1,091	3,092
Netherlands	14,583	189,531	110	137	58	14	7	78	157	370	20	65	209	1,068
Greece	14,391	143,150	139	100	41	2	10	18	71	146	26	27	94	603
Germany	27,098	307,380	56	74	64	12	14	44	134	256	35	25	114	694
Denmark	27,773	328,375	84	143	128	23	17	110	278	648	51	116	548	1,868
All	247,795	3,220,474	888	1,113	635	101	88	504	1,328	3,335	408	488	3,848	11,489
Men														
France	-	-	-	-	-	-	-	-	-	-	-	-	-	-
Italy	13,471	168,992	53	59	-	8	10	39	57	240	35	13	131	588
Spain	14,089	189,942	136	88	-	34	14	62	110	351	81	42	151	959
UK	20,452	262,720	438	229	-	41	11	68	120	567	88	171	1,040	2,653
Netherlands	-	-	-	-	-	-	-	-	-	-	-	-	-	-
Greece	9,726	90,989	193	141	-	12	20	31	63	279	49	54	99	878
Germany	19,743	221,724	167	138	-	37	25	65	127	437	84	35	264	1,253
Denmark	24,454	282,622	273	271	-	91	31	126	248	838	111	95	794	2,633
All	101,935	1,216,988	1,260	926	-	223	111	391	725	2,712	448	410	2,479	8,964

[1]CHD: Coronary heart disease [I20-I25] deaths; [2]CVD: Cardiovascular disease [I00-I99 except I20-I25] deaths; [3]UADT: deaths from upper aero-digestive cancers (including cancer of the mouth [C01 to C10 without C08=salivary gland]), larynx [C21], pharynx [C11 to C14], oesophagus [C15]); [4]Total frequency of alcohol related cancers; [5]Other cancers: deaths from all other cancers; [6]Violent deaths and injuries, including injury, poisoning and certain other consequences of external causes [S00-T98], and external causes of morbidity and mortality [V01-Y98]; [7]Resp=respiratory diseases [J00-J99].

For peer review only

Table 2. Characteristics of the study population at recruitment, according to amount and type of alcohol intake (g/day) in the EPIC study[1].

Characteristics	Unit	Never drinkers	Lifetime drinkers					Total[3]	Wine consumers[4]	Beer consumers[4]
			0.1-4.9	5-14.9	15-29.9	30-59.9[2]	>60[2]			
**Women**										
Number of participants	n	25,146	112,281	77,147	27,179	6,042	-	247,795	85,965	8,748
Person-Years	-	330,854	1,460,315	998,547	352,220	78,538	-	3,220,474	1,124,546	110,761
Age at recruitment	Years	52 (9)	52 (10)	51 (10)	49 (11)	47 (11)	-	51 (38-63)	52 (9)	46 (12)
Lifetime alcohol intake	g/day	0 (-)	2 (2)	9 (3)	20 (4)	43 (21)	-	7 (0-17)	12 (9)	11 (9)
Educational attainment[5]	%	14	22	27	33	37	-	25	28	28
Current smokers	%	13	14	18	24	31	-	17	17	28
Body mass index	kg/m^2	27 (5)	25 (5)	25 (4)	24 (4)	24 (4)	-	25 (20-31)	24 (4)	25 (4)
Height	cm	158 (6)	161 (6)	162 (6)	163 (7)	164 (6)	-	162 (153-170)	162 (6)	163 (7)
(Moderately) Active	%	26	39	44	44	43	-	40	42	42
Ever use of HRT[6]	%	16	25	29	28	25	-	25	50	34
Post-menopausal status[7]	%	50	49	49	44	38	-	48	29	20
Energy intake	kcal/day	1,848 (537)	1,943 (537)	2,015 (536)	2,090 (552)	2,195 (602)	-	1978 (542)	2,046 (544)	1,976 (545)
**Men**										
Number of participants	n	1,600	14,287	28,875	28,049	20,788	8,336	101,935	26,137	22,136
Person-Years	-	19,114	171,739	345,899	333,784	247,612	98,841	1,216,989	317,937	259,934
Age at recruitment	Years	53 (11)	53 (11)	53 (9)	52 (9)	52 (9)	52 (9)	53 (41-64)	53 (9)	52 (10)
Lifetime alcohol intake	g/day	0 (-)	2 (2)	10 (3)	22 (4)	42 (8)	94 (45)	25 (3-45)	30 (27)	22 (25)
Educational attainment[5]	%	21	30	31	32	26	14	29	22	27
Current smokers	%	28	22	25	30	36	49	30	31	33
Body mass index	kg/m^2	27 (4)	26 (4)	26 (3)	27 (3)	27 (4)	28 (4)	27 (22-31)	27 (4)	27 (4)
Height	cm	171 (7)	174 (7)	175 (7)	175 (7)	174 (7)	172 (7)	174 (165-183)	172 (7)	175 (7)
(Moderately) Active	%	42	46	50	52	52	50	50	48	52
Energy intake	kcal/day	2,284 (675)	2,267 (650)	2,315 (618)	2,417 (622)	2,569 (646)	2,789 (716)	2,427 (656)	2,487 (652)	2,369 (651)

[1]Means ± SDs are presented for continuous variables, frequencies for categorical variables; [2]In women the last alcohol category is ≥30g/day; [3]For continuous variables (with exception of energy intake), mean (10th-90th percentile) values are reported; [4]Study subjects consuming more than 10 g/day of wine (or beer), and consuming less than 3 g/day of beer (or wine); [5]Subjects with a university degree or more; [6]HRT=hormonal replacement therapy; [7]Post-menopausal women plus women who underwent an ovariectomy.

For peer review only

For peer review only - http://bmjopen.bmj.com/site/about/guidelines.xhtml

Figure 1. Number of deaths, person-years (PY) and multivariable hazard ratios[1] (HR) with 95% confidence intervals (CI) and p-value of the Wald test for statistical significance for overall and cause-specific mortality by categories of lifetime alcohol use, in women and men.

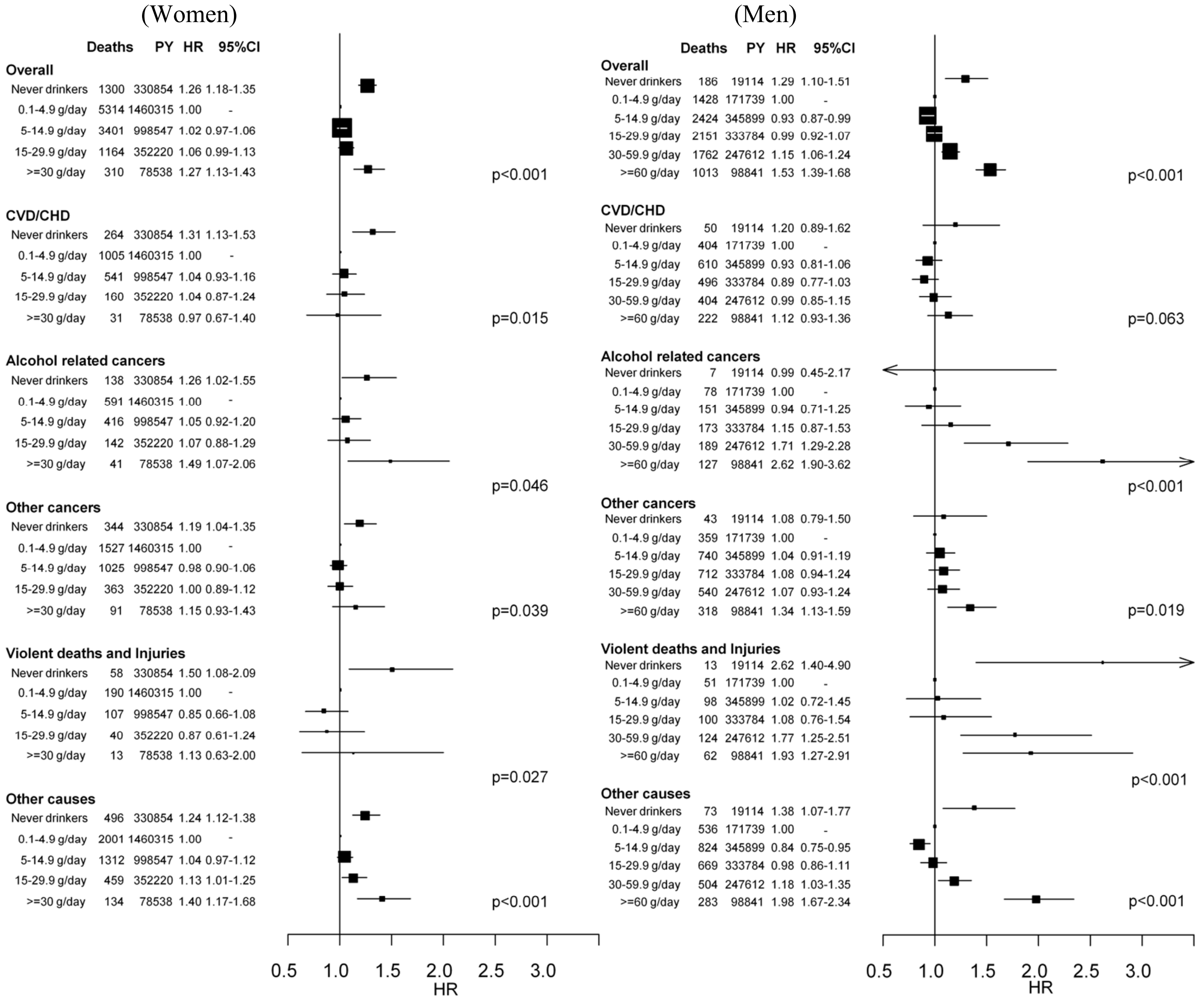

[1]Models were stratified by center. Systematic adjustment was undertaken for age at recruitment, BMI and height, former drinking, time since alcohol quitting, smoking status, duration of smoking, age at start smoking, educational attainment, and energy intake. In women adjustment was undertaken for menopausal status, ever use of replacement hormones, and number of full term pregnancies.

Table 3. Sex-specific number of deaths, hazard ratios (HR)[1] and 95%CI for overall mortality by categories of lifetime alcohol use (g/day), by smoking status (never and current smokers), and type of alcoholic beverage.

Women

	Never smokers			Current smokers			
	deaths	HR[2]	(95% CI)	deaths	HR[2]	(95% CI)	$p_{heterog}$[3]
**Overall**							
Never	1,009	1.34	(1.24, 1.45)	154	1.72	(1.32, 2.23)	
0.1-4.9	3,046	1	Ref	1,021	1.53	(1.23, 1.90)	
5-14.9	1,550	1.04	(0.98, 1.11)	874	1.51	(1.21, 1.88)	
15-29.9	397	1.04	(0.94, 1.16)	435	1.74	(1.38, 2.19)	
≥ 30	82	1.29	(1.03, 1.61)	140	2.08	(1.59, 2.73)	
p_{Wald}[4]			<0.001			<0.001	0.150
	**Wine use**			**Beer use**			
	deaths	HR[5]	(95% CI)	deaths	HR[5]	(95% CI)	$p_{difference}$[6]
**Overall**							
Never	2,156	1.15	(1.09, 1.22)	5,041	1.06	(1.02, 1.12)	
0.1-2.9	5,109	1	Ref	5,477	1	Ref	
3-9.9	2,813	0.96	(0.92, 1.01)	787	1.15	(1.07, 1.24)	
10-19.9	1,057	1.00	(0.93, 1.07)	147	1.50	(1.27, 1.77)	
≥ 20	354	1.14	(1.02, 1.27)	37	1.47	(1.06, 2.04)	
p_{Wald}[4]			<0.001			<0.001	<0.001

Men

	Never smokers			Current smokers			
	deaths	HR[2]	(95% CI)	deaths	HR[2]	(95% CI)	$p_{heterog}$[3]
**Overall**							
Never	84	1.50	(1.19, 1.21)	58	2.09	(1.26, 3.47)	
0.1-4.9	457	1	Ref	367	1.62	(1.04, 2.53)	
5-14.9	538	0.93	(0.82, 1.06)	799	1.45	(0.93, 2.25)	
15-29.9	369	1.00	(0.87, 1.16)	927	1.66	(1.06, 2.58)	
30-59.9	254	1.22	(1.23, 1.43)	857	1.83	(1.17, 2.84)	
≥ 60	107	1.56	(1.25, 1.95)	590	2.43	(1.55, 3.80)	
p_{Wald}[4]			<0.001			<0.001	0.864
	**Wine use**			**Beer use**			
	deaths	HR[5]	(95% CI)	deaths	HR[5]	(95% CI)	$p_{difference}$[6]
**Overall**							
Never	1,064	1.21	(1.12, 1.30)	975	1.07	(0.98, 1.16)	
0.1-2.9	3,266	1	Ref	2,959	1	Ref	
3-9.9	2,139	0.92	(0.87, 0.97)	2,486	1.04	(0.98, 1.10)	
10-19.9	1,040	0.96	(0.89, 1.03)	1,248	1.12	(1.04, 1.20)	
20-39.9	814	1.03	(0.95, 1.13)	877	1.41	(1.30, 1.54)	
≥ 40	641	1.22	(1.10, 1.35)	419	1.86	(1.66, 2.09)	
p_{Wald}[4]			<0.001			<0.001	<0.001

[1]Models were stratified by center. Systematic adjustment was undertaken for age at recruitment, BMI and height, former drinking, time since alcohol quitting, smoking status, duration of smoking, age at start smoking, educational attainment, and energy intake. In women adjustment was undertaken for menopausal status, ever use of replacement hormones, and number of full term pregnancies; [2]Models included interaction terms between lifetime alcohol use and a smoking indicator (0=never smokers; 1=current smokers), whereas former smokers and subjects with unknown smoking status were excluded; [3]$p_{heterogeneity}$: difference in HRs assessed comparing the log-likelihood of models with and without interaction terms between alcohol and smoking status to a 4 and 5 degrees of freedom (dof) chi-square distribution, in women and men respectively; [4]p_{Wald}: determined using a Wald test for contrasts according to a chi-square distribution with 4 and 5 dof, in women and men respectively; [5]Models on wine and beer uses were mutually adjusted, and also included spirits/liquors use; [6]$p_{difference}$ expresses the difference of associations between wine and beer use, determined evaluating the significance of the parameter estimate γ_2 in a model that included, other than the list of confounders, the terms $\gamma_1 \cdot (X_1+X_2)/2 + \gamma_2 \cdot (X_1-X_2)/2$, with X_1=log(wine use+1) and X_2=log(beer use+1).

For peer review only

Figure 2. Sex-specific plots displaying cumulative probabilities of death due to overall mortality, for heavy (≥30 g/day in women and ≥60 g/day in men, *continuous* line) and moderate lifetime use (0.1-4.9 g/day) (*dotted* line), in smokers (*black* line) and never smokers (*grey* line), for study subjects aged 60 years.

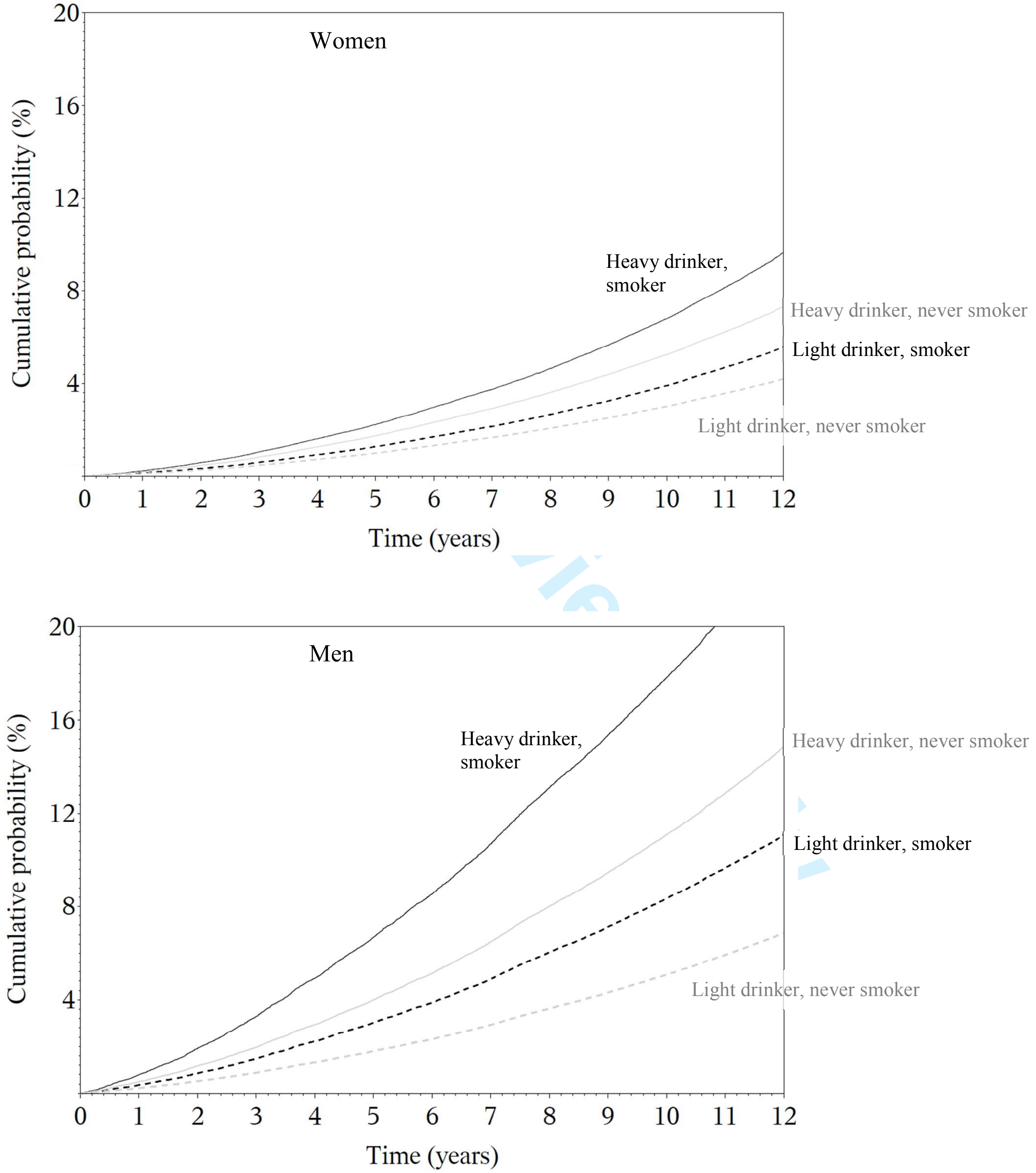

Figure 3. In competing risks analyses, sex-specific plots displaying cumulative probabilities of death due to CVD/CHD (red), alcohol related cancers (blue), and violent death and injuries (green), for study subjects aged 60 years according to heavy (≥30 g/day in women and ≥60 g/day in men, *continuous* line) and moderate (0.1-4.9 g/day, *dotted* lines) lifetime alcohol use in current and never smokers in the EPIC study.

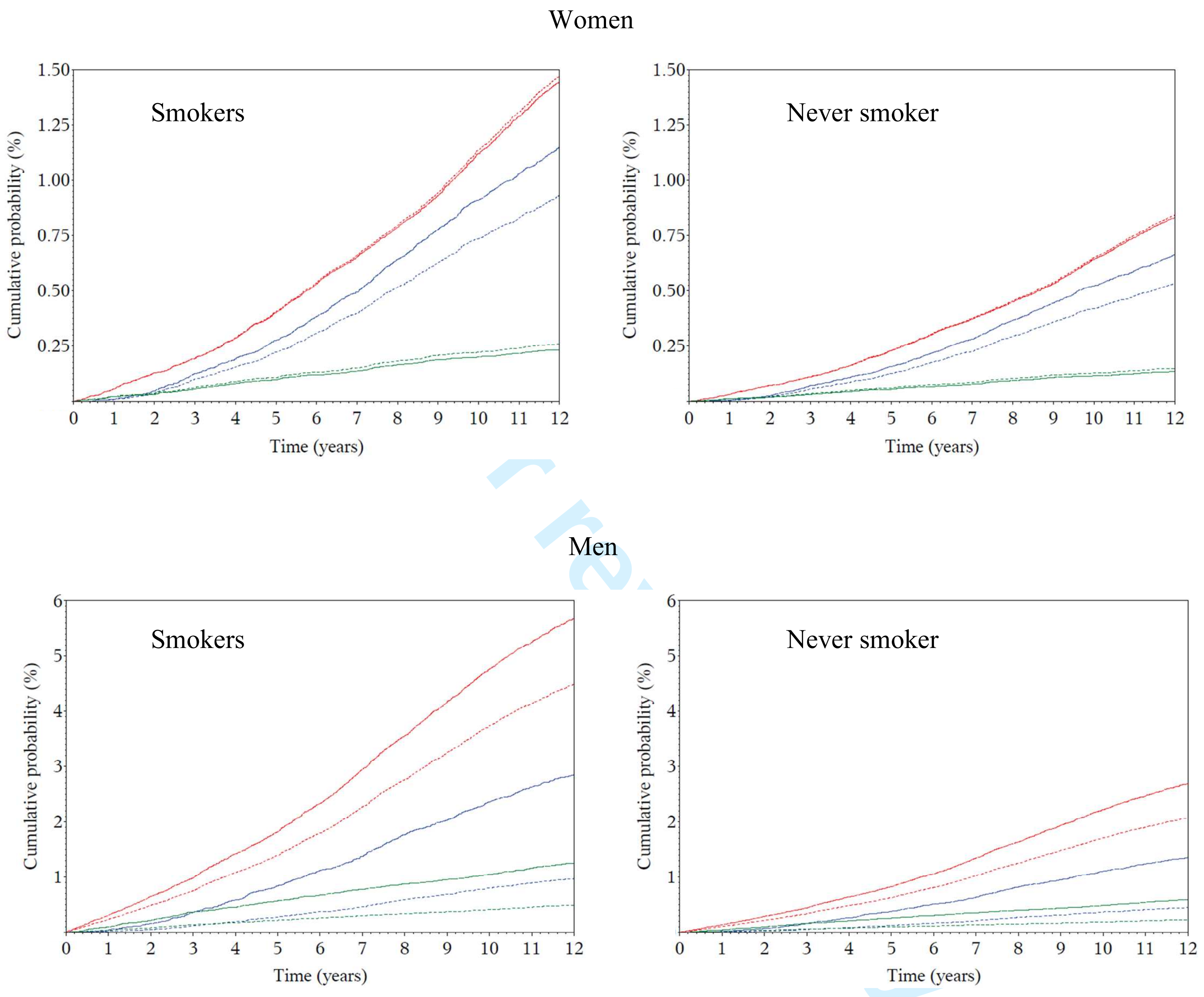

Table 4. Sex-specific estimates of rate advancement period (RAP) and associated 95%CI for overall and mortality due to ARCs, CVD/CHD and injuries and violent deaths, related to two scenarios of lifetime alcohol use. RAP estimates express the impact of a given exposure on the risk of death, by determining the time (in years) by which the risk of death is anticipated for study subjects exposed, e.g. all drinkers more than the threshold (5 or 15 g/day in Scenario I and II, respectively), compared to non-exposed, i.e. individuals drinking between 0.1 g/day and the threshold.

		Scenario I Threshold: 5 g/day		Scenario II Threshold: 15 g/day	
		RAP (years)	95%CI	RAP (years)	95%CI
Women	Overall	0.36	-0.05, 0.76	0.83	0.26, 1.39
	CVD/CHD	0.23	-0.46, 0.92	0.08	-0.96, 1.14
	Alcohol related cancers	1.28	-0.86, 3.41	1.90	-1.00, 4.81
	Injuries and violent deaths	-2.69	-6.85, 1.47	-0.20	-5.85, 5.46
Men	Overall	0.15	-0.48, 0.76	1.42	0.96, 1.89
	CVD/CHD	-0.53	-1.57, 0.50	-0.01	-0.82, 0.81
	Alcohol related cancers	2.59	-0.30, 5.49	5.03	3.07, 7.00
	Injuries and violent deaths	7.59	-2.82, 18.02	11.83	3.92, 18.17

[1]Never lifetime alcohol users did not enter into the estimation process.

For peer review only

Supplementary figure 1. Number of deaths, person-years (PY) and multivariable hazard ratios[1] (HR) with 95% confidence intervals (CI) and p-value of the Wald test for statistical significance for overall and cause-specific mortality by categories of lifetime alcohol use, in women and men.

(Women) (Men)

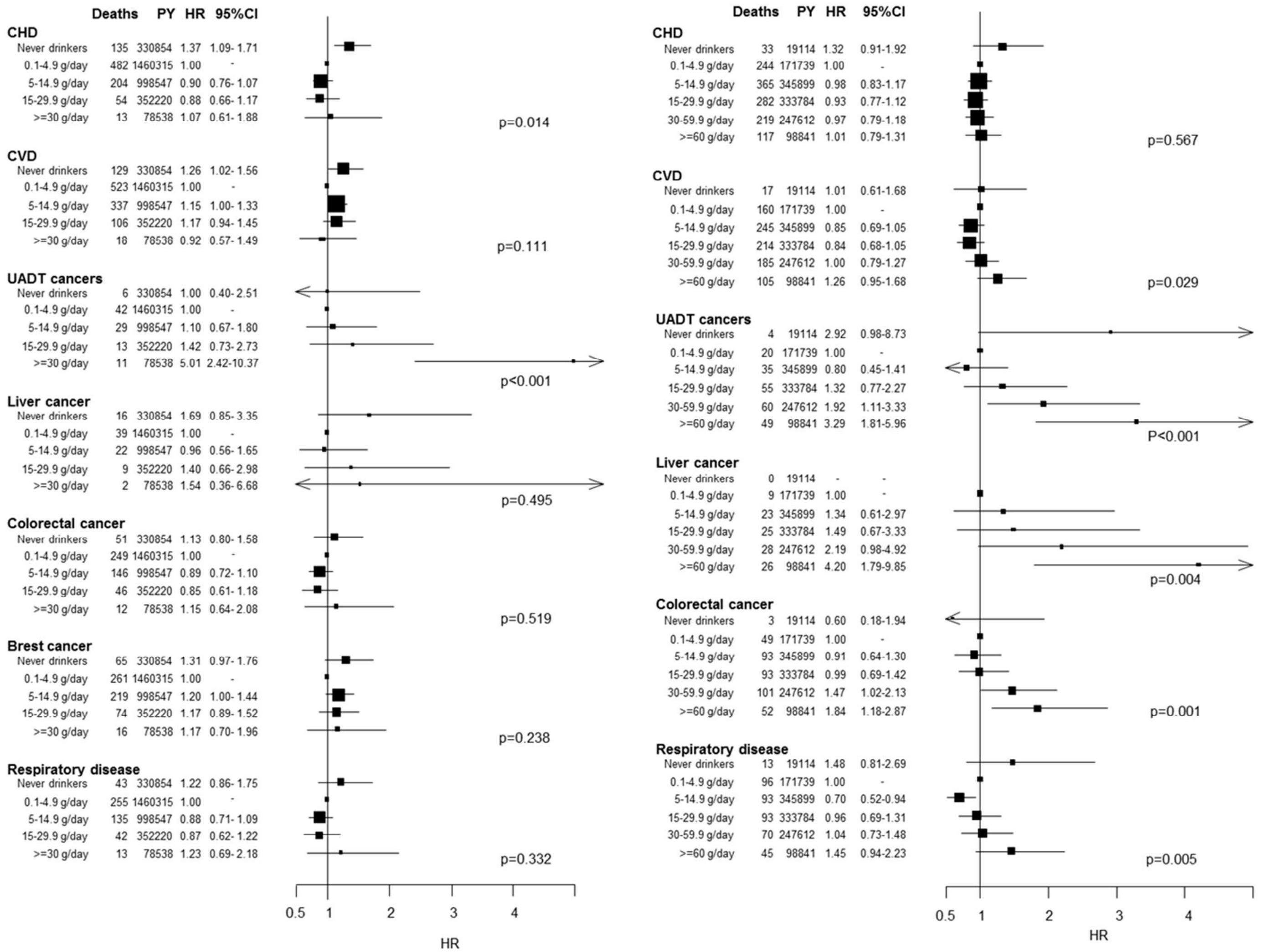

[1]Models were stratified by center. Systematic adjustment was undertaken for age at recruitment, BMI and height, former drinking, time since alcohol quitting, smoking status, duration of smoking, age at start smoking, educational attainment, and energy intake. In women adjustment was undertaken for menopausal status, ever use of replacement hormones, and number of full term pregnancies.

Supplementary figure 2. Relationship between lifetime alcohol use (g/day) and overall and cause specific risk of death (and associated 95% CI) obtained by using fractional polynomials of order two. P-linearity was obtained by comparing the log-likelihood difference of models with fractional polynomials and a linear term to a chi-square distribution with 3 degrees of freedom. **Women.**

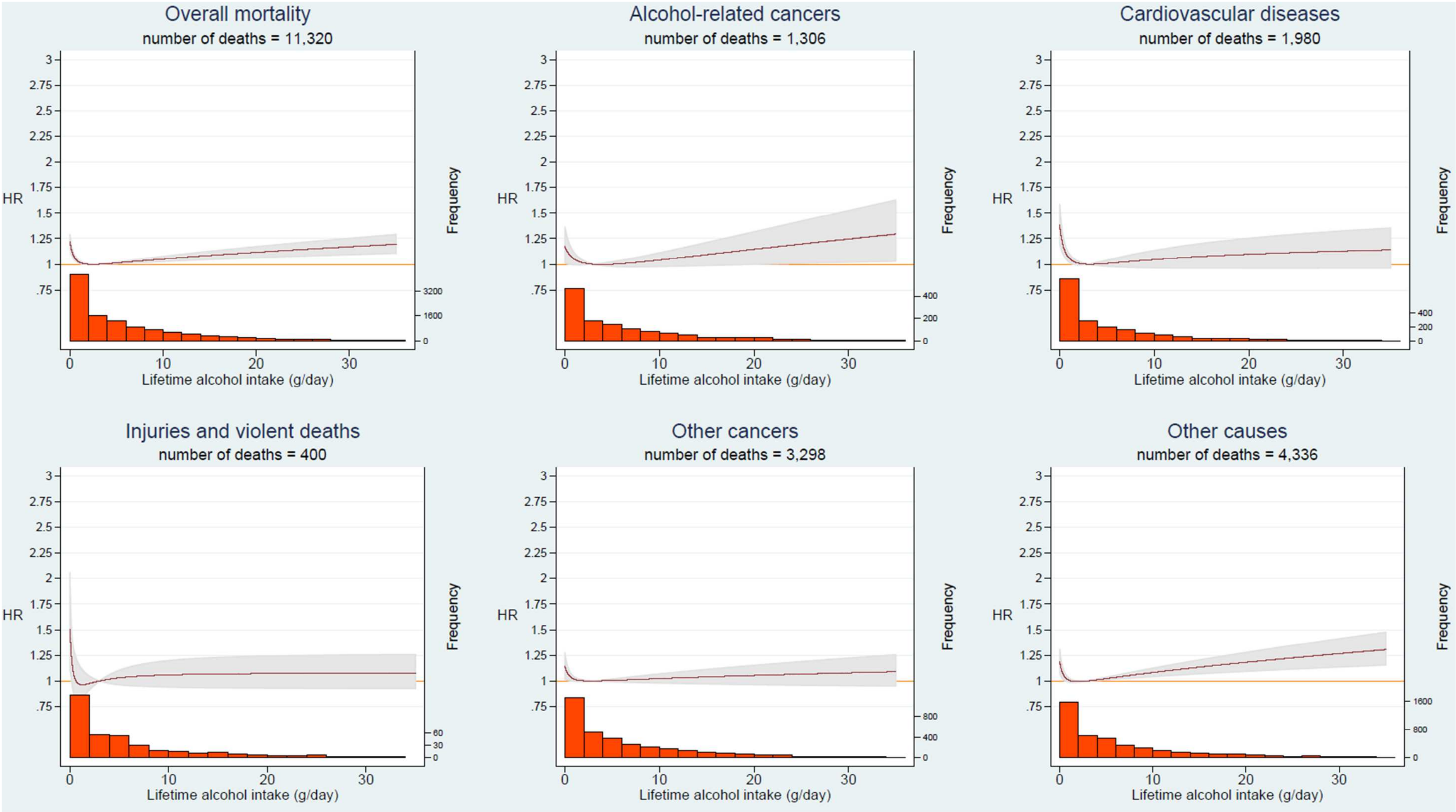

Supplementary figure 3. Relationship between lifetime alcohol use (g/day) and overall and cause specific risk of death (and associated 95% CI) obtained by using fractional polynomials of order two. P-linearity was obtained by comparing the log-likelihood difference of models with fractional polynomials and a linear term to a chi-square distribution with 3 degrees of freedom. **Men.**

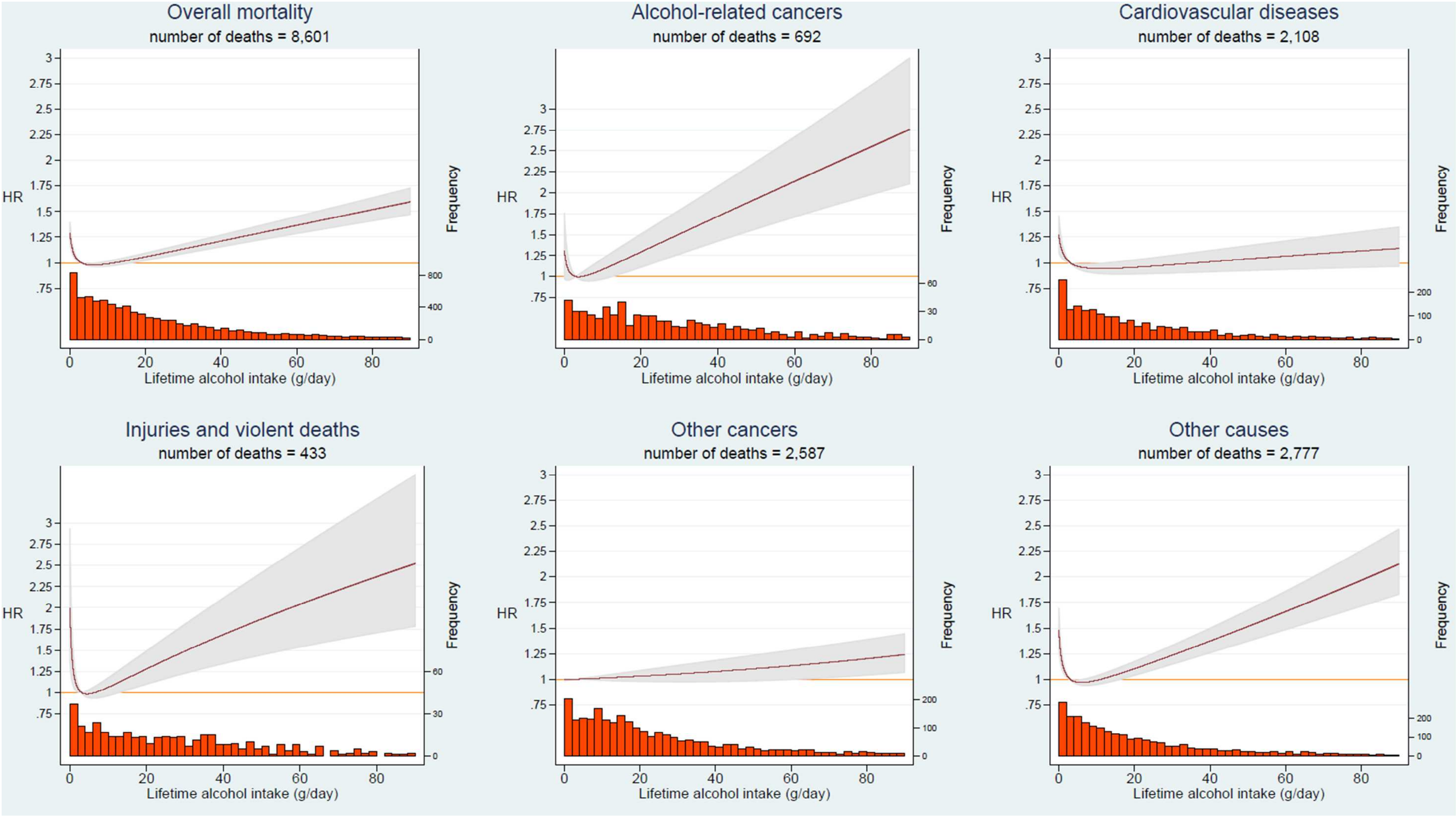

Supplementary figure 4. Associations between average alcohol use (g/day) and overall mortality (and associated 95% CI) by country of origin. $P_{heterogeneity}$ was equal to 0.511, and was obtained by comparing the log-likelihood difference of models with and without interaction terms to a chi-square statistics with 32 degrees of freedom (dof). For each country, p_{Wald} was obtained comparing the Wald test statistics to a chi-square distribution with 4 dof. Country-specific geometric means for total and subtypes of alcohol use (W=wine, B=beer, Sp=spirits/liquors in g/day) are also displayed. **Women.**

	Deaths	PY	HR	95%CI
**Denmark**				
Never drinkers	42	4274	1.46	1.07-1.99
0.1-4.9 g/day	688	121770	1.00	-
5-14.9 g/day	771	147822	1.01	0.91-1.12
15-29.9 g/day	290	46092	1.16	1.01-1.33
>=30 g/day	77	8417	1.38	0.77-2.48
**France**				
Never drinkers	322	101186	1.14	1.01-1.29
0.1-4.9 g/day	1209	460788	1.00	-
5-14.9 g/day	866	286583	1.03	0.95-1.13
15-29.9 g/day	339	98728	1.06	0.94-1.20
>=30 g/day	97	23842	1.21	0.98-1.48
**Germany**				
Never drinkers	17	4347	1.55	0.95-2.53
0.1-4.9 g/day	366	165022	1.00	-
5-14.9 g/day	231	106100	1.11	0.94-1.31
15-29.9 g/day	53	25349	1.04	0.78-1.39
>=30 g/day	27	6561	1.59	1.07-2.35
**Greece**				
Never drinkers	229	40057	1.35	1.12-1.63
0.1-4.9 g/day	224	70214	1.00	-
5-14.9 g/day	110	25380	0.98	0.78-1.23
15-29.9 g/day	35	6083	1.40	0.98-2.00
>=30 g/day	5	1416	0.87	0.36-2.10
**Italy**				
Never drinkers	97	37848	1.17	0.93-1.47
0.1-4.9 g/day	308	143624	1.00	-
5-14.9 g/day	194	84933	0.95	0.79-1.14
15-29.9 g/day	91	34375	0.96	0.76-1.22
>=30 g/day	20	5464	1.31	0.83-2.06
**Netherlands**				
Never drinkers	149	18218	1.40	1.16-1.69
0.1-4.9 g/day	455	81374	1.00	-
5-14.9 g/day	328	63099	1.04	0.90-1.20
15-29.9 g/day	111	23318	0.94	0.76-1.16
>=30 g/day	25	3522	1.22	0.81-1.83
**Spain**				
Never drinkers	240	112721	1.32	1.09-1.60
0.1-4.9 g/day	197	118191	1.00	-
5-14.9 g/day	136	64214	1.24	1.00-1.55
15-29.9 g/day	40	24426	1.04	0.74-1.46
>=30 g/day	8	3475	1.70	0.84-3.46
**UK General Population**				
Never drinkers	192	11818	1.32	1.12-1.54
0.1-4.9 g/day	918	110618	1.00	-
5-14.9 g/day	454	72087	0.98	0.87-1.10
15-29.9 g/day	96	17303	1.00	0.81-1.24
>=30 g/day	16	3501	1.11	0.68-1.83
**UK Health Conscious**				
Never drinkers	12	385	1.57	0.88-2.78
0.1-4.9 g/day	949	188713	1.00	-
5-14.9 g/day	311	148328	0.95	0.83-1.08
15-29.9 g/day	109	76545	1.05	0.86-1.28
>=30 g/day	35	22340	1.30	0.92-1.82

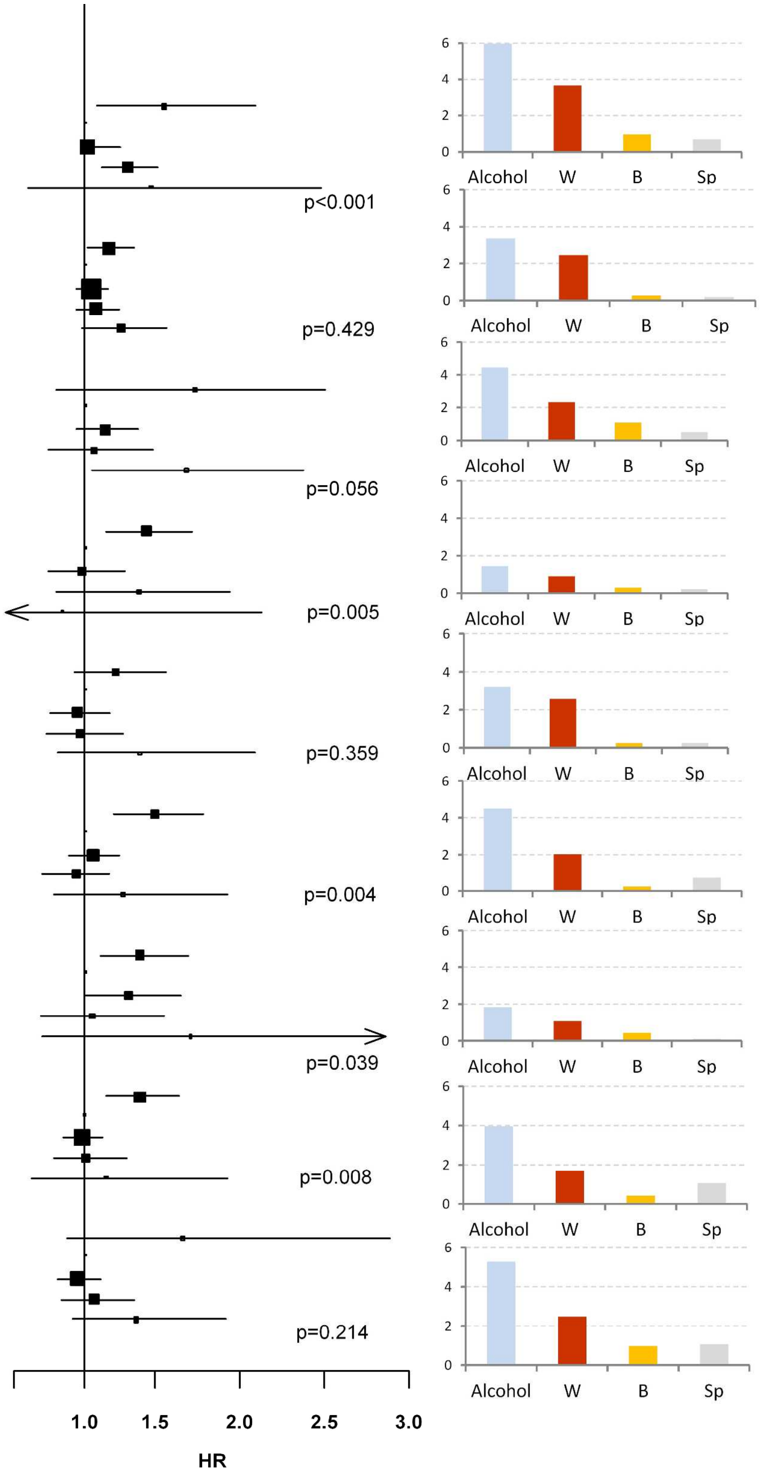

Supplementary figure 5. Associations between average lifetime alcohol use (g/day) and overall and cause specific risk of death (and associated 95% CI) by country of origin. $P_{heterogeneity}$ was equal to 0.012, and was obtained by comparing the log-likelihood difference of models with and without interaction terms to a chi-square statistics with 25 degrees of freedom (dof). For each country, p_{Wald} was obtained comparing the Wald test statistics to a chi-square distribution with 5 dof. Country-specific geometric means for total and subtypes of alcohol use (W=wine, B=beer, Sp=spirits/liquors in g/day) are also displayed. **Men.**

	Deaths	PY	HR	95%CI
**Denmark**				
Never drinkers	16	9707	1.59	0.95-2.64
0.1-4.9 g/day	201	25257	1.00	-
5-14.9 g/day	790	100810	1.01	0.87-1.18
15-29.9 g/day	843	96599	1.10	0.94-1.28
30-59.9 g/day	592	50606	1.39	1.19-1.64
>=60 g/day	191	8382	2.27	1.86-2.78
**Germany**				
Never drinkers	7	1114	1.30	0.61-2.80
0.1-4.9 g/day	106	20670	1.00	-
5-14.9 g/day	299	62507	0.89	0.71-1.11
15-29.9 g/day	329	70623	0.89	0.71-1.10
30-59.9 g/day	314	49027	1.13	0.91-1.41
>=60 g/day	198	17782	1.59	1.25-2.01
**Greece**				
Never drinkers	50	3822	1.43	1.02-2.02
0.1-4.9 g/day	95	16453	1.00	-
5-14.9 g/day	154	20391	1.04	0.80-1.34
15-29.9 g/day	186	19503	1.12	0.88-1.44
30-59.9 g/day	201	18281	1.12	0.88-1.43
>=60 g/day	192	12540	1.33	1.04-1.70
**Italy**				
Never drinkers	11	3152	0.83	0.44-1.54
0.1-4.9 g/day	102	28993	1.00	-
5-14.9 g/day	103	37583	0.73	0.55-0.96
15-29.9 g/day	147	46909	0.73	0.57-0.94
30-59.9 g/day	179	44149	0.86	0.67-1.10
>=60 g/day	46	8205	1.04	0.73-1.48
**Spain**				
Never drinkers	34	7145	1.70	1.10-2.63
0.1-4.9 g/day	51	16010	1.00	-
5-14.9 g/day	107	25612	1.31	0.94-1.83
15-29.9 g/day	158	38444	1.27	0.93-1.75
30-59.9 g/day	274	56481	1.40	1.04-1.90
>=60 g/day	335	46249	1.90	1.41-2.55
**UK General Population**				
Never drinkers	59	2798	1.15	0.87-1.51
0.1-4.9 g/day	524	30864	1.00	-
5-14.9 g/day	773	60032	0.95	0.85-1.07
15-29.9 g/day	376	29573	1.12	0.97-1.28
30-59.9 g/day	144	12434	1.04	0.86-1.26
>=60 g/day	36	2643	1.50	1.07-2.10
**UK Health Conscious**				
Never drinkers	9	113	1.51	0.78-2.93
0.1-4.9 g/day	349	33491	1.00	-
5-14.9 g/day	198	38965	0.84	0.70-1.00
15-29.9 g/day	112	32134	0.89	0.72-1.11
30-59.9 g/day	58	16634	1.11	0.84-1.48
>=60 g/day	15	3039	1.46	0.87-2.46

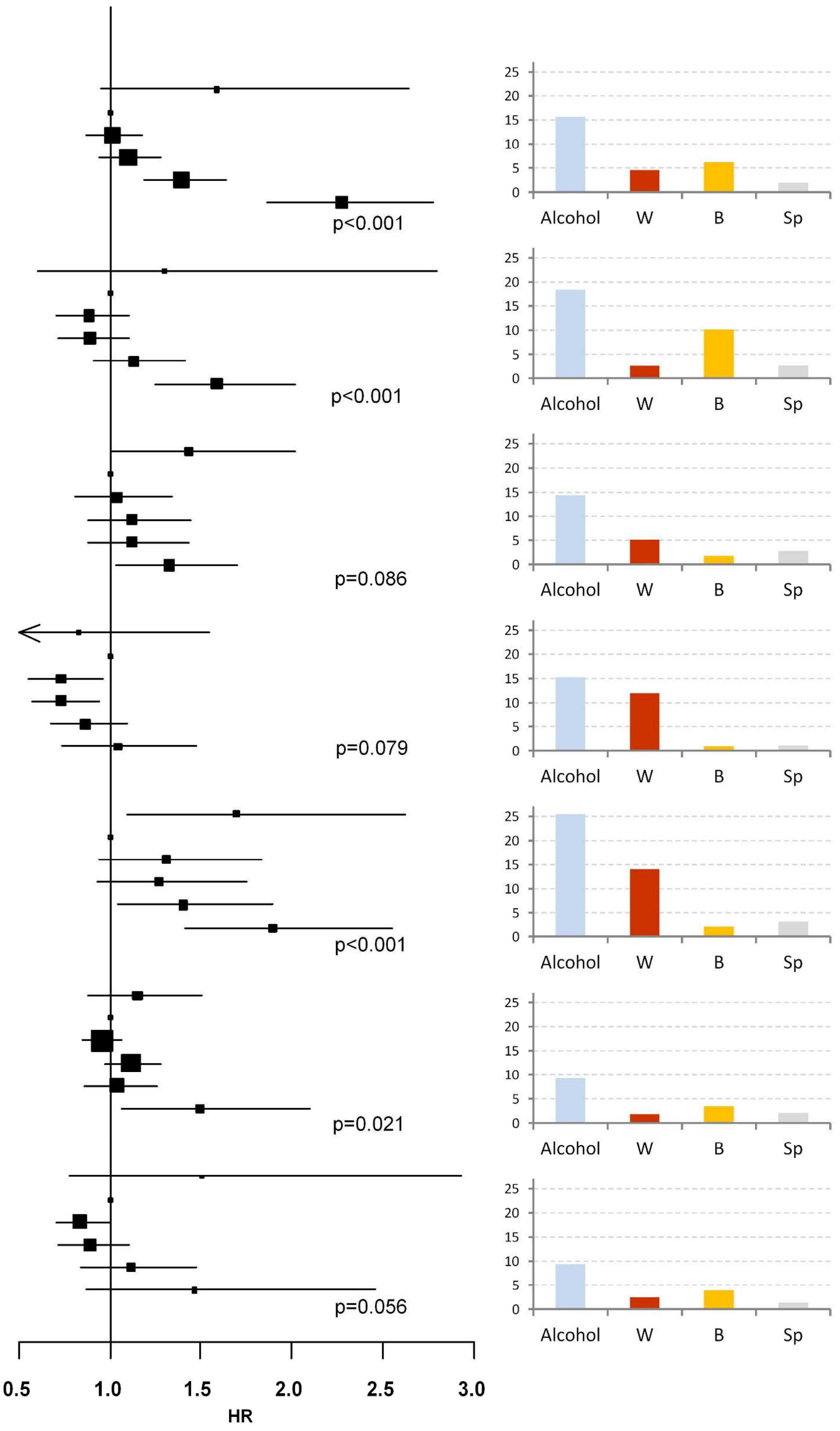

Supplementary figure 6. HRs and survival differences for heavy versus moderate drinkers as a function of attained age (40 - 90 years). Solid lines represent predictions from the model and shaded areas depict delta-method 95% CIs for the predictions. Estimates are derived from flexible parametric survival models with age as the time-scale, with the origin of the time-scale set to 30 years. Interactions between alcohol intake and the time-scale were fitted to allow for age-varying HR.

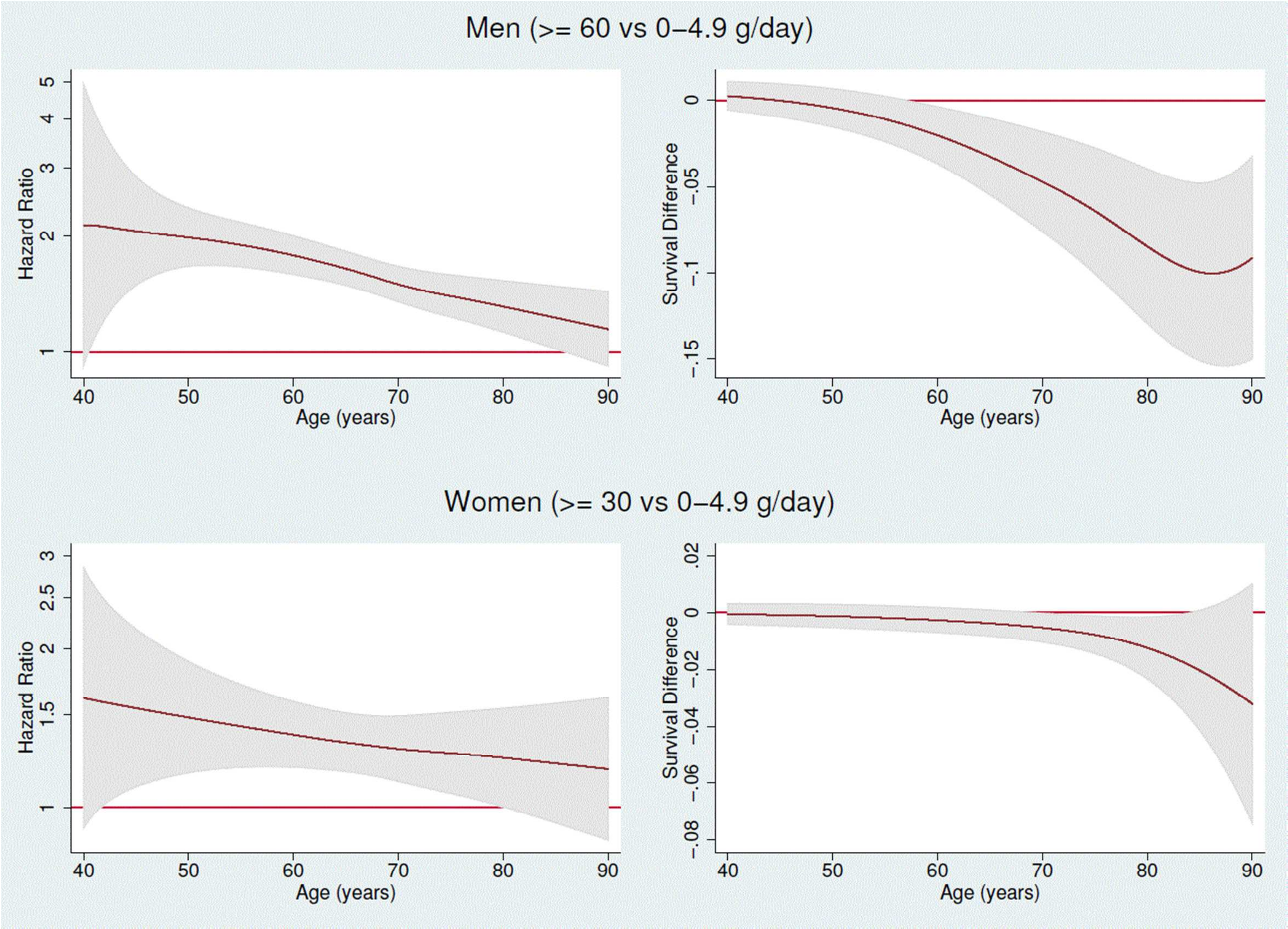

MS: "Lifetime alcohol use and overall and cause-specific mortality in the European Prospective Investigation into Cancer and nutrition (EPIC) study" by Ferrari et al.

STROBE statement checklist

(Pages of the submitted manuscript where each point of the checklist is addressed are indicated)

	Item No	Recommendation
**Title and abstract**	1	(*a*) Indicate the study's design with a commonly used term in the title or the abstract **(Pages 1, 4)**
		(*b*) Provide in the abstract an informative and balanced summary of what was done and what was found **(Page 4)**
**Introduction**		
Background/rationale	2	Explain the scientific background and rationale for the investigation being reported **(Page 6)**
Objectives	3	State specific objectives, including any prespecified hypotheses **(Page 6)**
**Methods**		
Study design	4	Present key elements of study design early in the paper **(Pages 7, 8)**
Setting	5	Describe the setting, locations, and relevant dates, including periods of recruitment, exposure, follow-up, and data collection **(Pages 7, 8)**
Participants	6	(*a*) Give the eligibility criteria, and the sources and methods of selection of participants. Describe methods of follow-up **(Pages 7, 8)**
		(*b*) For matched studies, give matching criteria and number of exposed and unexposed **(NA)**
Variables	7	Clearly define all outcomes, exposures, predictors, potential confounders, and effect modifiers. Give diagnostic criteria, if applicable **(Pages 8,9)**
Data sources/ measurement	8*	For each variable of interest, give sources of data and details of methods of assessment (measurement). Describe comparability of assessment methods if there is more than one group **(Pages 8, 9)**
Bias	9	Describe any efforts to address potential sources of bias **(Pages 10,11)**
Study size	10	Explain how the study size was arrived at **(Page 8)**
Quantitative variables	11	Explain how quantitative variables were handled in the analyses. If applicable, describe which groupings were chosen and why **(Pages 8,9)**
Statistical methods	12	(*a*) Describe all statistical methods, including those used to control for confounding **(Pages 8, 9, 10, 11)**
		(*b*) Describe any methods used to examine subgroups and interactions **(Pages 10,11)**
		(*c*) Explain how missing data were addressed **(Page 9)**
		(*d*) If applicable, explain how loss to follow-up was addressed **(Page 9)**
		(*e*) Describe any sensitivity analyses **(Page 10)**
**Results**		
Participants	13*	(a) Report numbers of individuals at each stage of study—eg numbers potentially eligible, examined for eligibility, confirmed eligible, included in the study, completing follow-up, and analysed **(Pages 7, 8, 12)**
		(b) Give reasons for non-participation at each stage **(8. For the EPIC study, details are included in Reference 11, Riboli et al, PHN, 2002)**
		(c) Consider use of a flow diagram **NO FLOW DIAGRAM**
Descriptive data	14*	(a) Give characteristics of study participants (eg demographic, clinical, social) and information on exposures and potential confounders **(Pages 12, 13, Table 2)**
		(b) Indicate number of participants with missing data for each variable of interest

For peer review only - http://bmjopen.bmj.com/site/about/guidelines.xhtml

		**(Page 9)**
		(c) Summarise follow-up time (eg, average and total amount) **(Page 12, Tables 1 and 2)**
Outcome data	15*	Report numbers of outcome events or summary measures over time **(Page 12, Table 1)**
Main results	16	(*a*) Give unadjusted estimates and, if applicable, confounder-adjusted estimates and their precision (eg, 95% confidence interval). Make clear which confounders were adjusted for and why they were included **(Page 13)**
		(*b*) Report category boundaries when continuous variables were categorized **(Pages 13, 14)**
		(*c*) If relevant, consider translating estimates of relative risk into absolute risk for a meaningful time period **(Pages 14, 15)**
Other analyses	17	Report other analyses done—eg analyses of subgroups and interactions, and sensitivity analyses **(Pages 13, 14, 15)**
**Discussion**		
Key results	18	Summarise key results with reference to study objectives **(Page 15)**
Limitations	19	Discuss limitations of the study, taking into account sources of potential bias or imprecision. Discuss both direction and magnitude of any potential bias **(Page 16)**
Interpretation	20	Give a cautious overall interpretation of results considering objectives, limitations, multiplicity of analyses, results from similar studies, and other relevant evidence **(Pages 16, 17)**
Generalisability	21	Discuss the generalisability (external validity) of the study results **(Pages 17, 18)**
**Other information**		
Funding	22	Give the source of funding and the role of the funders for the present study and, if applicable, for the original study on which the present article is based **(Pages 19, 20)**

*Give information separately for exposed and unexposed groups.

Note: An Explanation and Elaboration article discusses each checklist item and gives methodological background and published examples of transparent reporting. The STROBE checklist is best used in conjunction with this article (freely available on the Web sites of PLoS Medicine at http://www.plosmedicine.org/, Annals of Internal Medicine at http://www.annals.org/, and Epidemiology at http://www.epidem.com/). Information on the STROBE Initiative is available at http://www.strobe-statement.org.

For peer review only

Lifetime alcohol use and overall and cause-specific mortality in the European Prospective Investigation into Cancer and nutrition (EPIC) study.

Journal:	*BMJ Open*
Manuscript ID:	bmjopen-2014-005245.R1
Article Type:	Research
Date Submitted by the Author:	23-Apr-2014

Complete List of Authors:	Ferrari, Pietro; International Agency for Research on Cancer, Nutrition and Metabolism<br>Licaj, Idlir; International Agency for Research on Cancer,<br>Muller, David; International Agency for Research on Cancer,<br>Kragh-Andersen, Per; University of Copenhagen, Department of Biostatistics<br>Johansson, Mattias; International Agency for Research on Cancer,<br>Boeing, Heiner; German Institute of Human Nutrition Potsdam-Rehbruecke,<br>Weiderpass, Elisabete; University of Tromso, Department of Community Medicine, Faculty of Health Sciences<br>Dossus, Laure; INSERM, Nutrition, Hormones and Women's Health, U1018<br>Dartois, Laureen; Inserm, Center for Research in Epidemiology and Population Health<br>Fagherazzi, Guy; Inserm, U1018 Team 9<br>Bradbury, Kathryn; University of Oxford, Cancer Epidemiology Unit, Nuffield Department of Population Health<br>Khaw, Kay-Tee; University of Cambridge, Public Health and Primary Care<br>Wareham, Nicholas; Institute of Metabolic Science, Addenbrooke's Hospital, the Medical Research Council Epidemiology Unit<br>Duell, Eric; Catalan Institute of Oncology (ICO-IDIBELL), Unit of Nutrition, Environment and Cancer<br>Gurrea, Aurelio; Navarra Public Health Institute,<br>MOLINA-MONTES, Esther; Andalusian School of Public Health,<br>Navarro, Carmen; Murcia Regional Health Authority, Murcia,<br>Arriola, Larraitz; ublic Health Department of Gipuzkoa, Basque government,<br>Wallström, Peter; Lund University, Clinical Sciences, Malmö<br>Tjønneland, Anne; The Institute of cancer epidemiology, Danish Cancer society,<br>Olsen, Anja; Danish Cancer Society, Institute of Cancer Epidemiology<br>Trichopoulou, Antonia; Hellenic Health Foundation,,<br>Benetou, Vasiliki; University of Athens, Medical School, clinical neurophysiology<br>Trichopoulos, Dimitrios; Hellenic Health Foundation,, ; Harvard School of Public Health, Department of Epidemiology<br>Tumino, Rosario; "Civic- M.P.Arezzo" Hospital, Cancer Registry and Histopathology Unit<br>Agnoli, Claudia; Fondazione IRCCS Istituto Nazionale dei Tumori,<br>Sacerdote, Carlotta; CPO-Piemonte, ; HuGeF Foundation,<br>Palli, Domenico; CSPO-Scientific Institute of Tuscany, Molecular and

	Nutritional Epidemiology Unit<br>Li, Kuanrong; Division of Clinical Epidemiology, German Cancer Research Center,<br>Kaaks, Rudolf; Deutsches Krebsforschungszentrum<br>Peeters, Petra; University Medical Center Utrecht, Department of Epidemiology<br>Beulens, Joline; University Medical Center Utrecht, Julius Center<br>Nunes, Luciana; International Agency for Research on Cancer, Nutrition and Metabolism<br>Gunter, Marc; Imperial College, London, School of Public Health<br>Norat, Teresa; Imperial College London<br>Overvad, Kim; Aarhus University, Department of Public Health<br>Brennan, Paul; International Agency for Research on Cancer,<br>Riboli, Elio; Imperial College London, Department of Epidemiology and Biostatistics, School of Public Health<br>Romieu, Isabelle; IARC,

**Primary Subject Heading**:	Epidemiology
Secondary Subject Heading:	Public health
Keywords:	NUTRITION & DIETETICS, Epidemiology < ONCOLOGY, CARDIOLOGY

SCHOLARONE™ Manuscripts

Lifetime alcohol use and overall and cause-specific mortality in the European Prospective Investigation into Cancer and nutrition (EPIC) study.

Pietro FERRARI[1], Idlir LICAJ[1], David C MULLER[1], Per Kragh ANDERSEN[2], Mattias JOHANSSON[1], Heiner BOEING[3], Elisabete WEIDERPASS[4,5,6,7], Laure DOSSUS[8,9,10], Laureen DARTOIS[8,9,10], Guy FAGHERAZZI[8,9,10], Kathryn E BRADBURY[11], Kay-Tee KHAW[12], Nick WAREHAM[13], Eric J DUELL[14], Aurelio BARRICARTE[15,16], Esther MOLINA-MONTES[17,18], Carmen NAVARRO SANCHEZ[18,19,20], Larraitz ARRIOLA[16,21], Peter WALLSTRÖM[22], Anne TJØNNELAND[23], Anja OLSEN[23], Antonia TRICHOPOULOU[24,25], Vasiliki BENETOU[24], Dimitrios TRICHOPOULOS[25,26,27], Rosario TUMINO[28], Claudia AGNOLI[29], Carlotta SACERDOTE[30,31], Domenico PALLI[32], Kuanrong LI[33], Rudolf KAAKS[33], Petra PEETERS[34], Joline WJ BEULENS[34], Luciana NUNES[1,35], Marc GUNTER[36], Teresa NORAT[36], Kim OVERVAD[37,38], Paul BRENNAN[1], Elio RIBOLI[36], Isabelle ROMIEU[1].

Author Affiliations:

[1]International Agency for Research on Cancer, Lyon, France.

[2]Department of Biostatistics, University of Copenhagen, Copenhagen, Denmark.

[3]German Institute of Human Nutrition, Potsdam-Rehbrücke Department of Epidemiology, Nuthetal, Germany.

[4]Department of Community Medicine, Faculty of Health Sciences, University of Tromso, The Arctic University of Norway, Tromsø, Norway.

[5]Department of Research, Cancer Registry of Norway, Oslo, Norway.

[6]Department of Medical Epidemiology and Biostatistics, Karolinska Institutet, Stockholm, Sweden.

[7]Samfundet Folkhälsan, Helsinki, Finland.

[8]Inserm, Centre for research in Epidemiology and Population Health (CESP), U1018, Nutrition, Hormones and Women's Health team, F-94805, Villejuif, France.

[9]Université Paris Sud, UMRS 1018, F-94805, Villejuif, France.

[10]IGR, F-94805, Villejuif, France.

[11]Cancer Epidemiology Unit, Nuffield Department of Population Health, University of Oxford, UK.

For peer review only

[12]the Department of Public Health and Primary Care, University of Cambridge Addenbrooke's Hospital, Cambridge, United Kingdom.

[13]the Medical Research Council Epidemiology Unit, Institute of Metabolic Science, Addenbrooke's Hospital, Cambridge, United Kingdom;

[14]Unit of Nutrition, Environment and Cancer, Cancer Epidemiology Research Program Bellvitge Biomedical Research Institute (IDIBELL), Catalan Institute of Oncology (ICO), Barcelona, Spain.

[15]Navarre Public Health Institute, Pamplona, Spain.

[16]Consortium for Biomedical Research in Epidemiology and Public Health (CIBER Epidemiología y Salud Pública-CIBERESP), Spain.

[17]Escuela Andaluza de Salud Pública. Instituto de Investigación Biosanitaria de Granada, Granada, Spain.

[18]CIBER Epidemiología y Salud Pública (CIBERESP), Spain.

[19]Department of Epidemiology, Murcia Regional Health Council, Murcia, Spain.

[20]Department of Health and Social Sciences, Universidad de Murcia, Spain.

[21]Public Health Division of Gipuzkoa, Instituto BIO-Donostia, Basque Government. Spain.

[22]Nutrition Epidemiology Research Group, Department of Clinical Sciences, Lund University, Malmö, Sweden.

[23]Danish Cancer Society Research Center, Copenhagen, Denmark.

[24]WHO Collaborating Center for Food and Nutrition Policies, Department of Hygiene, Epidemiology and Medical Statistics, University of Athens Medical School, Athens, Greece.

[25]Hellenic Health Foundation, Athens, Greece.

[26]Department of Epidemiology, Harvard School of Public Health, Boston, MA, USA.

[27]Bureau of Epidemiologic Research, Academy of Athens, Athens, Greece.

[28]Cancer Registry and Histopathology Unit, "Civic- M.P.Arezzo" Hospital, ASP Ragusa, Italy.

[29]Epidemiology and Prevention Unit, Fondazione IRCCS Istituto Nazionale dei Tumori, Milan, Via Venezian, 1, 20133 Milan, Italy.

[30]Unit of Cancer Epidemiology, AO Citta' della Salute e della Scienza-University of Turin and Center for Cancer Prevention (CPO-Piemonte), Via Santena 7, 10126 Turin, Italy.

[31]Human Genetics Foundation (HuGeF), Via Nizza 52, 10126 Turin, Italy.

[32]Molecular and Nutritional Epidemiology Unit, Cancer Research and Prevention Institute – ISPO, Florence, Italy.

For peer review only

[33]the Department of Cancer Epidemiology, German Cancer Research Centre, Heidelberg, Germany.

[34]Department of Epidemiology, Julius Center for Health Sciences and Primary Care, University Medical Center, Utrecht, The Netherlands.

[35]Department of Statistics, Federal University of Rio Grande do Sul, Porto Alegre, Brazil.

[36]Department of Epidemiology & Biostatistics, School of Public Health, Imperial College London, London, United Kingdom.

[37]Department of Public Health, Section for Epidemiology, Aarhus University, Aarhus, Denmark.

[38]Department of Cardiology, Aalborg University Hospital, Aalborg, Denmark.

Corresponding Author:

Pietro Ferrari, PhD

Nutrition and Metabolism Section

International Agency for Research on Cancer

150, cours Albert Thomas, Lyon, France

Tel +33 472728031

Email: ferrarip@iarc.fr

Abbreviations:

ARC: alcohol related cancers

CVD: cardiovascular diseases

CHD: coronary heart disease

HR: Hazard ratio

CI: Confidence Intervals

EPIC: European Prospective Investigation into Cancer and nutrition

Keywords: lifetime alcohol, smoking status, overall mortality.

For peer review only

Abstract (300 words)

Objectives. To investigate the role of factors that modulate the association between alcohol and mortality, and to provide estimates of absolute risk of death.

Design. The European Prospective Investigation into Cancer and Nutrition (EPIC).

Setting. 23 centres in 10 countries.

Participants. 380,395 men and women, free of cancer, diabetes, heart attack or stroke at enrolment, followed up for 12.6 years on average.

Main outcome measures. 20,453 fatal events, of which 2,053 alcohol related cancers (ARC, including cancers of upper aero-digestive tract, liver, colorectal and female breast), 4,187 cardiovascular diseases (CVD/CHD), 856 violent deaths and injuries. Lifetime alcohol use was assessed at recruitment.

Results. Hazard ratios comparing extreme drinkers (≥30 g/day in women and ≥60 g/day in men) to moderate drinkers (0.1-4.9 g/day) were 1.27 (95%CI: 1.13,1.43) in women and 1.53 (1.39,1.68) in men. Strong associations were observed for ARC mortality, in men particularly, and for violent deaths and injuries, in men only. No associations were observed for CVD/CHD mortality among drinkers, whereby hazard ratios were higher in never compared to moderate drinkers. Overall mortality seemed to be more strongly related to beer than wine use, particularly in men. The 10-year risks of overall death for women aged 60 years, drinking more than 30 g/day was 5% and 7%, for never and current smokers, respectively. Corresponding figures in men consuming more than 60 g/day were 11% and 18%, in never and current smokers respectively. In competing risks analyses, mortality due to CVD/CHD was more pronounced than ARC in men, while CVD/CHD and ARC mortality were of similar magnitude in women.

Conclusions. In this large European cohort, alcohol use was positively associated with overall mortality, ARC and violent death and injuries, but marginally to CVD/CHD. Absolute risks of death observed in EPIC suggest that alcohol is an important determinant of total mortality.

For peer review only

Article summary

Strengths and limitations of this study

- This study was based on information on dietary and lifestyle exposure collected in a large prospective investigation of European adults.
- Findings are based on 380,395 men and women (among whom 20,453 fatal events occurred) for which information on lifetime alcohol use was available, allowing separate consideration of former drinkers from lifetime abstainers.
- Exclusion of study participants reporting a morbid condition at baseline, and sensitivity analyses excluding the first three years of follow-up limited the chance that reverse causality affected the findings.
- Although statistical models included many potentially relevant adjustment factors, residual confounding might partially account for the observed associations.
- Average lifetime alcohol consumption was evaluated in this study, whereas it is possible that specific drinking patterns in particular phases of life, as well as the effect of binge drinking or drinking during meals, may also be of particular relevance for mortality.

For peer review only

Introduction

Alcohol intake has been associated with an increased risk of death from a large list of morbid conditions, including digestive tract conditions, liver cirrhosis, chronic pancreatitis, hypertension, injuries and violence (1-3). In contrast, moderate alcohol drinking was suggested to be associated with a reduction in cardiovascular disease (CVD) mortality (4;5). As for cancer, the International Agency for Research on Cancer (IARC) and the World Cancer Research Fund/American Institute for Cancer Research (WCRF/AICR) concluded that alcohol use is associated with an increased risk to develop cancers of the upper aero-digestive tract, liver, colorectal and female breast (6;7). It has been estimated that alcohol accounted for about 2.7 million annual deaths and 3.8% of all deaths worldwide (8;9), but the impact of alcohol on mortality is differential with respect to specific causes of diseases (3).

Within the European Prospective Investigation into Cancer and Nutrition (EPIC), a recent work showed that heavy alcohol use was associated to a higher risk of death from alcohol-related cancer, external causes and 'other causes', while no associations were observed for coronary heart disease and other cardiovascular diseases (10).

In this work, we further investigated associations between alcohol use and overall and cause-specific mortality. Notably, potential variability of the relationships with respect to smoking habits, the type of alcoholic beverages, and country was explored. The cumulative probabilities of death were estimated for overall mortality and, in a competing risks framework, for specific mortality causes with respect to levels of alcohol, separately in men and women. Further, the burden of alcohol use in relation to a broad group of causes of deaths was evaluated by means of overall estimates of rate advancement periods, with respect to two alternative scenarios.

For peer review only

Methods

Study Population. EPIC is an on-going multi-centre study that has been described in detail previously (11). From 1992 to 2000, 521,448 individuals, aged 25 to 70 years were recruited in the surroundings of 23 centres in 10 European countries. Most of the participants were recruited from the general population residing in a given geographic area, a town, or a province. Exceptions were the cohorts of France (female members of a health insurance for school employees), Utrecht (breast cancer screening attendees), Ragusa (blood donors and their spouses), and Oxford (mainly vegetarian and healthy eaters). Some characteristics of the study population in the different participating countries are reported in Table 1. Study participants provided informed consent and completed questionnaires on their diet, lifestyle, and medical history. The study was approved by the relevant ethical review boards of each centre and the International Agency of Research on Cancer in Lyon, France (11).

Dietary and lifestyle assessment. Diet was assessed at enrolment using validated country- or center-specific dietary questionnaires (DQ) designed to capture habitual consumption over the preceding year. Lifetime alcohol consumption was assessed based on self-reported weekly consumption of wine, beer, and liquor at ages 20, 30, 40, 50 years in the lifestyle questionnaire. Information on lifetime alcohol consumption was available for approximately 76% of EPIC participants (12). Information on smoking status and duration, physical activity during leisure time, prevalent conditions at baseline, educational attainment, anthropometric measures and reproductive history was obtained using lifestyle questionnaires.

Assessment of causes of death. Vital status and information on cause and date of death were ascertained using record linkage with cancer registries, boards of health, and death registries (Denmark, Italy, The Netherlands, Spain, the United Kingdom), or by active follow-up (France Germany, Greece). Data were coded using the 10th revision of the International

For peer review only

Statistical Classification of Diseases, Injuries and Causes of Death (ICD-10) where the underlying cause is the official cause of death.

In this work, six different causes of deaths were selected: cardiovascular disease (CVD) [I00-I99 excluding I20-I25] and coronary heart disease (CHD) [I20-I25], alcohol related cancer (ARC), including colorectal cancer [C18- C20], female breast cancer [C50], upper aero-digestive cancers (UADT, including cancer of the mouth [C01 to C10 without C08=salivary gland], larynx [C21], pharynx [C11 to C14], oesophagus [C15]), violent deaths and injuries (Injury, poisoning and certain other consequences of external causes [S00-T98]; deaths due to respiratory diseases [J00-J99]; a group for all other causes (including external causes of morbidity and mortality [V01-Y98], unknown causes [R96-R99]).

Statistical analyses. Participants from Denmark (Aarhus, Copenhagen), France, Germany (Heidelberg, Potsdam), Greece, Italy (Florence, Varese, Ragusa, Turin), the Netherlands (Utrecht), Spain (Asturias, Granada, Murcia, Navarra, San Sebastian), and the United Kingdom (Cambridge, Oxford), were eligible for this analysis. We excluded the entire cohorts of Naples (Italy), Bilthoven (The Netherlands), Sweden and Norway because no information on past alcohol use was collected (n=118,082). Further exclusions concerned participants with incomplete vital status information (n=928), who had not filled out the dietary or lifestyle questionnaires (n=11,411), and participants whose ratio of energy intake to estimated energy requirement (n=7,592), calculated in terms of gender, body weight, height, and age, was in the top or bottom 1% in order to partially reduce the impact of outlier values (13). Participants that at recruitment reported a cancer (n=13,283), diabetes (n=11,240), myocardial infarction or heart disease (n=5,266) or stroke (n=3,246) were excluded from the analyses (n= 30,665 in total).

Cox proportional hazard models were used to compute mortality hazard ratios (HR), and 95% confidence intervals, for categories of average lifetime alcohol use; never drinkers, 1–4.9

For peer review only

g/day (reference category), 5–14.9, 15–29.9, 30–59.9, ≥60 g/day. In women, the last two alcohol categories were collapsed into a ≥30 g/day group. Time in the study up to death, loss or end of follow-up, whichever came first was the primary time variable. The Breslow method was adopted for handling ties. Models were stratified by center to control for differences in questionnaire design, follow-up procedures, and other center-specific effects (13). Systematic adjustments were undertaken for age at recruitment, BMI and height (continuous), an indicator for participants that quitted alcohol drinking, time since alcohol quitting (continuous), smoking (never, current with 1 to 15 cigarettes/day, current with more than 15 cigarettes/day, former smoker that quitted less than 10 years before recruitment, former smoker that quitted more than 10 years before recruitment, current smoker of other than cigarettes, unknown [n=8,819]), duration of smoking (continuous), age at start smoking (less than 19 years, more than 19 years, unknown [n=39,041]), educational attainment (five categories of level of schooling: none, primary, technical, or degree or more, unknown [n=14,223]) as a proxy variable for socioeconomic status, physical activity (inactive, moderately inactive, moderately active, active, unknown [n=328]), and energy intake (continuous). In women the models were further adjusted for menopausal status (dichotomized as natural postmenopausal or surgical versus pre- or peri-menopausal, as assessed at baseline), ever use of replacement hormones, and number of full term pregnancies (nulliparous, 1 or 2 children, more than three, unknown [n=6,482]). Indicator variables specific to some of the confounding factors were used to model missing values, after checking that the parameters associated with these indicators were not statistically significantly associated with risk of death.

Models for overall and cause-specific mortality were fitted, separately for men and women. An overall test of significance of HRs related to alcohol use was determined by computing p-values (p_{Wald}) for Wald test statistics compared to a chi-square distribution with degrees of

For peer review only

freedom equal to the number of alcohol categories minus one. The proportional hazards assumption in the Cox model was satisfied and evaluated via inclusion into the disease model of interaction terms between lifetime alcohol and follow-up time. To reduce the chance of reverse causality, sensitivity analyses were run excluding the first three years of follow-up. As results were not different from those using the entire cohort, they were not shown. Analyses excluding former drinkers (4% and 5% of the study populations, in men and women respectively) provided very similar results (results not shown).

Evaluating heterogeneity. Effect modification in the relation between alcohol and mortality by, in turn, smoking status (never, ever), smoking status (never and current smokers), and recruitment country was assessed. Models with main effects and interaction terms were fitted and compared to models with main effects only. The difference in log-likelihood (likelihood ratio test statistics) was compared to a chi-square distribution with degrees of freedom equal to the number of interaction terms. HRs for alcohol categories across levels of interacting variables were computed as linear combinations of main effects and interactions. Associations with wine and beer use (each grouped as never, 0.1-2.9 g/day (reference), 3-9.9, 10-19.9, 20-39.9, ≥40 g/day, ≥20 g/day in women) and total mortality were assessed in mutually adjusted models. The difference of association for wine and beer use in relation to overall mortality was assessed by inspecting the significance of the parameter related to the arithmetic difference of wine and beer use (expressed on the log-scale plus 1 to deal with abstainers) in a model that also included their arithmetic sum. When assessing the association for wine (beer) intake, analyses were restricted to moderate lifetime drinkers of beer (wine) and spirits (below 3 g/day).

Flexible parametric survival models (14) with age as the time-scale were used to evaluate whether the association between alcohol intake and mortality rate varied by attained age. The origin of the time-scale was set to 30 years as the hazard of death is essentially zero prior to

For peer review only

that age. The baseline cumulative hazard was modeled using restricted cubic splines with three internal knots placed at evenly spaced centiles of the uncensored log-survival times in order to ensure that an equivalent number of deaths occurred between each knot (15). Interactions between alcohol intake and the time scale were modeled using restricted cubic splines with one knot placed at the median of uncensored log-survival times. HRs and differences in survival functions were calculated from the fitted models and plotted against attained age, along with confidence intervals calculated based on delta method variance estimates.

Possible departures from linearity in the association between average lifetime alcohol use and total mortality were assessed using fractional polynomials (16), a subset of generalized linear models in which various powers (−2, −1, −0.5, 0, 0.5, 1, 2, 3) of the covariate(s) of interest are entered into the linear predictor. Fractional polynomials of order two were consistently used in this work for lifetime alcohol use (17). Non-linearity was tested comparing the difference in log-likelihood of a model with the fractional polynomials with a model with a linear term only to a chi-square distribution with three degrees of freedom (16).

Absolute risks. An extension of the Cox proportional hazards model was employed to fit cause- specific associations between lifetime alcohol use and cause-specific mortality in a competing risks framework (18). An augmented dataset was created where the initial dataset is replicated a number of times equal to the different causes of death. In each replicated dataset, competing causes of death were set to censored observations, and the analyses were stratified by the event type (19). The relationship between each confounder variables and cause-specific mortality was assumed homogenous across causes of death. In this way competing risks was accounted for, and cumulative cause-specific and overall mortality curves were estimated for heavy (greater than 30 and 60 g/day, in women and men respectively) and moderate (0.1 to 4.9 g/day) drinkers, separately for never and current

For peer review only

smokers (20). Cumulative mortality curves were obtained for participants aged 60 years, using mean values for continuous confounding factors, and average frequencies for categorical confounders.

Quantifying the alcohol burden. The burden of alcohol on mortality was quantified with estimates of the rate advancement period (RAP) (21), according to two scenarios with threshold levels equal to 5 and 15 g/day. For overall and cause-specific risk of death, RAP were computed, dividing the log(HR) estimate comparing alcohol users above and the threshold with alcohol drinkers between 0.1 g/day and the threshold, by the log of the parameter associated with age. Never alcohol users were not included in the estimation. Associated 95% confidence intervals were also determined. RAP estimates express the impact of a given exposure on the risk of death, by determining the time (in years) by which the risk of death is anticipated for exposed study participants compared to non-exposed.

Statistical tests were two-sided, and p-values <0.05 were considered statistically significant. All analyses were performed using SAS 9.2 (22) and Stata 12.1 (23).

Results

Baseline characteristics. The current analysis was based on 247,795 female and 101,935 male study participants. The median age at enrolment was 52 years for women and 53 years for men. Study participants were followed on average 12.6 years, accumulating 4,800,585 person-years, during which a total of 20,453 fatal events were recorded (Table 1).

Drinking patterns differed substantially between men and women (Table 2). In women, 10% (n=25,146) of participants were lifetime never drinkers, while 45% (n=112,281) and 2% (n=6,042) were moderate (0.1 to 4.9 g/day) and heavy users (>30g/day), respectively. Conversely, only 1.5% (n=1,600) of men reported having never consumed alcohol, 14% (n=14,287) were moderate drinkers, whilst 29% (n=29,124) were heavy or extreme drinkers

For peer review only

(30 to 59.9 and ≥60 g/day). Furthermore, the vast majority of women who were regular drinkers (total alcohol intake ≥10g/day) drank predominantly wine (91%), rather than beer (9%), whilst regular drinkers in men drank beer (46%) and wine (54%) in similar proportion.

Compared to never and moderate drinkers, women with higher alcohol use had higher levels of education and physical activity, and were more likely to be current smokers, or pre-/peri-menopausal. Never alcohol users were less likely to have used hormonal replacement therapy than alcohol drinkers. In men, the trends were somewhat less apparent. Heavy and extreme alcohol users (≥30 g/day) were more often current smokers, attained lower educational level, and had higher energy intake levels, compared to moderate drinkers. Never drinkers were physically less active than alcohol drinkers.

Lifetime alcohol and total mortality. Lifetime average alcohol use was strongly associated with total mortality, in that both never and heavy drinkers (≥30 g/day) had notably higher mortality rates than did light to moderate drinkers (0.1-4.9 g/day), a pattern that was consistently apparent among female and male study participants (Figure 1). The HR comparing never and heavy drinkers to moderate drinkers in women was 1.26 (95% CI: 1.18-1.35) and 1.27 (1.13-1.43), respectively. The corresponding HRs among men were 1.29 (1.10-1.51) for never drinkers, 1.15 (1.06-1.24) for heavy drinkers and 1.53 (1.39-1.68) for extreme drinkers (≥60 g/day).

Lifetime alcohol and cause specific mortality. In men, extreme alcohol use was associated with mortality due to alcohol related cancers ($HR_{\geq 60 \text{ vs ref}}$ = 2.62: 1.90, 3.62), other cancers (1.34: 1.13, 1.59), violent deaths and injuries (1.93: 1.27, 2.91), and other causes (1.98: 1.67, 2.34). With the exception of the category for never drinkers, alcohol intake was not associated with CVD or CHD mortality, both in women and men. Among women, heavy drinkers displayed $HR_{\geq 30 \text{ vs ref}}$ equal to 1.49 (1.07, 2.06) for alcohol related cancers. Respiratory diseases were not associated with lifetime alcohol in women, while results were suggestive of

For peer review only

an increased risk in extreme alcohol users compared to moderate users in men (Supplementary Figure 1). Dose–response relationships evaluated with fractional polynomials are displayed in Supplementary Figures 2 and 3, for women and men respectively.

Evaluating heterogeneity. In both sexes, alcohol related HRs for overall mortality were of similar magnitude in never and current smokers (Table 3). Analyses conducted by smoking intensity (never vs. heavy smokers, i.e. more than 15 cigarettes/day) produced very similar evidence (results not shown). Cause-specific analyses showed mostly homogeneous alcohol-related HRs by smoking status (results not shown). In women, beer use was more strongly related than wine to overall mortality for amounts greater than 3g/day compared to the reference category (0.1 to 2.9 g/day). Lifetime never wine and beer users displayed higher risks than moderate drinkers. The associations between lifetime alcohol and overall risk of mortality were differential across country of origin in men ($p_{heterogeneity}$=0.012) but not in women ($p_{heterogeneity}$=0.511), as reported in Supplementary Figures 4 and 5, with stronger relationships observed in Northern European countries compared to Southern European countries.

The $HR_{\geq 60 \text{ vs ref}}$ for men was more pronounced at earlier ages, and were close to one as attained age approached 90 years ($p_{likelihood\text{-}ratio}$ for age-varying versus age invariant parameterization=0.003), however extreme male drinkers exhibited lower cumulative survival probability than the reference group throughout the lifespan (Supplementary Figure 6). No such age varying association was apparent for women ($p_{likelihood\text{-}ratio}$=0.80).

Absolute risks. The 10-year risk of death at the age of 60 years for heavy drinkers was 5% and 7% in women (≥30 g/day), and 11% and 18% in men (≥60 g/day), for never and current smokers, respectively (Figure 2). Corresponding figures in moderate drinkers (0.1 to 4.9 g/day) were 3% and 4% in women, and 5% and 8% in men. Based on a competing risks analysis, it was estimated that, at the age of 60 years, a female lifetime heavy alcohol drinker

For peer review only

and smoker had a 10-year risk of death of 1.0% for ARC, 1.2% for CVD/CHD, and 0.2% for violent death and injuries, as displayed in Figure 3. Corresponding figures for males (≥60 g/day) were 2.2% (ARC), 5.0% (CVD/CHD) and 1% (violent death and injuries). Risks for moderate drinkers for ARC, CVD/CHD and violent death and injuries were 0.8%, 1.2%, 0.2% and 0.9%, 4.0%, 0.3%, in women and men, respectively. Consistently lower risks were observed for never smoker individuals, with estimates equal to 0.5%, 0.7% and 0.1% in women, and 1%, 2.1% and 0.3% in men.

Rate advancement period. The impact of lifetime alcohol on overall and cause-specific mortality was estimated with RAP values (Table 4). In women, RAP for overall mortality were equal to 0.36 years (95%CI: -0.05, 0.76) and 0.83 (0.26, 1.39), for the 5 and 15 g/day scenario, respectively. In men, RAP values were equal to 0.15 (-0.48, 0.76) and 1.42 (0.96, 1.89), for 5 and 15 g/day respectively. RAP values were sizeable for mortality due to alcohol related cancer (5.03: 3.07, 7.00) and violent death and injuries (11.83: 3.92, 18.17) in the second scenario.

Discussion

In this large European prospective study, the association between alcohol use and overall and cause-specific risk of death was evaluated in eight European populations. When accounting for potential confounding factors, average lifetime alcohol use was strongly associated with overall mortality, whereas lifetime never alcohol users consistently displayed a higher risk of death compared to moderate drinkers. These results are in agreement with a recent evaluation of alcohol and cause-specific mortality in EPIC (10). With respect to this recent study (10), further analyses were conducted to deeply investigate the role of factors that modulate the association between alcohol use and the risk of death, notably smoking and types of alcohol beverage. Estimates of 10-year risk of death in relation to levels of alcohol use were provided.

For peer review only

This study has several strengths. It was conducted using a large prospective cohort, where dietary and lifestyle exposure information were collected on disease-free individuals. Information on lifetime alcohol use was available on 76% of the cohort, allowing separate consideration of former drinkers and lifetime abstainers. Further, exclusion of study participants reporting a morbid condition at baseline, and sensitivity analyses excluding the first three years of follow-up suggest that reverse causality is unlikely to have affected the results. One potential weakness of this study is that, although statistical models included many potentially relevant adjustment factors, residual confounding might partially account for the observed associations. In addition, average lifetime alcohol consumption was used throughout this study, whereby it is possible that specific drinking patterns in particular phases of life (10), as well as the effect of binge drinking or drinking during meals may be of particular relevance for mortality.

A recent Russian study found a strong relationship between vodka and risk of death (24). While an apparent J-shaped relationship between alcohol use and mortality has been reported (25;26), the interpretation of this pattern is the subject of some controversy. It has been suggested that alcohol abstinence does not truly entail greater risk of death than moderate use, and that misclassification of alcohol quantity and lack of accuracy in reporting prevalent morbid conditions at baseline in the group of never drinkers (27) could explain the excess risks observed. This reasoning motivated our choice of considering moderate alcohol drinkers, as the reference category throughout this work. Moreover, residual and unmeasured confounding are plausible drivers of the association (28). These suggestions are supported by our findings that never drinkers are at increased risk of death due to violence and injury. This implausible association casts considerable doubt on the veracity of the apparent increased risk of death among never drinkers.

For peer review only

The overall mortality hazard ratio for men with extreme versus moderate alcohol use was greater at younger ages, and approached one as age increased towards 90 years. This result reflects the comparatively low incidence of death through middle age. Consideration of the absolute risk of death, however, suggests that moderate drinkers have a substantial cumulative survival advantage over extreme drinkers throughout the adult lifespan. It has been suggested that wine drinking could be more favourably associated than other alcoholic beverages to the risk of CHD and some cancers (29-32). In this study beer use displayed more apparent risk patterns than wine consumption, particularly in men. Although we believe that this finding is relevant, we call for cautious interpretations of these results, as the lifestyle profile of wine and beer drinkers is profoundly different. The associations between alcohol and mortality were heterogeneous across countries in men, but not in women. This could be due to the larger amount of alcohol consumed in men than in women, naturally increasing the variability of exposure and the statistical power to detect associations, and of the larger variability characterizing drinking habits in men, such as binge drinking, drinking during meals and other societal aspects. Although no heterogeneity was observed by smoking status, mortality among smokers was higher than the mortality among non-smokers , so the absolute increase in risk associated with alcohol intake is more extreme among smokers. This differential increase in cumulative probability of death emphasises the central role of tobacco as a risk factor for mortality, as well as the potential extra harm of increased alcohol consumption.

In the EPIC study, although associations with alcohol were mostly apparent for ARC and violent death and injuries, absolute risks were highest for CVD/CHD in men, while CVD/CHD and ARC risks were of similar magnitude in women. In general, as individuals reporting a prevalent condition at recruitment (either cancer, diabetes, heart attack or stroke) were excluded from the analysis in an effort to minimize reverse causality, our estimates of absolute risks of death are possibly underestimated. RAP values were estimated to appreciate

For peer review only

the risk benefit of alcohol drinkers if they were to modify their exposure, according to a counterfactual scenario (21;33). Consistently in men and women, RAP values for overall mortality were larger when the reference category was set to 0.1 to 15 g/day than when using a threshold of 5 g/day, thus indicating that, based on the EPIC study, the benefit for drinkers could be largest if their intake is reduced to levels below 15 g/day. These results are in line with findings of a recent work in the UK population, where the reduction of overall mortality was estimated to be optimized for alcohol reduction up to a median population level of 5 g/day (34).

In a large prospective study in Europe, lifetime alcohol intake was significantly associated with both overall and ARC specific mortality. In men, positive associations were observed for violent deaths and injuries, while CVD and CHD deaths were not associated with alcohol use among drinkers. Our findings suggest that these associations do not differ between never and current smokers, and were stronger for beer than for wine drinkers.

For peer review only

Funding statement

This work was supported by the Direction Générale de la Santé (French Ministry of Health) (Grant GR-IARC-2003-09-12-01), by the European Commission (Directorate General for Health and Consumer Affairs) and the International Agency for Research on Cancer. The national cohorts are supported by the Danish Cancer Society (Denmark); the Ligue Contre le Cancer, the Institut Gustave Roussy, the Mutuelle Générale de l'Education Nationale, and the Institut National de la Santé et de la Recherche Médicale (France); the Deutsche Krebshilfe, the Deutsches Krebsforschungszentrum, and the Federal Ministry of Education and Research (Germany); the Hellenic Health Foundation, the Stavros Niarchos Foundation, and the Hellenic Ministry of Health and Social Solidarity (Greece); the Italian Association for Research on Cancer and the National Research Council (Italy); the Dutch Ministry of Public Health, Welfare and Sports, the Netherlands Cancer Registry, LK Research Funds, Dutch Prevention Funds, the Dutch Zorg Onderzoek Nederland, the World Cancer Research Fund, and Statistics Netherlands (Netherlands); European Research Council-2009-AdG 232997 and the Nordforsk, Nordic Centre of Excellence program on Food, Nutrition and Health (Norway); the Health Research Fund, Regional Governments of Andalucýa, Asturias, Basque Country, Murcia (project 6236) and Navarra, Instituto de Salud Carlos III, Redes de Investigacion Cooperativa (RD06/0020) (Spain); the Swedish Cancer Society, the Swedish Scientific Council, and the Regional Government of Skåne (Sweden); Cancer Research UK, the Medical Research Council, the Stroke Association, the British Heart Foundation, the Department of Health, the Food Standards Agency, and the Wellcome Trust (United Kingdom). The work undertaken by David C Muller was done during the tenure of an IARC, Australia postdoctoral fellowship, supported by the Cancer Council Australia.

Contributorship statement

Pietro FERRARI, Idlir LICAJ, Isabelle ROMIEU, Paul BRENNAN, Heiner BOEING conceptualized the study and defined the analytical strategy. Pietro FERRARI and Idlir LICAJ had full access to all the data in the study and take responsibility for the integrity of the data and the accuracy of the data analysis. Pietro FERRARI, Idlir LICAJ, David C MULLER, Per Kragh ANDERSEN, Luciana NUNES performed statistical analyses and provided preliminary interpretation of findings. Pietro FERRARI, David C MULLER, Mattias JOHANSSON, Idlir LICAJ, Paul BRENNAN contributed to draft the manuscript. Elisabete WEIDERPASS, Laure DOSSUS, Laureen DARTOIS, Guy FAGHERAZZI,

For peer review only

Kathryn Erica BRADBURY, Kay-Tee KHAW, Eric Jeffrey DUELL, Aurelio BARRICARTE, Esther MOLINA-MONTES, Carmen NAVARRO SANCHEZ, Larraitz ARRIOLA, Peter WALLSTRÖM, Anne TJØNNELAND, Anja OLSEN, Antonia TRICHOPOULOU, Vasiliki BENETOU, Dimitrios TRICHOPOULOS, Rosario TUMINO, Claudia AGNOLI, Carlotta SACERDOTE, Domenico PALLI, Kuanrong LI, Rudolf KAAKS, Petra PEETERS, Joline Wilhelma Johanna BEULENE, Marc GUNTER, Teresa NORAT, Kim OVERVAD, Elio RIBOLI, Paul BRENNAN, and Isabelle ROMIEU played a key role in the acquisition of the data, and were active in searching for funding to continue the study. With respect to this work, they all critically helped in the interpretation of results, revised the manuscript and provided relevant intellectual input.

Competing Interest Declaration

All authors have completed the Unified Competing Interest form at www.icmje.org/coi_disclosure.pdf (available on request from the corresponding author). Dr Kathryn E BRADBURY reports grants from Cancer Research UK, during the conduct of the study. The rest of the authors declared no support from any organisation for the submitted work, no financial relationships with any organisations that might have an interest in the submitted work in the previous three years, no other relationships or activities that could appear to have influenced the submitted work.

Sharing statement

Statistical code is available from the corresponding author by emailing ferrarip@iarc.fr.

For peer review only

Reference List

(1) Thun MJ, Peto R, Lopez AD, et al. Alcohol consumption and mortality among middle-aged and elderly U.S. adults. N Engl J Med 1997 Dec 11;337(24):1705-14.

(2) Bagnardi V, Blangiardo M, La Vecchia C., et al. A meta-analysis of alcohol drinking and cancer risk. Br J Cancer 2001 Nov 30;85(11):1700-5.

(3) Rehm J, Baliunas D, Borges GL, et al. The relation between different dimensions of alcohol consumption and burden of disease: an overview. Addiction 2010 May;105(5):817-43.

(4) Corrao G, Bagnardi V, Zambon A, et al. A meta-analysis of alcohol consumption and the risk of 15 diseases. Prev Med 2004 May;38(5):613-9.

(5) Ronksley PE, Brien SE, Turner BJ, et al. Association of alcohol consumption with selected cardiovascular disease outcomes: a systematic review and meta-analysis. BMJ 2011;342:d671.

(6) IARC. Alcohol consumption and ethyl carbamate. 96 ed. IARC; 2010.

(7) WCRF/AICR. Food, nutrition, physical activity and the prevention of cancer: a global perspective. Washington DC: AICR; 2007.

(8) Rehm J, Mathers C, Popova S, et al. Global burden of disease and injury and economic cost attributable to alcohol use and alcohol-use disorders. Lancet 2009 Jun 27;373(9682):2223-33.

(9) Lim SS, Vos T, Flaxman AD, et al. A comparative risk assessment of burden of disease and injury attributable to 67 risk factors and risk factor clusters in 21 regions, 1990-2010: a systematic analysis for the Global Burden of Disease Study 2010. Lancet 2012 Dec 15;380(9859):2224-60.

(10) Bergmann MM, Rehm J, Klipstein-Grobusch K, et al. The association of pattern of lifetime alcohol use and cause of death in the European Prospective Investigation into Cancer and Nutrition (EPIC) study. Int J Epidemiol 2013 Dec;42(6):1772-90.

(11) Riboli E, Hunt KJ, Slimani N, et al. European Prospective Investigation into Cancer and Nutrition (EPIC): study populations and data collection. Public Health Nutr 2002 Dec;5(6B):1113-24.

(12) Ferrari P, Jenab M, Norat T, et al. Lifetime and baseline alcohol intake and risk of colon and rectal cancers in the European prospective investigation into cancer and nutrition (EPIC). Int J Cancer 2007 Nov 1;121(9):2065-72.

(13) Ferrari P, Day NE, Boshuizen HC, et al. The evaluation of the diet/disease relation in the EPIC study: considerations for the calibration and the disease models. Int J Epidemiol 2008 Apr;37(2):368-78.

(14) Royston P, Parmar MK. The use of restricted mean survival time to estimate the treatment effect in randomized clinical trials when the proportional hazards assumption is in doubt. Stat Med 2011 Aug 30;30(19):2409-21.

For peer review only

(15) Heinzl H, Kaider A. Gaining more flexibility in Cox proportional hazards regression models with cubic spline functions. Comput Methods Programs Biomed 1997 Nov;54(3):201-8.

(16) Sauerbrei W, Royston P. Building multivariable prognostic and diagnostic models: Transformation of the predictors by using fractional polynomials. Journal ofthe Royal Statistical Society, Series A 1999;62:71-94.

(17) Royston P, Ambler G, Sauerbrei W. The use of fractional polynomials to model continuous risk variables in epidemiology. Int J Epidemiol 1999 Oct;28(5):964-74.

(18) Andersen PK, Geskus RB, de WT, et al. Competing risks in epidemiology: possibilities and pitfalls. Int J Epidemiol 2012 Jun;41(3):861-70.

(19) Putter H, Fiocco M, Geskus RB. Tutorial in biostatistics: competing risks and multi-state models. Stat Med 2007 May 20;26(11):2389-430.

(20) Rosthoj S, Andersen PK, Abildstrom SZ. SAS macros for estimation of the cumulative incidence functions based on a Cox regression model for competing risks survival data. Comput Methods Programs Biomed 2004 Apr;74(1):69-75.

(21) Brenner H, Gefeller O, Greenland S. Risk and rate advancement periods as measures of exposure impact on the occurrence of chronic diseases. Epidemiology 1993 May;4(3):229-36.

(22) SAS Institute Inc. 2002. NC, USA, Cary.

(23) Stata Statistical Software: *Release 12*. Version StataCorp. College Station, TX: StataCorp LP.: 2011.

(24) Zaridze D, Lewington S, Boroda A, et al. Alcohol and mortality in Russia: prospective observational study of 151 000 adults. Lancet 2014 Jan 30;(13):10-6736.

(25) Thun MJ, Peto R, Lopez AD, et al. Alcohol consumption and mortality among middle-aged and elderly U.S. adults. N Engl J Med 1997 Dec 11;337(24):1705-14.

(26) Lin Y, Kikuchi S, Tamakoshi A, et al. Alcohol consumption and mortality among middle-aged and elderly Japanese men and women. Ann Epidemiol 2005 Sep;15(8):590-7.

(27) Fillmore KM, Stockwell T, Chikritzhs T, et al. Moderate alcohol use and reduced mortality risk: systematic error in prospective studies and new hypotheses. Ann Epidemiol 2007 May;17(5 Suppl):S16-S23.

(28) Naimi TS, Brown DW, Brewer RD, et al. Cardiovascular risk factors and confounders among nondrinking and moderate-drinking U.S. adults. Am J Prev Med 2005 May;28(4):369-73.

(29) Klatsky AL, Friedman GD, Armstrong MA, et al. Wine, liquor, beer, and mortality. Am J Epidemiol 2003 Sep 15;158(6):585-95.

For peer review only

(30) Maskarinec G, Meng L, Kolonel LN. Alcohol intake, body weight, and mortality in a multiethnic prospective cohort. Epidemiology 1998 Nov;9(6):654-61.

(31) Gronbaek M, Becker U, Johansen D, et al. Type of alcohol consumed and mortality from all causes, coronary heart disease, and cancer. Ann Intern Med 2000 Sep;19;133(6):411-9.

(32) Renaud SC, Gueguen R, Siest G, et al. Wine, beer, and mortality in middle-aged men from eastern France. Arch Intern Med 1999 Sep 13;159(16):1865-70.

(33) Gellert C, Schottker B, Holleczek B, et al. Using rate advancement periods for communicating the benefits of quitting smoking to older smokers. Tob Control 2013 Jul;22(4):227-30.

(34) Nichols M, Scarborough P, Allender S, et al. What is the optimal level of population alcohol consumption for chronic disease prevention in England? Modelling the impact of changes in average consumption levels. BMJ Open 2012 May 30;2(3):e000957.

For peer review only

Table 1. Country and sex-specific number of participants (N), person-years (PY), cause-specific and overall number of deaths.

Country	N	PY	CHD[1]	CVD[2]	Cancers					Other cancers[5]	Violent & Injuries[6]	Resp[7]	Other causes[8]	Total
					Breast	UADT[3]	Liver	Colon-rectum	Total[4]					
Women														
France	65,127	971,127	45	202	62	8	4	27	101	678	115	73	1,619	2,833
Italy	24,956	306,244	26	87	71	6	12	53	142	293	30	12	120	710
Spain	23,616	323,027	41	50	51	6	9	47	113	243	46	9	119	621
UK	50,251	651,640	387	320	160	30	15	127	332	716	85	161	1,091	3,092
Netherlands	14,583	189,531	110	137	58	14	7	78	157	370	20	65	209	1,068
Greece	14,391	143,150	139	100	41	2	10	18	71	146	26	27	94	603
Germany	27,098	307,380	56	74	64	12	14	44	134	256	35	25	114	694
Denmark	27,773	328,375	84	143	128	23	17	110	278	648	51	116	548	1,868
All	247,795	3,220,474	888	1,113	635	101	88	504	1,328	3,335	408	488	3,848	11,489
Men														
France	-	-	-	-	-	-	-	-	-	-	-	-	-	-
Italy	13,471	168,992	53	59	-	8	10	39	57	240	35	13	131	588
Spain	14,089	189,942	136	88	-	34	14	62	110	351	81	42	151	959
UK	20,452	262,720	438	229	-	41	11	68	120	567	88	171	1,040	2,653
Netherlands	-	-	-	-	-	-	-	-	-	-	-	-	-	-
Greece	9,726	90,989	193	141	-	12	20	31	63	279	49	54	99	878
Germany	19,743	221,724	167	138	-	37	25	65	127	437	84	35	264	1,253
Denmark	24,454	282,622	273	271	-	91	31	126	248	838	111	95	794	2,633
All	101,935	1,216,988	1,260	926	-	223	111	391	725	2,712	448	410	2,479	8,964

[1]CHD: Coronary heart disease [I20-I25] deaths; [2]CVD: Cardiovascular disease [I00-I99 except I20-I25] deaths; [3]UADT: deaths from upper aero-digestive cancers (including cancer of the mouth [C01 to C10 without C08=salivary gland]), larynx [C21], pharynx [C11 to C14], oesophagus [C15]); [4]Total frequency of alcohol related cancers; [5]Other cancers: deaths from all other cancers; [6]Violent deaths and injuries, including injury, poisoning and certain other consequences of external causes [S00-T98], and external causes of morbidity and mortality [V01-Y98]; [7]Resp=respiratory diseases [J00-J99].

Table 2. Characteristics of the study population at recruitment, according to amount and type of alcohol intake (g/day) in the EPIC study[1].

Characteristics	Unit	Never drinkers	Lifetime drinkers					Total[3]	Wine consumers[4]	Beer consumers[4]
			0.1-4.9	5-14.9	15-29.9	30-59.9[2]	>60[2]			
Women										
Number of participants	n	25,146	112,281	77,147	27,179	6,042	-	247,795	85,965	8,748
Person-Years	-	330,854	1,460,315	998,547	352,220	78,538	-	3,220,474	1,124,546	110,761
Age at recruitment	Years	52 (9)	52 (10)	51 (10)	49 (11)	47 (11)	-	51 (38-63)	52 (9)	46 (12)
Lifetime alcohol intake	g/day	0 (-)	2 (2)	9 (3)	20 (4)	43 (21)	-	7 (0-17)	12 (9)	11 (9)
Educational attainment[5]	%	14	22	27	33	37	-	25	28	28
Current smokers	%	13	14	18	24	31	-	17	17	28
Body mass index	kg/m^2	27 (5)	25 (5)	25 (4)	24 (4)	24 (4)	-	25 (20-31)	24 (4)	25 (4)
Height	cm	158 (6)	161 (6)	162 (6)	163 (7)	164 (6)	-	162 (153-170)	162 (6)	163 (7)
(Moderately) Active	%	26	39	44	44	43	-	40	42	42
Ever use of HRT[6]	%	16	25	29	28	25	-	25	50	34
Post-menopausal status[7]	%	50	49	49	44	38	-	48	29	20
Energy intake	kcal/day	1,848 (537)	1,943 (537)	2,015 (536)	2,090 (552)	2,195 (602)	-	1978 (542)	2,046 (544)	1,976 (545)
Men										
Number of participants	n	1,600	14,287	28,875	28,049	20,788	8,336	101,935	26,137	22,136
Person-Years	-	19,114	171,739	345,899	333,784	247,612	98,841	1,216,989	317,937	259,934
Age at recruitment	Years	53 (11)	53 (11)	53 (9)	52 (9)	52 (9)	52 (9)	53 (41-64)	53 (9)	52 (10)
Lifetime alcohol intake	g/day	0 (-)	2 (2)	10 (3)	22 (4)	42 (8)	94 (45)	25 (3-45)	30 (27)	22 (25)
Educational attainment[5]	%	21	30	31	32	26	14	29	22	27
Current smokers	%	28	22	25	30	36	49	30	31	33
Body mass index	kg/m^2	27 (4)	26 (4)	26 (3)	27 (3)	27 (4)	28 (4)	27 (22-31)	27 (4)	27 (4)
Height	cm	171 (7)	174 (7)	175 (7)	175 (7)	174 (7)	172 (7)	174 (165-183)	172 (7)	175 (7)
(Moderately) Active	%	42	46	50	52	52	50	50	48	52
Energy intake	kcal/day	2,284 (675)	2,267 (650)	2,315 (618)	2,417 (622)	2,569 (646)	2,789 (716)	2,427 (656)	2,487 (652)	2,369 (651)

[1]Means ± SDs are presented for continuous variables, frequencies for categorical variables; [2]In women the last alcohol category is ≥30g/day; [3]For continuous variables (with exception of energy intake), mean (10th-90th percentile) values are reported; [4]Study subjects consuming more than 10 g/day of wine (or beer), and consuming less than 3 g/day of beer (or wine); [5]Subjects with a university degree or more; [6]HRT=hormonal replacement therapy; [7]Post-menopausal women plus women who underwent an ovariectomy.

For peer review only

Figure 1. Number of deaths, person-years (PY) and multivariable hazard ratios[1] (HR) with 95% confidence intervals (CI) and p-value of the Wald test for statistical significance for overall and cause-specific mortality by categories of lifetime alcohol use, in women and men.

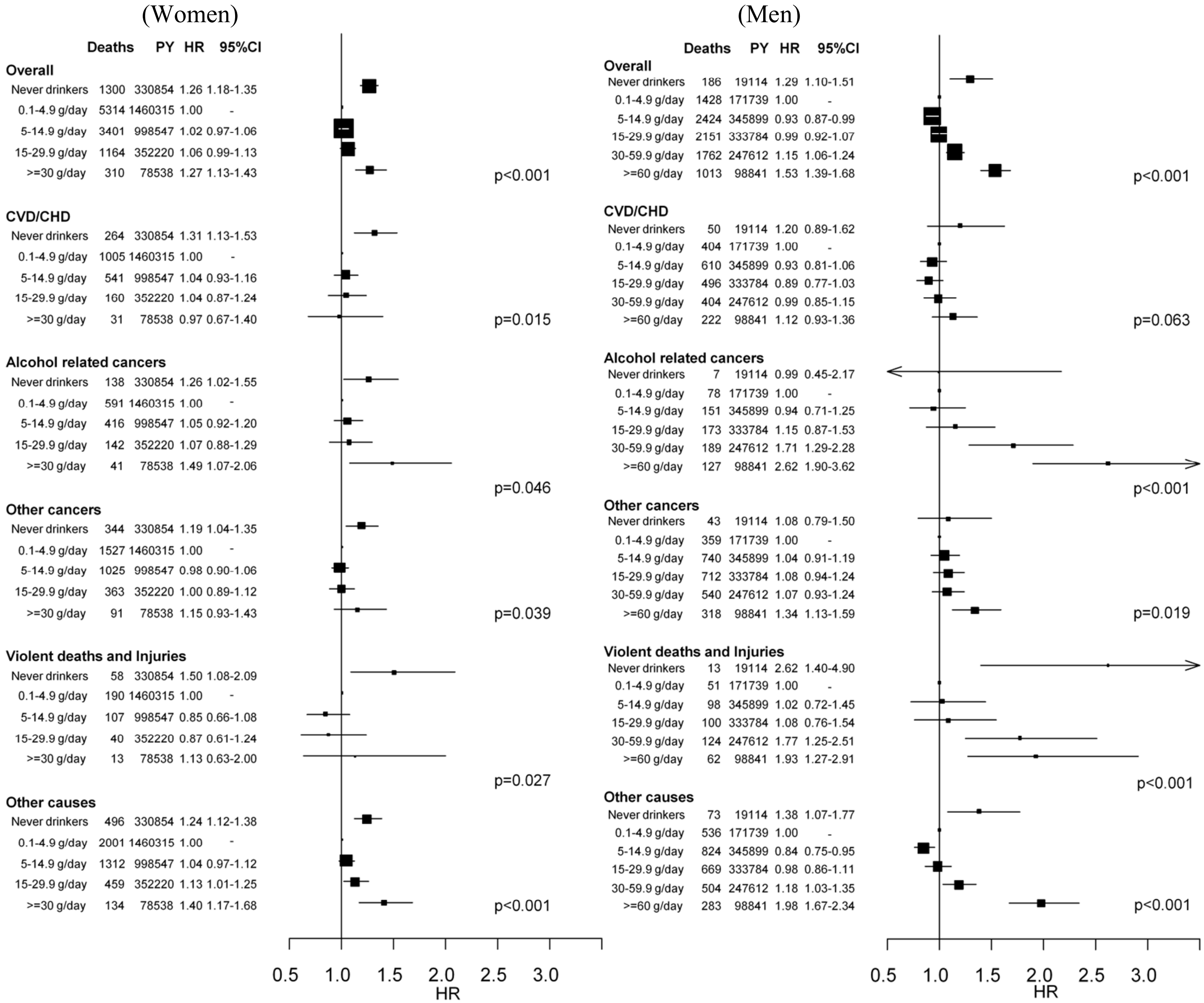

(Women)

	Deaths	PY	HR	95%CI	p
**Overall**					
Never drinkers	1300	330854	1.26	1.18-1.35	
0.1-4.9 g/day	5314	1460315	1.00	-	
5-14.9 g/day	3401	998547	1.02	0.97-1.06	
15-29.9 g/day	1164	352220	1.06	0.99-1.13	
>=30 g/day	310	78538	1.27	1.13-1.43	p<0.001
**CVD/CHD**					
Never drinkers	264	330854	1.31	1.13-1.53	
0.1-4.9 g/day	1005	1460315	1.00	-	
5-14.9 g/day	541	998547	1.04	0.93-1.16	
15-29.9 g/day	160	352220	1.04	0.87-1.24	
>=30 g/day	31	78538	0.97	0.67-1.40	p=0.015
**Alcohol related cancers**					
Never drinkers	138	330854	1.26	1.02-1.55	
0.1-4.9 g/day	591	1460315	1.00	-	
5-14.9 g/day	416	998547	1.05	0.92-1.20	
15-29.9 g/day	142	352220	1.07	0.88-1.29	
>=30 g/day	41	78538	1.49	1.07-2.06	p=0.046
**Other cancers**					
Never drinkers	344	330854	1.19	1.04-1.35	
0.1-4.9 g/day	1527	1460315	1.00	-	
5-14.9 g/day	1025	998547	0.98	0.90-1.06	
15-29.9 g/day	363	352220	1.00	0.89-1.12	
>=30 g/day	91	78538	1.15	0.93-1.43	p=0.039
**Violent deaths and Injuries**					
Never drinkers	58	330854	1.50	1.08-2.09	
0.1-4.9 g/day	190	1460315	1.00	-	
5-14.9 g/day	107	998547	0.85	0.66-1.08	
15-29.9 g/day	40	352220	0.87	0.61-1.24	
>=30 g/day	13	78538	1.13	0.63-2.00	p=0.027
**Other causes**					
Never drinkers	496	330854	1.24	1.12-1.38	
0.1-4.9 g/day	2001	1460315	1.00	-	
5-14.9 g/day	1312	998547	1.04	0.97-1.12	
15-29.9 g/day	459	352220	1.13	1.01-1.25	
>=30 g/day	134	78538	1.40	1.17-1.68	p<0.001

(Men)

	Deaths	PY	HR	95%CI	p
**Overall**					
Never drinkers	186	19114	1.29	1.10-1.51	
0.1-4.9 g/day	1428	171739	1.00	-	
5-14.9 g/day	2424	345899	0.93	0.87-0.99	
15-29.9 g/day	2151	333784	0.99	0.92-1.07	
30-59.9 g/day	1762	247612	1.15	1.06-1.24	
>=60 g/day	1013	98841	1.53	1.39-1.68	p<0.001
**CVD/CHD**					
Never drinkers	50	19114	1.20	0.89-1.62	
0.1-4.9 g/day	404	171739	1.00	-	
5-14.9 g/day	610	345899	0.93	0.81-1.06	
15-29.9 g/day	496	333784	0.89	0.77-1.03	
30-59.9 g/day	404	247612	0.99	0.85-1.15	
>=60 g/day	222	98841	1.12	0.93-1.36	p=0.063
**Alcohol related cancers**					
Never drinkers	7	19114	0.99	0.45-2.17	
0.1-4.9 g/day	78	171739	1.00	-	
5-14.9 g/day	151	345899	0.94	0.71-1.25	
15-29.9 g/day	173	333784	1.15	0.87-1.53	
30-59.9 g/day	189	247612	1.71	1.29-2.28	
>=60 g/day	127	98841	2.62	1.90-3.62	p<0.001
**Other cancers**					
Never drinkers	43	19114	1.08	0.79-1.50	
0.1-4.9 g/day	359	171739	1.00	-	
5-14.9 g/day	740	345899	1.04	0.91-1.19	
15-29.9 g/day	712	333784	1.08	0.94-1.24	
30-59.9 g/day	540	247612	1.07	0.93-1.24	
>=60 g/day	318	98841	1.34	1.13-1.59	p=0.019
**Violent deaths and Injuries**					
Never drinkers	13	19114	2.62	1.40-4.90	
0.1-4.9 g/day	51	171739	1.00	-	
5-14.9 g/day	98	345899	1.02	0.72-1.45	
15-29.9 g/day	100	333784	1.08	0.76-1.54	
30-59.9 g/day	124	247612	1.77	1.25-2.51	
>=60 g/day	62	98841	1.93	1.27-2.91	p<0.001
**Other causes**					
Never drinkers	73	19114	1.38	1.07-1.77	
0.1-4.9 g/day	536	171739	1.00	-	
5-14.9 g/day	824	345899	0.84	0.75-0.95	
15-29.9 g/day	669	333784	0.98	0.86-1.11	
30-59.9 g/day	504	247612	1.18	1.03-1.35	
>=60 g/day	283	98841	1.98	1.67-2.34	p<0.001

[1]Models were stratified by center. Systematic adjustment was undertaken for age at recruitment, BMI and height, former drinking, time since alcohol quitting, smoking status, duration of smoking, age at start smoking, educational attainment, and energy intake. In women adjustment was undertaken for menopausal status, ever use of replacement hormones, and number of full term pregnancies.

Table 3. Sex-specific number of deaths, hazard ratios (HR)[1] and 95%CI for overall mortality by categories of lifetime alcohol use (g/day), by smoking status (never and current smokers), and type of alcoholic beverage.

Women

	Never smokers			Current smokers			
	deaths	HR[2]	(95% CI)	deaths	HR[2]	(95% CI)	$p_{heterog}$[3]
**Overall**							
Never	1,009	1.34	(1.24, 1.45)	154	1.72	(1.32, 2.23)	
0.1-4.9	3,046	1	Ref	1,021	1.53	(1.23, 1.90)	
5-14.9	1,550	1.04	(0.98, 1.11)	874	1.51	(1.21, 1.88)	
15-29.9	397	1.04	(0.94, 1.16)	435	1.74	(1.38, 2.19)	
≥ 30	82	1.29	(1.03, 1.61)	140	2.08	(1.59, 2.73)	
p_{Wald}[4]			<0.001			<0.001	0.150

	Wine use			Beer use			
	deaths	HR[5]	(95% CI)	deaths	HR[5]	(95% CI)	$p_{difference}$[6]
**Overall**							
Never	2,156	1.15	(1.09, 1.22)	5,041	1.06	(1.02, 1.12)	
0.1-2.9	5,109	1	Ref	5,477	1	Ref	
3-9.9	2,813	0.96	(0.92, 1.01)	787	1.15	(1.07, 1.24)	
10-19.9	1,057	1.00	(0.93, 1.07)	147	1.50	(1.27, 1.77)	
≥ 20	354	1.14	(1.02, 1.27)	37	1.47	(1.06, 2.04)	
p_{Wald}[4]			<0.001			<0.001	<0.001

Men

	Never smokers			Current smokers			
	deaths	HR[2]	(95% CI)	deaths	HR[2]	(95% CI)	$p_{heterog}$[3]
**Overall**							
Never	84	1.50	(1.19, 1.21)	58	2.09	(1.26, 3.47)	
0.1-4.9	457	1	Ref	367	1.62	(1.04, 2.53)	
5-14.9	538	0.93	(0.82, 1.06)	799	1.45	(0.93, 2.25)	
15-29.9	369	1.00	(0.87, 1.16)	927	1.66	(1.06, 2.58)	
30-59.9	254	1.22	(1.23, 1.43)	857	1.83	(1.17, 2.84)	
≥ 60	107	1.56	(1.25, 1.95)	590	2.43	(1.55, 3.80)	
p_{Wald}[4]			<0.001			<0.001	0.864

	Wine use			Beer use			
	deaths	HR[5]	(95% CI)	deaths	HR[5]	(95% CI)	$p_{difference}$[6]
**Overall**							
Never	1,064	1.21	(1.12, 1.30)	975	1.07	(0.98, 1.16)	
0.1-2.9	3,266	1	Ref	2,959	1	Ref	
3-9.9	2,139	0.92	(0.87, 0.97)	2,486	1.04	(0.98, 1.10)	
10-19.9	1,040	0.96	(0.89, 1.03)	1,248	1.12	(1.04, 1.20)	
20-39.9	814	1.03	(0.95, 1.13)	877	1.41	(1.30, 1.54)	
≥ 40	641	1.22	(1.10, 1.35)	419	1.86	(1.66, 2.09)	
p_{Wald}[4]			<0.001			<0.001	<0.001

[1]Models were stratified by center. Systematic adjustment was undertaken for age at recruitment, BMI and height, former drinking, time since alcohol quitting, smoking status, duration of smoking, age at start smoking, educational attainment, and energy intake. In women adjustment was undertaken for menopausal status, ever use of replacement hormones, and number of full term pregnancies; [2]Models included interaction terms between lifetime alcohol use and a smoking indicator (0=never smokers; 1=current smokers), keeping the reference category the group of moderate alcohol users (0.1-4.9 g/day) among never smokers, whereas former smokers and subjects with unknown smoking status were excluded; [3]$p_{heterogeneity}$: difference in HRs assessed comparing the log-likelihood of models with and without interaction terms between alcohol and smoking status to a 4 and 5 degrees of freedom (dof) chi-square distribution, in women and men respectively; [4]p_{Wald}: determined using a Wald test for contrasts according to a chi-square distribution with 4 and 5 dof, in women and men respectively; [5]Models on wine and beer uses were mutually adjusted, and also included spirits/liquors use; [6]$p_{difference}$ expresses the difference of associations between wine and beer use, determined evaluating the significance of the parameter estimate γ_2 in a model that included, other than the list of confounders, the terms $\gamma_1 \cdot (X_1+X_2)/2 + \gamma_2 \cdot (X_1-X_2)/2$, with X_1=log(wine use+1) and X_2=log(beer use+1).

For peer review only

Figure 2. Sex-specific plots displaying cumulative probabilities of death due to overall mortality, for heavy (≥30 g/day in women and ≥60 g/day in men, *continuous* line) and moderate lifetime use (0.1-4.9 g/day) (*dotted* line), in smokers (*black* line) and never smokers (*grey* line), for study subjects aged 60 years.

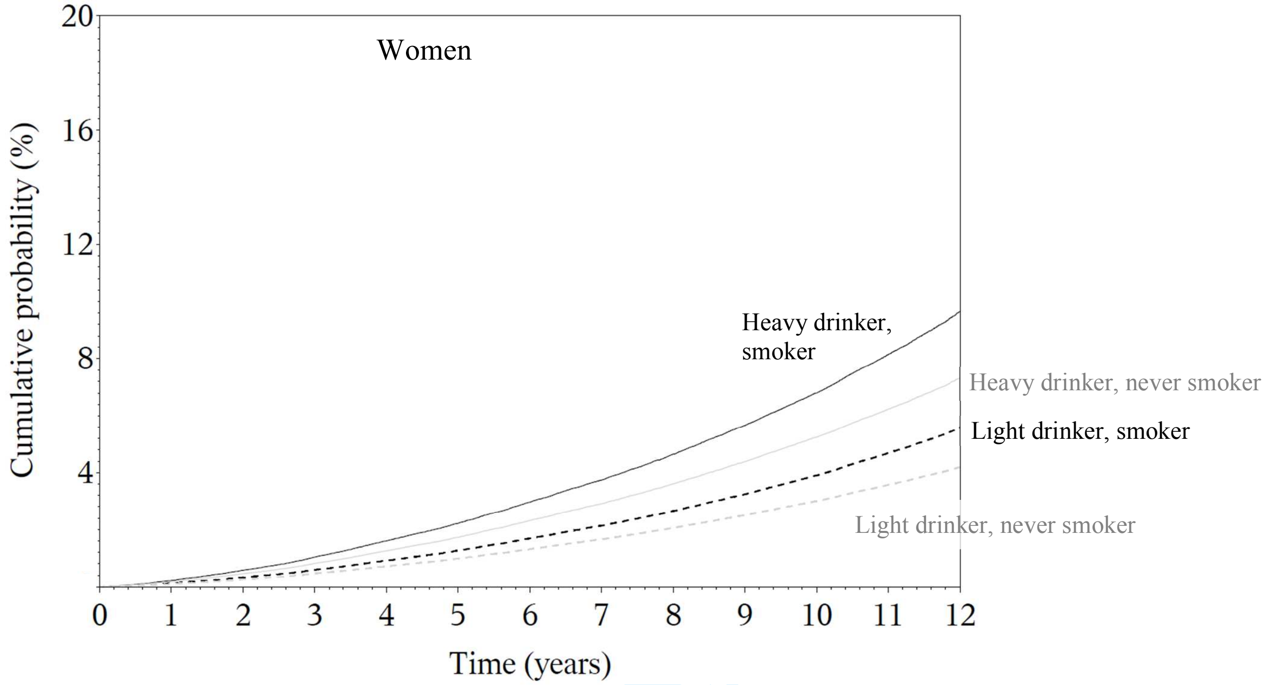

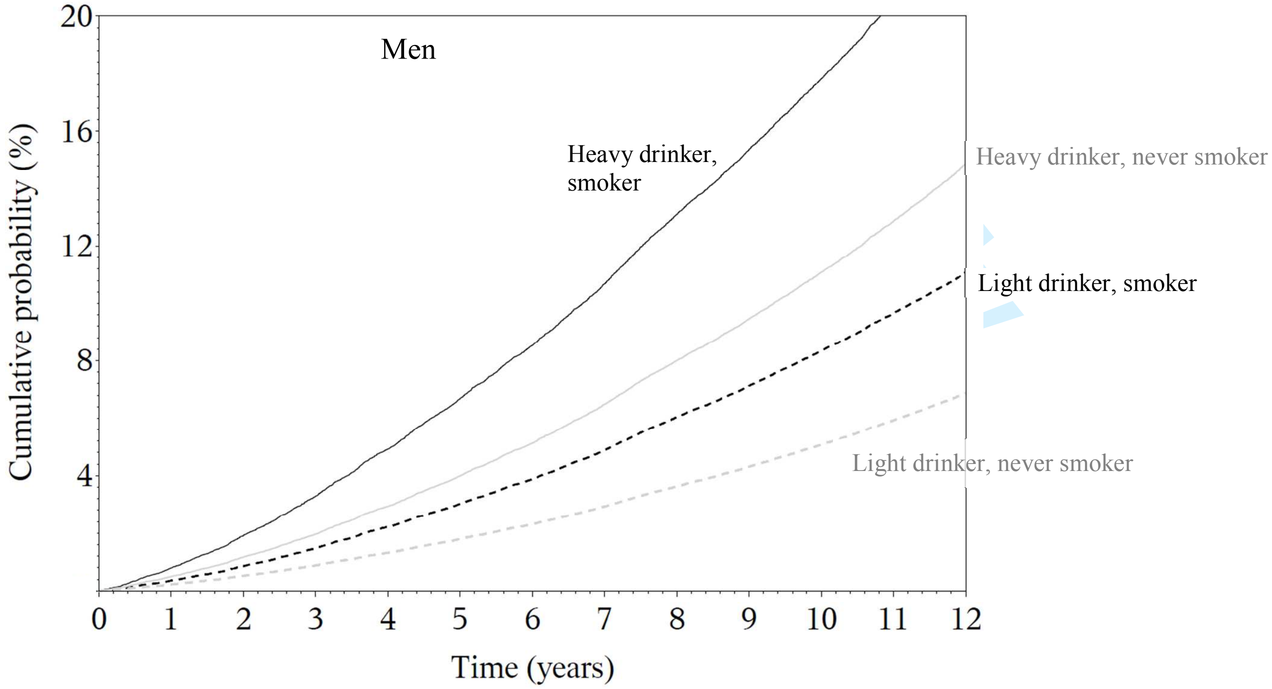

Figure 3. In competing risks analyses, sex-specific plots displaying cumulative probabilities of death due to CVD/CHD (red), alcohol related cancers (blue), and violent death and injuries (green), for study subjects aged 60 years according to heavy (≥30 g/day in women and ≥60 g/day in men, *continuous* line) and moderate (0.1-4.9 g/day, *dotted* lines) lifetime alcohol use in current and never smokers in the EPIC study.

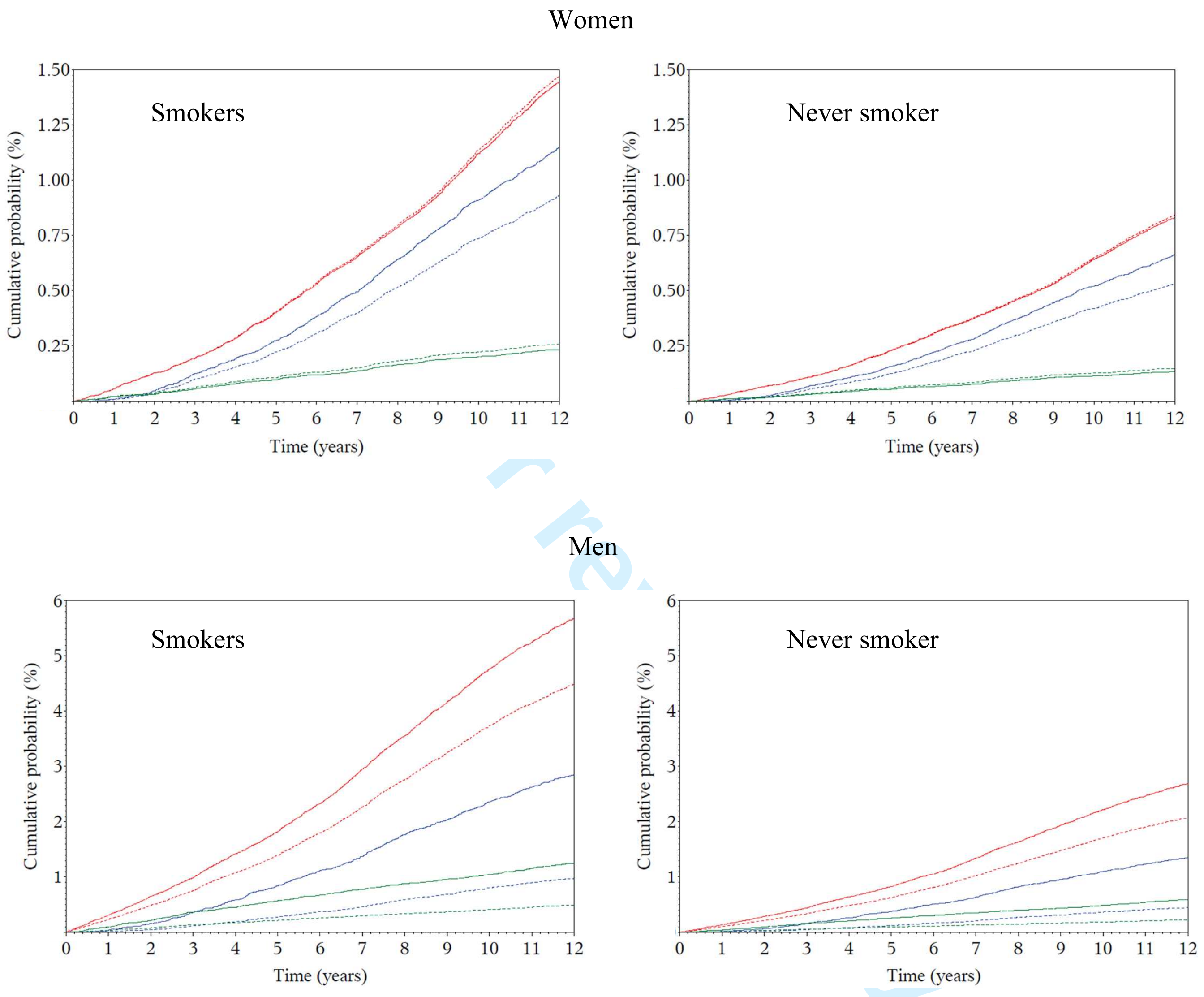

Table 4. Sex-specific estimates of rate advancement period (RAP) and associated 95%CI for overall and mortality due to ARCs, CVD/CHD and injuries and violent deaths, related to two scenarios of lifetime alcohol use. RAP estimates express the impact of a given exposure on the risk of death, by determining the time (in years) by which the risk of death is anticipated for study subjects exposed, e.g. all drinkers more than the threshold (5 or 15 g/day in Scenario I and II, respectively), compared to non-exposed, i.e. individuals drinking between 0.1 g/day and the threshold.

		Scenario I		Scenario II	
		Threshold: 5 g/day		Threshold: 15 g/day	
		RAP (years)	95%CI	RAP (years)	95%CI
Women	Overall	0.36	-0.05, 0.76	0.83	0.26, 1.39
	CVD/CHD	0.23	-0.46, 0.92	0.08	-0.96, 1.14
	Alcohol related cancers	1.28	-0.86, 3.41	1.90	-1.00, 4.81
	Injuries and violent deaths	-2.69	-6.85, 1.47	-0.20	-5.85, 5.46
Men	Overall	0.15	-0.48, 0.76	1.42	0.96, 1.89
	CVD/CHD	-0.53	-1.57, 0.50	-0.01	-0.82, 0.81
	Alcohol related cancers	2.59	-0.30, 5.49	5.03	3.07, 7.00
	Injuries and violent deaths	7.59	-2.82, 18.02	11.83	3.92, 18.17

[1]Never lifetime alcohol users did not enter into the estimation process.

For peer review only

Lifetime alcohol use and overall and cause-specific mortality in the European Prospective Investigation into Cancer and nutrition (EPIC) study.

Pietro FERRARI[1], Idlir LICAJ[1], David C MULLER[1], Per Kragh ANDERSEN[2], Mattias JOHANSSON[1], Heiner BOEING[3], Elisabete WEIDERPASS[4,5,6,7], Laure DOSSUS[8,9,10], Laureen DARTOIS[8,9,10], Guy FAGHERAZZI[8,9,10], Kathryn E BRADBURY[11], Kay-Tee KHAW[12], Nick WAREHAM[13], Eric J DUELL[14], Aurelio BARRICARTE[15,16], Esther MOLINA-MONTES[17,18], Carmen NAVARRO SANCHEZ[18,19,20], Larraitz ARRIOLA[16,21], Peter WALLSTRÖM[22], Anne TJØNNELAND[23], Anja OLSEN[23], Antonia TRICHOPOULOU[24,25], Vasiliki BENETOU[24], Dimitrios TRICHOPOULOS[25,26,27], Rosario TUMINO[28], Claudia AGNOLI[29], Carlotta SACERDOTE[30,31], Domenico PALLI[32], Kuanrong LI[33], Rudolf KAAKS[33], Petra PEETERS[34], Joline WJ BEULENS[34], Luciana NUNES[1,35], Marc GUNTER[36], Teresa NORAT[36], Kim OVERVAD[37,38], Paul BRENNAN[1], Elio RIBOLI[36], Isabelle ROMIEU[1].

Author Affiliations:

[1]International Agency for Research on Cancer, Lyon, France.

[2]Department of Biostatistics, University of Copenhagen, Copenhagen, Denmark.

[3]German Institute of Human Nutrition, Potsdam-Rehbrücke Department of Epidemiology, Nuthetal, Germany.

[4]Department of Community Medicine, Faculty of Health Sciences, University of Tromso, The Arctic University of Norway, Tromsø, Norway.

[5]Department of Research, Cancer Registry of Norway, Oslo, Norway.

[6]Department of Medical Epidemiology and Biostatistics, Karolinska Institutet, Stockholm, Sweden.

[7]Samfundet Folkhälsan, Helsinki, Finland.

[8]Inserm, Centre for research in Epidemiology and Population Health (CESP), U1018, Nutrition, Hormones and Women's Health team, F-94805, Villejuif, France.

[9]Université Paris Sud, UMRS 1018, F-94805, Villejuif, France.

[10]IGR, F-94805, Villejuif, France.

[11]Cancer Epidemiology Unit, Nuffield Department of Population Health, University of Oxford, UK.

For peer review only

[12]the Department of Public Health and Primary Care, University of Cambridge Addenbrooke's Hospital, Cambridge, United Kingdom.

[13]the Medical Research Council Epidemiology Unit, Institute of Metabolic Science, Addenbrooke's Hospital, Cambridge, United Kingdom;

[14]Unit of Nutrition, Environment and Cancer, Cancer Epidemiology Research Program Bellvitge Biomedical Research Institute (IDIBELL), Catalan Institute of Oncology (ICO), Barcelona, Spain.

[15]Navarre Public Health Institute, Pamplona, Spain.

[16]Consortium for Biomedical Research in Epidemiology and Public Health (CIBER Epidemiología y Salud Pública-CIBERESP), Spain.

[17]Escuela Andaluza de Salud Pública. Instituto de Investigación Biosanitaria de Granada, Granada, Spain.

[18]CIBER Epidemiología y Salud Pública (CIBERESP), Spain.

[19]Department of Epidemiology, Murcia Regional Health Council, Murcia, Spain.

[20]Department of Health and Social Sciences, Universidad de Murcia, Spain.

[21]Public Health Division of Gipuzkoa, Instituto BIO-Donostia, Basque Government. Spain.

[22]Nutrition Epidemiology Research Group, Department of Clinical Sciences, Lund University, Malmö, Sweden.

[23]Danish Cancer Society Research Center, Copenhagen, Denmark.

[24]WHO Collaborating Center for Food and Nutrition Policies, Department of Hygiene, Epidemiology and Medical Statistics, University of Athens Medical School, Athens, Greece.

[25]Hellenic Health Foundation, Athens, Greece.

[26]Department of Epidemiology, Harvard School of Public Health, Boston, MA, USA.

[27]Bureau of Epidemiologic Research, Academy of Athens, Athens, Greece.

[28]Cancer Registry and Histopathology Unit, "Civic- M.P.Arezzo" Hospital, ASP Ragusa, Italy.

[29]Epidemiology and Prevention Unit, Fondazione IRCCS Istituto Nazionale dei Tumori, Milan, Via Venezian, 1, 20133 Milan, Italy.

[30]Unit of Cancer Epidemiology, AO Citta' della Salute e della Scienza-University of Turin and Center for Cancer Prevention (CPO-Piemonte), Via Santena 7, 10126 Turin, Italy.

[31]Human Genetics Foundation (HuGeF), Via Nizza 52, 10126 Turin, Italy.

[32]Molecular and Nutritional Epidemiology Unit, Cancer Research and Prevention Institute – ISPO, Florence, Italy.

For peer review only

[33]the Department of Cancer Epidemiology, German Cancer Research Centre, Heidelberg, Germany.

[34]Department of Epidemiology, Julius Center for Health Sciences and Primary Care, University Medical Center, Utrecht, The Netherlands.

[35]Department of Statistics, Federal University of Rio Grande do Sul, Porto Alegre, Brazil.

[36]Department of Epidemiology & Biostatistics, School of Public Health, Imperial College London, London, United Kingdom.

[37]Department of Public Health, Section for Epidemiology, Aarhus University, Aarhus, Denmark.

[38]Department of Cardiology, Aalborg University Hospital, Aalborg, Denmark.

Corresponding Author:

Pietro Ferrari, PhD

Nutrition and Metabolism Section

International Agency for Research on Cancer

150, cours Albert Thomas, Lyon, France

Tel +33 472728031

Email: ferrarip@iarc.fr

Abbreviations:

ARC: alcohol related cancers

CVD: cardiovascular diseases

CHD: coronary heart disease

HR: Hazard ratio

CI: Confidence Intervals

EPIC: European Prospective Investigation into Cancer and nutrition

Keywords: lifetime alcohol, smoking status, overall mortality.

For peer review only

Abstract (300 words)

Objectives. To investigate the role of factors that modulate the association between alcohol and mortality, and to provide estimates of absolute risk of death.

Design. The European Prospective Investigation into Cancer and Nutrition (EPIC).

Setting. 23 centres in 10 countries.

Participants. 380,395 men and women, free of cancer, diabetes, heart attack or stroke at enrolment, followed up for 12.6 years on average.

Main outcome measures. 20,453 fatal events, of which 2,053 alcohol related cancers (ARC, including cancers of upper aero-digestive tract, liver, colorectal and female breast), 4,187 cardiovascular diseases (CVD/CHD), 856 violent deaths and injuries. Lifetime alcohol use was assessed at recruitment.

Results. Hazard ratios comparing extreme drinkers (≥30 g/day in women and ≥60 g/day in men) to moderate drinkers (0.1-4.9 g/day) were 1.27 (95%CI: 1.13,1.43) in women and 1.53 (1.39,1.68) in men. Strong associations were observed for ARC mortality, in men particularly, and ~~to~~ for violent deaths and injuries, in men only. No associations were observed for CVD/CHD mortality among drinkers, whereby hazard ratios were higher in never compared to moderate drinkers. Overall mortality seemed to be more strongly related to beer than wine use, particularly in men. The 10-year risks of overall death for women aged 60 years, drinking more than 30 g/day was 5% and 7%, for never and current smokers, respectively. Corresponding figures in men consuming more than 60 g/day were 11% and 18%, in never and current smokers respectively. In competing risks analyses, mortality due to CVD/CHD was more pronounced than ARC in men, while CVD/CHD and ARC mortality were of similar magnitude in women.

Conclusions. In this large European cohort, alcohol use was positively associated with overall mortality, ARC and violent death and injuries, but marginally to CVD/CHD. Absolute risks of death observed in EPIC suggest that alcohol is an important determinant of total mortality.

For peer review only

Article summary

Strengths and limitations of this study

- This study was based on information on dietary and lifestyle exposure collected in a large prospective investigation of European adults.
- Findings are based on 380,395 men and women (among whom 20,453 fatal events occurred) for which information on lifetime alcohol use was available, allowing separate consideration of former drinkers from ~~and~~ lifetime abstainers.
- Exclusion of study participants reporting a morbid condition at baseline, and sensitivity analyses excluding the first three years of follow-up limited the chance that reverse causality affected the findings.
- Although statistical models included many potentially relevant adjustment factors, residual confounding might partially account for the observed associations.
- Average lifetime alcohol consumption was evaluated in this study, whereas it is possible that specific drinking patterns in particular phases of life, as well as the effect of binge drinking or drinking during meals, may also be of particular relevance for mortality.

For peer review only

For peer review only

Introduction

Alcohol intake has been associated with an increased risk of death from a large list of morbid conditions, including digestive tract conditions, liver cirrhosis, chronic pancreatitis, hypertension, injuries and violence (1-3). In contrast, moderate alcohol drinking was suggested to be associated with a reduction in cardiovascular disease (CVD) mortality (4;5). As for cancer, the International Agency for Research on Cancer (IARC) and the World Cancer Research Fund/American Institute for Cancer Research (WCRF/AICR) concluded that alcohol use is associated with an increased risk to develop cancers of the upper aero-digestive tract, liver, colorectal and female breast (6;7). It has been estimated that alcohol accounted for about 2.7 million annual deaths and 3.8% of all deaths worldwide (8;9), but the impact of alcohol on mortality is differential with respect to specific causes of diseases (3).

Within the European Prospective Investigation into Cancer and Nutrition (EPIC), a recent work showed that heavy alcohol use was associated to a higher risk of death from alcohol-related cancer, external causes and ‘other causes’, while no associations were observed for coronary heart disease and other cardiovascular diseases (10).

In this work, we further investigated associations between alcohol use and overall and cause-specific mortality ~~were further investigated~~. Notably, potential variability of the relationships with respect to smoking habits, the type of alcoholic beverages, and country was explored. The cumulative probabilities of death were estimated for overall mortality and, in a competing risks framework, for specific mortality causes with respect to levels of alcohol, separately in men and women. Further, the burden of alcohol use in relation to a broad group of causes of deaths was evaluated by means of overall estimates of rate advancement periods, with respect to two alternative scenarios.

Methods

Study Population. EPIC is an on-going multi-centre study that has been described in detail previously (11). From 1992 to 2000, 521,448 individuals, aged 25 to 70 years were recruited in the surroundings of 23 centres in 10 European countries. Most of the participants were recruited from the general population residing in a given geographic area, a town, or a province. Exceptions were the cohorts of France (female members of a health insurance for school employees), Utrecht (breast cancer screening attendees), Ragusa (blood donors and their spouses), and Oxford (mainly vegetarian and healthy eaters). Some characteristics of the study population in the different participating countries are reported in Table 1. Study participants provided informed consent and completed questionnaires on their diet, lifestyle, and medical history. The study was approved by the relevant ethical review boards of each centre and the International Agency of Research on Cancer in Lyon, France (11).

Dietary and lifestyle assessment. Diet was assessed at enrolment using validated country- or center-specific dietary questionnaires (DQ) designed to capture habitual consumption over the preceding year. Lifetime alcohol consumption was assessed based on self-reported weekly consumption of wine, beer, and liquor at ages 20, 30, 40, 50 years in the lifestyle questionnaire. Information on lifetime alcohol consumption was available for approximately 76% of EPIC participants (12). Information on smoking status and duration, physical activity during leisure time, prevalent conditions at baseline, educational attainment, anthropometric measures and reproductive history was obtained using lifestyle questionnaires.

Assessment of causes of death. Vital status and information on cause and date of death were ascertained using record linkage with cancer registries, boards of health, and death registries (Denmark, Italy, The Netherlands, Spain, the United Kingdom), or by active follow-up (France Germany, Greece). Data were coded using the 10th revision of the International

For peer review only

Statistical Classification of Diseases, Injuries and Causes of Death (ICD-10) where the underlying cause is the official cause of death.

In this work, six different causes of deaths were selected: cardiovascular disease (CVD) [I00-I99 excluding I20-I25] and coronary heart disease (CHD) [I20-I25], alcohol related cancer (ARC), including colorectal cancer [C18- C20], female breast cancer [C50], upper aero-digestive cancers (UADT, including cancer of the mouth [C01 to C10 without C08=salivary gland], larynx [C21], pharynx [C11 to C14], oesophagus [C15]), violent deaths and injuries (Injury, poisoning and certain other consequences of external causes [S00-T98]; deaths due to respiratory diseases [J00-J99]; a group for all other causes (including external causes of morbidity and mortality [V01-Y98], unknown causes [R96-R99]).

Statistical analyses. Participants from Denmark (Aarhus, Copenhagen), France, Germany (Heidelberg, Potsdam), Greece, Italy (Florence, Varese, Ragusa, Turin), the Netherlands (Utrecht), Spain (Asturias, Granada, Murcia, Navarra, San Sebastian), and the United Kingdom (Cambridge, Oxford), were eligible for this analysis. We excluded the entire cohorts of Naples (Italy), Bilthoven (The Netherlands), Sweden and Norway because no information on past alcohol use was collected (n=118,082). Further exclusions concerned participants with incomplete vital status information (n=928), who had not filled out the dietary or lifestyle questionnaires (n=11,411), and participants whose ratio of energy intake to estimated energy requirement (n=7,592), calculated in terms of gender, body weight, height, and age, was in the top or bottom 1% in order to partially reduce the impact of outlier values (13). Participants that at recruitment reported a cancer (n=13,283), diabetes (n=11,240), myocardial infarction or heart disease (n=5,266) or stroke (n=3,246) were excluded from the analyses (n= 30,665 in total).

Cox proportional hazards models were used to compute mortality hazard ratios (HR), and 95% confidence intervals, for categories of average lifetime alcohol use; never drinkers, 1–4.9

For peer review only - http://bmjopen.bmj.com/site/about/guidelines.xhtml

g/day (reference category), 5–14.9, 15–29.9, 30–59.9, ≥60 g/day. In women, the last two alcohol categories were collapsed into a ≥30 g/day group. Time in the study up to death, loss or end of follow-up, whichever came first was the primary time variable. The Breslow method was adopted for handling ties. Models were stratified by center to control for differences in questionnaire design, follow-up procedures, and other center-specific effects (13). ~~Systematic adjustments were undertaken for age at recruitment, and other lifestyle factors~~ Systematic adjustments were undertaken for age at recruitment, BMI and height (continuous), an indicator for participants that quitted alcohol drinking, time since alcohol quitting (continuous), smoking (never, current with 1 to 15 cigarettes/day, current with more than 15 cigarettes/day, former smoker that quitted less than 10 years before recruitment, former smoker that quitted more than 10 years before recruitment, current smoker of other than cigarettes, unknown [n=8,819]), duration of smoking (continuous), age at start smoking (less than 19 years, more than 19 years, unknown [n=39,041]), educational attainment (five categories of level of schooling: none, primary, technical, or degree or more, unknown [n=14,223]) as a proxy variable for socioeconomic status, physical activity (inactive, moderately inactive, moderately active, active, unknown [n=328]), and energy intake (continuous). In women the models were further adjusted for menopausal status (dichotomized as natural postmenopausal or surgical versus pre- or peri-menopausal, as assessed at baseline), ever use of replacement hormones, and number of full term pregnancies (nulliparous, 1 or 2 children, more than three, unknown [n=6,482]). Indicator variables specific to some of the confounding factors were used to model missing values, after checking that the parameters associated with these indicators were not statistically significantly associated with risk of death.

Models for overall and cause-specific mortality were fitted, separately for men and women. An overall test of significance of HRs related to alcohol use was determined by computing p-

For peer review only

values (p_{Wald}) for Wald test statistics compared to a chi-square distribution with degrees of freedom equal to the number of alcohol categories minus one. The proportional hazards assumption in the Cox model was satisfied and evaluated via inclusion into the disease model of interaction terms between lifetime alcohol and follow-up time. To reduce the chance of reverse causality, sensitivity analyses were run excluding the first three years of follow-up. As results were not different from those using the entire cohort, they were not shown. Analyses excluding former drinkers (4% and 5% of the study populations, in men and women respectively) provided very similar results (results not shown).

Evaluating heterogeneity. Effect modification in the relations between alcohol and mortality by, in turn, smoking status (never, ever), smoking status (never and current smokers), and recruitment country was assessed. Models with main effects and interaction terms were fitted and compared to models with main effects only. The difference in log-likelihood (likelihood ratio test statistics) was compared to a chi-square distribution with degrees of freedom equal to the number of interaction terms. HRs for alcohol categories across levels of interacting variables were computed as linear combinations of main effects and interactions. Associations with wine and beer uses (each grouped as never, 0.1-2.9 g/day (reference), 3-9.9, 10-19.9, 20-39.9, ≥40 g/day, ≥20 g/day in women) and total mortality were assessed in mutually adjusted models. The difference of association for wine and beer use in relation to overall mortality was assessed by inspecting the significance of the parameter related to the arithmetic difference of wine and beer use (expressed on the log-scale plus 1 to deal with abstainers) in a model that also included their arithmetic sum. When assessing the association for wine (beer) intake, analyses were restricted to moderate lifetime drinkers of beer (wine) and spirits (below 3 g/day).

Flexible parametric survival models (14) with age as the time-scale were used to evaluate whether the association between alcohol intake and mortality rate varied by attained age. The

For peer review only

origin of the time-scale was set to 30 years as the hazard of death is essentially zero prior to that age. The baseline cumulative hazard was modeled using restricted cubic splines with three internal knots placed at evenly spaced centiles of the uncensored log-survival times in order to ensure that an equivalent number of deaths occurred between each knot (15). Interactions between alcohol intake and the time scale were modeled using restricted cubic splines with one knot placed at the median of uncensored log-survival times. HRs and differences in survival functions were calculated from the fitted models and plotted against attained age, along with confidence intervals calculated based on delta method variance estimates.

Possible departures from linearity in the association between average lifetime alcohol use and total mortality were assessed using fractional polynomials (16), a subset of generalized linear models in which various powers (−2, −1, −0.5, 0, 0.5, 1, 2, 3) of the covariate(s) of interest are entered into the linear predictor. Fractional polynomials of order two were consistently used in this work for lifetime alcohol use (17). Non-linearity was tested comparing the difference in log-likelihood of a model with the fractional polynomials with a model with a linear term only to a chi-square distribution with three degrees of freedom (16).

Absolute risks. An extension of the Cox proportional hazards model was employed to fit cause- specific associations between lifetime alcohol use and cause-specific mortality in a competing risks framework (18). An augmented dataset was created where the initial dataset is replicated a number of times equal to the different causes of death. In each replicated dataset, competing causes of death were set to censored observations, and the analyses were stratified by the event type (19). The relationship between each confounder variables and cause-specific mortality was assumed homogenous across causes of death. In this way competing risks was accounted for, and cumulative cause-specific and overall mortality curves were estimated for heavy (greater than 30 and 60 g/day, in women and men

For peer review only

respectively) and moderate (0.1 to 4.9 g/day) drinkers, separately for never and current smokers (20). Cumulative mortality curves were obtained for participants aged 60 years, using mean values for continuous confounding factors, and average frequencies for categorical confounders.

Quantifying the alcohol burden. The burden of alcohol on mortality was quantified with estimates of the rate advancement period (RAP) (21), according to two scenarios with threshold levels equal to 5 and 15 g/day. For overall and cause-specific risk of death, RAP were computed, dividing the log(HR) estimate comparing alcohol users above and the threshold with alcohol drinkers between 0.1 g/day and the threshold, by the log of the parameter associated with age. Never alcohol users were not included in the estimation. Associated 95% confidence intervals were also determined. RAP estimates express the impact of a given exposure on the risk of death, by determining the time (in years) by which the risk of death is anticipated for exposed study participants compared to non-exposed.

Statistical tests were two-sided, and p-values <0.05 were considered statistically significant. All analyses were performed using SAS 9.2 (22) and Stata 12.1 (23).

Results

Baseline characteristics. The current analysis was based on 247,795 female and 101,935 male study participants. The median age at enrolment was 52 years for women and 53 years for men. Study participants were foll–owed on average 12.6 years, accumulating 4,800,585 person-years, during which a total of 20,453 fatal events were recorded (Table 1).

Drinking patterns differed substantially between men and women (Table 2). In women, 10% (n=25,146) of participants were lifetime never drinkers, while 45% (n=112,281) and 2% (n=6,042) were moderate (0.1 to 4.9 g/day) and heavy users (>30g/day), respectively. Conversely, only 1.5% (n=1,600) of men reported having never consumed alcohol, 14%

For peer review only

(n=14,287) were moderate drinkers, whilst 29% (n=29,124) were heavy or extreme drinkers (30 to 59.9 and ≥60 g/day). Furthermore, the vast majority of women who were regular drinkers (total alcohol intake ≥10g/day) drank predominantly wine (91%), rather than beer (9%), whilst regular drinkers in men drank beer (46%) and wine (54%) in similar proportion.

Compared to never and moderate drinkers, women with higher alcohol use had higher levels of education and physical activity, and were more likely to be current smokers, or pre-/peri-menopausal. Never alcohol users were less likely to have used hormonal replacement therapy than alcohol drinkers. In men, the trends were somewhat less apparent. Heavy and extreme alcohol users (≥30 g/day) were more often current smokers, attained lower educational level, and had higher energy intake levels, compared to moderate drinkers. Never drinkers were physically less active than alcohol drinkers.

Lifetime alcohol and total mortality. Lifetime average alcohol use was strongly associated with total mortality, in that both never and heavy drinkers (≥30 g/day) had notably higher mortality rates than did light to moderate drinkers (0.1-4.9 g/day), a pattern that was consistently apparent among female and male study participants (Figure 1). The HR comparing never and heavy drinkers to moderate drinkers in women was 1.26 (95% CI: 1.18-1.35) and 1.27 (1.13-1.43), respectively. The corresponding HRs among men were 1.29 (1.10-1.51) for never drinkers, 1.15 (1.06-1.24) for heavy drinkers and 1.53 (1.39-1.68) for extreme drinkers (≥60 g/day).

Lifetime alcohol and cause specific mortality. In men, extreme alcohol use was associated with mortality due to alcohol related cancers ($HR_{\geq 60 \text{ vs ref}}$ = 2.62: 1.90, 3.62), other cancers (1.34: 1.13, 1.59), violent deaths and injuries (1.93: 1.27, 2.91), and other causes (1.98: 1.67, 2.34). With the exception of the category for never drinkers, alcohol intake was not associated with CVD or CHD mortality, both in women and men. Among women, heavy drinkers displayed $HR_{\geq 30 \text{ vs ref}}$ equal to 1.49 (1.07, 2.06) for alcohol related cancers. Respiratory

For peer review only

diseases were not associated with lifetime alcohol in women, while results were suggestive of an increased risk in extreme alcohol users compared to moderate users in men (Supplementary Figure 1). Dose–response relationships evaluated with fractional polynomials are displayed in Supplementary Figures 2 and 3, for women and men respectively.

Evaluating heterogeneity. In both sexes, alcohol related HRs for overall mortality were of similar magnitude in never and current smokers (Table 3). Analyses conducted by smoking intensity (never vs. heavy smokers, i.e. more than 15 cigarettes/day) produced very similar evidence (results not shown). Cause-specific analyses showed mostly homogeneous alcohol-related HRs by smoking status (results not shown). In women, beer use was more strongly related than wine to overall mortality for amounts greater than 3g/day compared to the reference category (0.1 to 2.9 g/day). Lifetime never wine and beer users displayed higher risks than moderate drinkers. The associations between lifetime alcohol and overall risk of mortality were differential across country of origin in men ($p_{heterogeneity}$=0.012) but not in women ($p_{heterogeneity}$=0.511), as reported in Supplementary Figures 4 and 5, with stronger relationships observed in Northern European countries compared to Southern European countries.

The $HR_{\geq 60 \text{ vs ref}}$ for men was more pronounced at earlier ages, and were close to ~~approached~~ one as attained age approached 90 years ($p_{likelihood\text{-}ratio}$ for age-varying versus age invariant parameterization=0.003), however extreme male drinkers exhibited lower cumulative survival probability than the reference group throughout the lifespan (Supplementary Figure 6). No such age varying association was apparent for women ($p_{likelihood\text{-}ratio}$=0.80).

Absolute risks. The 10-year risk of death at the age of 60 years for heavy drinkers was 5% and 7% in women (≥30 g/day), and 11% and 18% in men (≥60 g/day), for never and current smokers, respectively (Figure 2). Corresponding figures in moderate drinkers (0.1 to 4.9 g/day) were 3% and 4% in women, and 5% and 8% in men. Based on a competing risks

For peer review only

analysis, it was estimated that, at the age of 60 years, a female lifetime heavy alcohol drinker and smoker had a 10-years risk of death of 1.0% for ARC, 1.2% for CVD/CHD, and 0.2% for violent death and injuries, as displayed in Figure 3. Corresponding figures for males (≥60 g/day) were 2.2% (ARC), 5.0% (CVD/CHD) and 1% (violent death and injuries). Risks for moderate drinkers for ARC, CVD/CHD and violent death and injuries were 0.8%, 1.2%, 0.2% and 0.9%, 4.0%, 0.3%, in women and men, respectively. Consistently lower risks were observed for never smoker individuals, with estimates equal to 0.5%, 0.7% and 0.1% in women, and 1%, 2.1% and 0.3% in men.

Rate advancement period. The impact of lifetime alcohol on overall and cause-specific mortality was estimated with RAP values (Table 4). In women, RAP for overall mortality were equal to 0.36 years (95%CI: -0.05, 0.76) and 0.83 (0.26, 1.39), for the 5 and 15 g/day scenario, respectively. In men, RAP values were equal to 0.15 (-0.48, 0.76) and 1.42 (0.96, 1.89), for 5 and 15 g/day respectively. RAP values were sizeable for mortality due to alcohol related cancer (5.03: 3.07, 7.00) and violent death and injuries (11.83: 3.92, 18.17) in the second scenario.

For peer review only

Discussion

In this large European prospective study, the association between alcohol use and overall and cause-specific risk of death was evaluated in eight European populations. When accounting for potential confounding factors, average lifetime alcohol use was strongly associated with overall mortality, whereas lifetime never alcohol users consistently displayed a higher risk of death compared to moderate drinkers. These results are in agreement with a recent evaluation of alcohol and cause-specific mortality in EPIC (10). With respect to this recent study (10), further analyses were conducted to deeply investigate the role of factors that modulate the

association between alcohol use and the risk of death, notably smoking and types of alcohol beverage. Estimates of 10-year risk of death in relation to levels of alcohol use were provided.

This study has several strengths. It was conducted using a large prospective cohort, where dietary and lifestyle exposure information were collected on disease-free individuals. Information on lifetime alcohol use was available on 76% of the cohort, allowing separate consideration of former drinkers and lifetime abstainers. Further, exclusion of study participants reporting a morbid condition at baseline, and sensitivity analyses excluding the first three years of follow-up suggest that reverse causality is unlikely to have affected the results. One potential weakness of this study is that, although statistical models included many potentially relevant adjustment factors, residual confounding might partially account for the observed associations. In addition, average lifetime alcohol consumption was used throughout this study, whereby it is possible that specific drinking patterns in particular phases of life (10), as well as the effect of binge drinking or drinking during meals may be of particular relevance for mortality.

A recent Russian study found ~~that~~ a strong relationship between vodka and risk of death (24). While an apparent J-shaped relationship between alcohol use and mortality has been reported (25;26), the interpretation of this pattern is the subject of some controversy. It has been suggested that alcohol abstinence does not truly entail greater risk of death than moderate use, and that misclassification of alcohol quantity and lack of accuracy in reporting prevalent morbid conditions at baseline in the group of never drinkers (27) could explain the excess risks observed. This reasoning motivated our choice of considering moderate alcohol drinkers, as the reference category throughout this work. Moreover, residual and unmeasured confounding are plausible drivers of the association (28). These suggestions are supported by our findings that never drinkers are at increased risk of death due to violence and injury. This

For peer review only

implausible association casts considerable doubt on the veracity of the apparent increased risk of death among never drinkers.

The overall mortality hazard ratio for men with extreme versus moderate alcohol use was greater at younger ~~earlier~~ ages, and approached one ~~unity~~ as age ~~advances~~ increased towards 90 years. This result reflects the comparatively low incidence of death through middle age. Consideration of the absolute risk of death, however, suggests that moderate drinkers have a substantial cumulative survival advantage over extreme drinkers throughout the adult lifespan. It has been suggested that wine drinking could be more favourably associated than other alcoholic beverages to the risk of CHD and some cancers (29-32). In this study beer use displayed more apparent risk patterns than wine ~~intake~~consumption, particularly in men. Although we believe that this finding is relevant, we call for cautious interpretations of these results, as the lifestyle profile of wine and beer drinkers is profoundly different. The associations between alcohol and mortality were heterogeneous across countries in men, but not in women. This could be due to the larger amount of alcohol consumed in men than in women, naturally increasing the variability of exposure and the statistical power to detect associations, and of the larger variability characterizing drinking habits in men, such as binge drinking, drinking during meals and other societal aspects. Although no heterogeneity was observed by smoking status, ~~at all levels of alcohol intake the~~ mortality among smokers was higher than the mortality among non-smokers , so the absolute increase in risk associated with alcohol intake is more extreme among smokers. This differential increase in cumulative probability of death emphasises the central role of tobacco as a risk factor for mortality, as well as the potential extra harm of increased alcohol consumption.

In the EPIC study, although associations with alcohol were mostly apparent for ARC and violent death and injuries, absolute risks were highest for CVD/CHD in men, while CVD/CHD and ARC risks were of similar magnitude in women. In general, as individuals

For peer review only

reporting a prevalent condition at recruitment (either cancer, diabetes, heart attack or stroke) were excluded from the analysis in an effort to minimize reverse causality, our estimates of absolute risks of death are possibly underestimated. RAP values were estimated to appreciate the risk benefit of alcohol drinkers if they were to modify their exposure, according to a counterfactual scenario (21;33). Consistently in men and women, RAP values for overall mortality were larger when the reference category was set to 0.1 to 15 g/day than when using a threshold of 5 g/day, thus indicating that, based on the EPIC study, the benefit for drinkers could be largest if they reduced their intake is reduced to levels below 15 g/day. These results are in line with findings of a recent work in the UK population, where the reduction of overall mortality was estimated to be optimized for alcohol reduction up to a median population level of 5 g/day (34).

In a large prospective study in Europe, lifetime alcohol intake was significantly associated with both overall and ARC specific mortality. In men, positive associations were observed for violent deaths and injuries, while CVD and CHD deaths were not associated with alcohol use among drinkers. Our findings suggest that these associations do not differ between never and current smokers, and were stronger for beer than for wine usedrinkers.

For peer review only

Funding statement

This work was supported by ~~la~~ the Direction Générale de la Santé (French Ministry of Health)~~, with contract~~ (Grant GR-IARC-2003-09-12-01), by the European Commission (Directorate General for Health and Consumer Affairs) and the International Agency for Research on Cancer. The national cohorts are supported by the Danish Cancer Society (Denmark); the Ligue Contre le Cancer, the Institut Gustave Roussy, the Mutuelle Générale de l'Education Nationale, and the Institut National de la Santé et de la Recherche Médicale (France); the Deutsche Krebshilfe, the Deutsches Krebsforschungszentrum, and the Federal Ministry of Education and Research (Germany); the Hellenic Health Foundation, the Stavros Niarchos Foundation, and the Hellenic Ministry of Health and Social Solidarity (Greece); the Italian Association for Research on Cancer and the National Research Council (Italy); the Dutch Ministry of Public Health, Welfare and Sports, the Netherlands Cancer Registry, LK Research Funds, Dutch Prevention Funds, the Dutch Zorg Onderzoek Nederland, the World Cancer Research Fund, and Statistics Netherlands (Netherlands); European Research Council-2009-AdG 232997 and the Nordforsk, Nordic Centre of Excellence program on Food, Nutrition and Health (Norway); the Health Research Fund, Regional Governments of Andalucýa, Asturias, Basque Country, Murcia (project 6236) and Navarra, Instituto de Salud Carlos III, Redes de Investigacion Cooperativa (RD06/0020) (Spain); the Swedish Cancer Society, the Swedish Scientific Council, and the Regional Government of Skåne (Sweden); Cancer Research UK, the Medical Research Council, the Stroke Association, the British Heart Foundation, the Department of Health, the Food Standards Agency, and the Wellcome Trust (United Kingdom). The work undertaken by David C Muller was done during the tenure of an IARC, Australia postdoctoral fellowship, supported by the Cancer Council Australia.

Contributorship statement

Pietro FERRARI, Idlir LICAJ, Isabelle ROMIEU, Paul BRENNAN, Heiner BOEING conceptualized the study and defined the analytical strategy. Pietro FERRARI and Idlir LICAJ had full access to all the data in the study and take responsibility for the integrity of the data and the accuracy of the data analysis. Pietro FERRARI, Idlir LICAJ, David C MULLER, Per Kragh ANDERSEN, Luciana NUNES performed statistical analyses and provided preliminary interpretation of findings. Pietro FERRARI, David C MULLER, Mattias JOHANSSON, Idlir LICAJ, Paul BRENNAN contributed to draft the manuscript. Elisabete WEIDERPASS, Laure DOSSUS, Laureen DARTOIS, Guy FAGHERAZZI,

For peer review only

Kathryn Erica BRADBURY, Kay-Tee KHAW, Eric Jeffrey DUELL, Aurelio BARRICARTE, Esther MOLINA-MONTES, Carmen NAVARRO SANCHEZ, Larraitz ARRIOLA, Peter WALLSTRÖM, Anne TJØNNELAND, Anja OLSEN, Antonia TRICHOPOULOU, Vasiliki BENETOU, Dimitrios TRICHOPOULOS, Rosario TUMINO, Claudia AGNOLI, Carlotta SACERDOTE, Domenico PALLI, Kuanrong LI, Rudolf KAAKS, Petra PEETERS, Joline Wilhelma Johanna BEULENE, Marc GUNTER, Teresa NORAT, Kim OVERVAD, Elio RIBOLI, Paul BRENNAN, and Isabelle ROMIEU played a key role in the acquisition of the data, and were active in searching for funding to continue the study. With respect to this work, they all critically helped in the interpretation of results, revised the manuscript and provided relevant intellectual input.

Competing Interest Declaration

All authors have completed the Unified Competing Interest form at www.icmje.org/coi_disclosure.pdf (available on request from the corresponding author). Dr. Kathryn E BRADBURY reports grants from Cancer Research UK, during the conduct of the study. The rest of the authors declared no support from any organisation for the submitted work, no financial relationships with any organisations that might have an interest in the submitted work in the previous three years, no other relationships or activities that could appear to have influenced the submitted work.

Sharing statement

Statistical code is available from the corresponding author by emailing ferrarip@iarc.fr.

For peer review only

Reference List

(1) Thun MJ, Peto R, Lopez AD, Monaco JH, Henley SJ, Heath CW, Jr., et al. Alcohol consumption and mortality among middle-aged and elderly U.S. adults. N Engl J Med 1997 Dec 11;337(24):1705-14.

(2) Bagnardi V, Blangiardo M, La Vecchia C., Corrao G. A meta-analysis of alcohol drinking and cancer risk. Br J Cancer 2001 Nov 30;85(11):1700-5.

(3) Rehm J, Baliunas D, Borges GL, Graham K, Irving H, Kehoe T, et al. The relation between different dimensions of alcohol consumption and burden of disease: an overview. Addiction 2010 May;105(5):817-43.

(4) Corrao G, Bagnardi V, Zambon A, La VC. A meta-analysis of alcohol consumption and the risk of 15 diseases. Prev Med 2004 May;38(5):613-9.

(5) Ronksley PE, Brien SE, Turner BJ, Mukamal KJ, Ghali WA. Association of alcohol consumption with selected cardiovascular disease outcomes: a systematic review and meta-analysis. BMJ 2011;342:d671.

(6) IARC. Alcohol consumption and ethyl carbamate. 96 ed. IARC; 2010.

(7) WCRF/AICR. Food, nutrition, physical activity and the prevention of cancer: a global perspective. Washington DC: AICR; 2007.

(8) Rehm J, Mathers C, Popova S, Thavorncharoensap M, Teerawattananon Y, Patra J. Global burden of disease and injury and economic cost attributable to alcohol use and alcohol-use disorders. Lancet 2009 Jun 27;373(9682):2223-33.

(9) Lim SS, Vos T, Flaxman AD, Danaei G, Shibuya K, Adair-Rohani H, et al. A comparative risk assessment of burden of disease and injury attributable to 67 risk factors and risk factor clusters in 21 regions, 1990-2010: a systematic analysis for the Global Burden of Disease Study 2010. Lancet 2012 Dec 15;380(9859):2224-60.

(10) Bergmann MM, Rehm J, Klipstein-Grobusch K, Boeing H, Schutze M, Drogan D, et al. The association of pattern of lifetime alcohol use and cause of death in the European Prospective Investigation into Cancer and Nutrition (EPIC) study. Int J Epidemiol 2013 Dec;42(6):1772-90.

(11) Riboli E, Hunt KJ, Slimani N, Ferrari P, Norat T, Fahey M, et al. European Prospective Investigation into Cancer and Nutrition (EPIC): study populations and data collection. Public Health Nutr 2002 Dec;5(6B):1113-24.

(12) Ferrari P, Jenab M, Norat T, Moskal A, Slimani N, Olsen A, et al. Lifetime and baseline alcohol intake and risk of colon and rectal cancers in the European prospective investigation into cancer and nutrition (EPIC). Int J Cancer 2007 Nov 1;121(9):2065-72.

(13) Ferrari P, Day NE, Boshuizen HC, Roddam A, Hoffmann K, Thiebaut A, et al. The evaluation of the diet/disease relation in the EPIC study: considerations for the calibration and the disease models. Int J Epidemiol 2008 Apr;37(2):368-78.

For peer review only

(14) Royston P, Parmar MK. The use of restricted mean survival time to estimate the treatment effect in randomized clinical trials when the proportional hazards assumption is in doubt. Stat Med 2011 Aug 30;30(19):2409-21.

(15) Heinzl H, Kaider A. Gaining more flexibility in Cox proportional hazards regression models with cubic spline functions. Comput Methods Programs Biomed 1997 Nov;54(3):201-8.

(16) Sauerbrei W, Royston P. Building multivariable prognostic and diagnostic models: Transformation of the predictors by using fractional polynomials. Journal ofthe Royal Statistical Society, Series A 1999;62:71-94.

(17) Royston P, Ambler G, Sauerbrei W. The use of fractional polynomials to model continuous risk variables in epidemiology. Int J Epidemiol 1999 Oct;28(5):964-74.

(18) Andersen PK, Geskus RB, de WT, Putter H. Competing risks in epidemiology: possibilities and pitfalls. Int J Epidemiol 2012 Jun;41(3):861-70.

(19) Putter H, Fiocco M, Geskus RB. Tutorial in biostatistics: competing risks and multi-state models. Stat Med 2007 May 20;26(11):2389-430.

(20) Rosthoj S, Andersen PK, Abildstrom SZ. SAS macros for estimation of the cumulative incidence functions based on a Cox regression model for competing risks survival data. Comput Methods Programs Biomed 2004 Apr;74(1):69-75.

(21) Brenner H, Gefeller O, Greenland S. Risk and rate advancement periods as measures of exposure impact on the occurrence of chronic diseases. Epidemiology 1993 May;4(3):229-36.

(22) SAS Institute Inc. 2002. NC, USA, Cary.

(23) Stata Statistical Software: *Release 12*. Version StataCorp. College Station, TX: StataCorp LP.: 2011.

(24) Zaridze D, Lewington S, Boroda A, Scelo G, Karpov R, Lazarev A, et al. Alcohol and mortality in Russia: prospective observational study of 151 000 adults. Lancet 2014 Jan 30;(13):10-6736.

(25) Thun MJ, Peto R, Lopez AD, Monaco JH, Henley SJ, Heath CW, Jr., et al. Alcohol consumption and mortality among middle-aged and elderly U.S. adults. N Engl J Med 1997 Dec 11;337(24):1705-14.

(26) Lin Y, Kikuchi S, Tamakoshi A, Wakai K, Kawamura T, Iso H, et al. Alcohol consumption and mortality among middle-aged and elderly Japanese men and women. Ann Epidemiol 2005 Sep;15(8):590-7.

(27) Fillmore KM, Stockwell T, Chikritzhs T, Bostrom A, Kerr W. Moderate alcohol use and reduced mortality risk: systematic error in prospective studies and new hypotheses. Ann Epidemiol 2007 May;17(5 Suppl):S16-S23.

For peer review only

(28) Naimi TS, Brown DW, Brewer RD, Giles WH, Mensah G, Serdula MK, et al. Cardiovascular risk factors and confounders among nondrinking and moderate-drinking U.S. adults. Am J Prev Med 2005 May;28(4):369-73.

(29) Klatsky AL, Friedman GD, Armstrong MA, Kipp H. Wine, liquor, beer, and mortality. Am J Epidemiol 2003 Sep 15;158(6):585-95.

(30) Maskarinec G, Meng L, Kolonel LN. Alcohol intake, body weight, and mortality in a multiethnic prospective cohort. Epidemiology 1998 Nov;9(6):654-61.

(31) Gronbaek M, Becker U, Johansen D, Gottschau A, Schnohr P, Hein HO, et al. Type of alcohol consumed and mortality from all causes, coronary heart disease, and cancer. Ann Intern Med 2000 Sep;19;133(6):411-9.

(32) Renaud SC, Gueguen R, Siest G, Salamon R. Wine, beer, and mortality in middle-aged men from eastern France. Arch Intern Med 1999 Sep 13;159(16):1865-70.

(33) Gellert C, Schottker B, Holleczek B, Stegmaier C, Muller H, Brenner H. Using rate advancement periods for communicating the benefits of quitting smoking to older smokers. Tob Control 2013 Jul;22(4):227-30.

(34) Nichols M, Scarborough P, Allender S, Rayner M. What is the optimal level of population alcohol consumption for chronic disease prevention in England? Modelling the impact of changes in average consumption levels. BMJ Open 2012 May 30;2(3):e000957.

For peer review only

Table 1. Country and sex-specific number of participants (N), person-years (PY), cause-specific and overall number of deaths.

					Cancers									
Country	N	PY	CHD[1]	CVD[2]	Breast	UADT[3]	Liver	Colon-rectum	Total[4]	Other cancers[5]	Violent & Injuries[6]	Resp[7]	Other causes[8]	Total
Women														
France	65,127	971,127	45	202	62	8	4	27	101	678	115	73	1,619	2,833
Italy	24,956	306,244	26	87	71	6	12	53	142	293	30	12	120	710
Spain	23,616	323,027	41	50	51	6	9	47	113	243	46	9	119	621
UK	50,251	651,640	387	320	160	30	15	127	332	716	85	161	1,091	3,092
Netherlands	14,583	189,531	110	137	58	14	7	78	157	370	20	65	209	1,068
Greece	14,391	143,150	139	100	41	2	10	18	71	146	26	27	94	603
Germany	27,098	307,380	56	74	64	12	14	44	134	256	35	25	114	694
Denmark	27,773	328,375	84	143	128	23	17	110	278	648	51	116	548	1,868
All	247,795	3,220,474	888	1,113	635	101	88	504	1,328	3,335	408	488	3,848	11,489
Men														
France	-	-	-	-	-	-	-	-	-	-	-	-	-	-
Italy	13,471	168,992	53	59	-	8	10	39	57	240	35	13	131	588
Spain	14,089	189,942	136	88	-	34	14	62	110	351	81	42	151	959
UK	20,452	262,720	438	229	-	41	11	68	120	567	88	171	1,040	2,653
Netherlands	-	-	-	-	-	-	-	-	-	-	-	-	-	-
Greece	9,726	90,989	193	141	-	12	20	31	63	279	49	54	99	878
Germany	19,743	221,724	167	138	-	37	25	65	127	437	84	35	264	1,253
Denmark	24,454	282,622	273	271	-	91	31	126	248	838	111	95	794	2,633
All	101,935	1,216,988	1,260	926	-	223	111	391	725	2,712	448	410	2,479	8,964

[1]CHD: Coronary heart disease [I20-I25] deaths; [2]CVD: Cardiovascular disease [I00-I99 except I20-I25] deaths; [3]UADT: deaths from upper aero-digestive cancers (including cancer of the mouth [C01 to C10 without C08=salivary gland]), larynx [C21], pharynx [C11 to C14], oesophagus [C15]); [4]Total frequency of alcohol related cancers; [5]Other cancers: deaths from all other cancers; [6]Violent deaths and injuries, including injury, poisoning and certain other consequences of external causes [S00-T98], and external causes of morbidity and mortality [V01-Y98]; [7]Resp=respiratory diseases [J00-J99].

For peer review only

Table 2. Characteristics of the study population at recruitment, according to amount and type of alcohol intake (g/day) in the EPIC study[1].

Characteristics	Unit	Never drinkers	Lifetime drinkers							
			0.1-4.9	5-14.9	15-29.9	30-59.9[2]	>60[2]	Total[3]	Wine consumers[4]	Beer consumers[4]
Women										
Number of participants	n	25,146	112,281	77,147	27,179	6,042	-	247,795	85,965	8,748
Person-Years	-	330,854	1,460,315	998,547	352,220	78,538	-	3,220,474	1,124,546	110,761
Age at recruitment	Years	52 (9)	52 (10)	51 (10)	49 (11)	47 (11)	-	51 (38-63)	52 (9)	46 (12)
Lifetime alcohol intake	g/day	0 (-)	2 (2)	9 (3)	20 (4)	43 (21)	-	7 (0-17)	12 (9)	11 (9)
Educational attainment[5]	%	14	22	27	33	37	-	25	28	28
Current smokers	%	13	14	18	24	31	-	17	17	28
Body mass index	kg/m^2	27 (5)	25 (5)	25 (4)	24 (4)	24 (4)	-	25 (20-31)	24 (4)	25 (4)
Height	cm	158 (6)	161 (6)	162 (6)	163 (7)	164 (6)	-	162 (153-170)	162 (6)	163 (7)
(Moderately) Active	%	26	39	44	44	43	-	40	42	42
Ever use of HRT[6]	%	16	25	29	28	25	-	25	50	34
Post-menopausal status[7]	%	50	49	49	44	38	-	48	29	20
Energy intake	kcal/day	1,848 (537)	1,943 (537)	2,015 (536)	2,090 (552)	2,195 (602)	-	1978 (542)	2,046 (544)	1,976 (545)
Men										
Number of participants	n	1,600	14,287	28,875	28,049	20,788	8,336	101,935	26,137	22,136
Person-Years	-	19,114	171,739	345,899	333,784	247,612	98,841	1,216,989	317,937	259,934
Age at recruitment	Years	53 (11)	53 (11)	53 (9)	52 (9)	52 (9)	52 (9)	53 (41-64)	53 (9)	52 (10)
Lifetime alcohol intake	g/day	0 (-)	2 (2)	10 (3)	22 (4)	42 (8)	94 (45)	25 (3-45)	30 (27)	22 (25)
Educational attainment[5]	%	21	30	31	32	26	14	29	22	27
Current smokers	%	28	22	25	30	36	49	30	31	33
Body mass index	kg/m^2	27 (4)	26 (4)	26 (3)	27 (3)	27 (4)	28 (4)	27 (22-31)	27 (4)	27 (4)
Height	cm	171 (7)	174 (7)	175 (7)	175 (7)	174 (7)	172 (7)	174 (165-183)	172 (7)	175 (7)
(Moderately) Active	%	42	46	50	52	52	50	50	48	52
Energy intake	kcal/day	2,284 (675)	2,267 (650)	2,315 (618)	2,417 (622)	2,569 (646)	2,789 (716)	2,427 (656)	2,487 (652)	2,369 (651)

[1]Means ± SDs are presented for continuous variables, frequencies for categorical variables; [2]In women the last alcohol category is ≥30g/day; [3]For continuous variables (with exception of energy intake), mean (10th-90th percentile) values are reported; [4]Study subjects consuming more than 10 g/day of wine (or beer), and consuming less than 3 g/day of beer (or wine); [5]Subjects with a university degree or more; [6]HRT=hormonal replacement therapy; [7]Post-menopausal women plus women who underwent an ovariectomy.

For peer review only

Figure 1. Number of deaths, person-years (PY) and multivariable hazard ratios[1] (HR) with 95% confidence intervals (CI) and p-value of the Wald test for statistical significance for overall and cause-specific mortality by categories of lifetime alcohol use, in women and men.

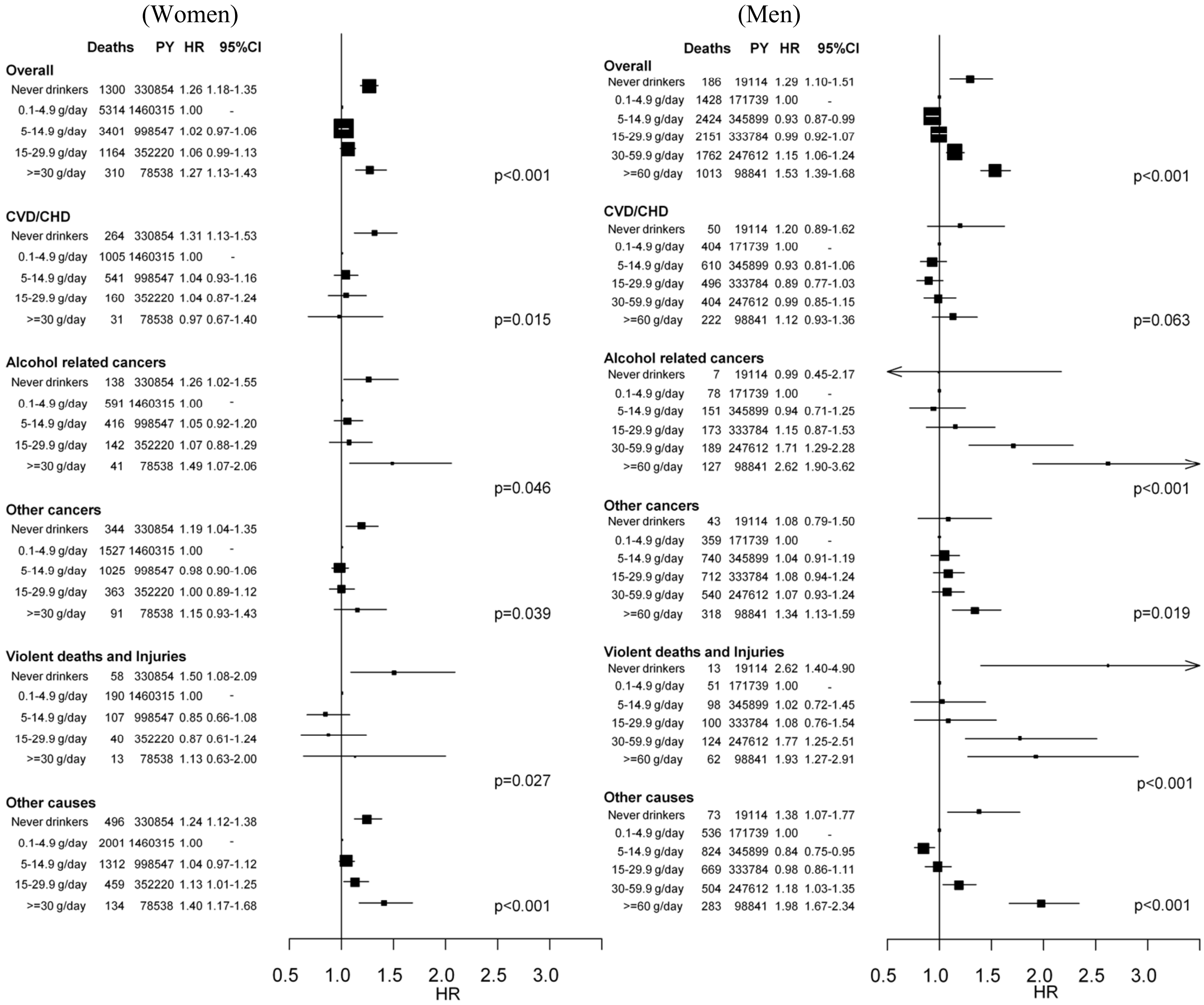

[1]Models were stratified by center. Systematic adjustment was undertaken for age at recruitment, BMI and height, former drinking, time since alcohol quitting, smoking status, duration of smoking, age at start smoking, educational attainment, and energy intake. In women adjustment was undertaken for menopausal status, ever use of replacement hormones, and number of full term pregnancies.

For peer review only - http://bmjopen.bmj.com/site/about/guidelines.xhtml

Table 3. Sex-specific number of deaths, hazard ratios (HR)[1] and 95%CI for overall mortality by categories of lifetime alcohol use (g/day), by smoking status (never and current smokers), and type of alcoholic beverage.

Women

	Never smokers			Current smokers			
	deaths	HR[2]	(95% CI)	deaths	HR[2]	(95% CI)	$p_{heterog}$[3]
**Overall**							
Never	1,009	1.34	(1.24, 1.45)	154	1.72	(1.32, 2.23)	
0.1-4.9	3,046	1	Ref	1,021	1.53	(1.23, 1.90)	
5-14.9	1,550	1.04	(0.98, 1.11)	874	1.51	(1.21, 1.88)	
15-29.9	397	1.04	(0.94, 1.16)	435	1.74	(1.38, 2.19)	
≥ 30	82	1.29	(1.03, 1.61)	140	2.08	(1.59, 2.73)	
p_{Wald}[4]			<0.001			<0.001	0.150

	Wine use			Beer use			
	deaths	HR[5]	(95% CI)	deaths	HR[5]	(95% CI)	$p_{difference}$[6]
**Overall**							
Never	2,156	1.15	(1.09, 1.22)	5,041	1.06	(1.02, 1.12)	
0.1-2.9	5,109	1	Ref	5,477	1	Ref	
3-9.9	2,813	0.96	(0.92, 1.01)	787	1.15	(1.07, 1.24)	
10-19.9	1,057	1.00	(0.93, 1.07)	147	1.50	(1.27, 1.77)	
≥ 20	354	1.14	(1.02, 1.27)	37	1.47	(1.06, 2.04)	
p_{Wald}[4]			<0.001			<0.001	<0.001

Men

	Never smokers			Current smokers			
	deaths	HR[2]	(95% CI)	deaths	HR[2]	(95% CI)	$p_{heterog}$[3]
**Overall**							
Never	84	1.50	(1.19, 1.21)	58	2.09	(1.26, 3.47)	
0.1-4.9	457	1	Ref	367	1.62	(1.04, 2.53)	
5-14.9	538	0.93	(0.82, 1.06)	799	1.45	(0.93, 2.25)	
15-29.9	369	1.00	(0.87, 1.16)	927	1.66	(1.06, 2.58)	
30-59.9	254	1.22	(1.23, 1.43)	857	1.83	(1.17, 2.84)	
≥ 60	107	1.56	(1.25, 1.95)	590	2.43	(1.55, 3.80)	
p_{Wald}[4]			<0.001			<0.001	0.864

	Wine use			Beer use			
	deaths	HR[5]	(95% CI)	deaths	HR[5]	(95% CI)	$p_{difference}$[6]
**Overall**							
Never	1,064	1.21	(1.12, 1.30)	975	1.07	(0.98, 1.16)	
0.1-2.9	3,266	1	Ref	2,959	1	Ref	
3-9.9	2,139	0.92	(0.87, 0.97)	2,486	1.04	(0.98, 1.10)	
10-19.9	1,040	0.96	(0.89, 1.03)	1,248	1.12	(1.04, 1.20)	
20-39.9	814	1.03	(0.95, 1.13)	877	1.41	(1.30, 1.54)	
≥ 40	641	1.22	(1.10, 1.35)	419	1.86	(1.66, 2.09)	
p_{Wald}[4]			<0.001			<0.001	<0.001

[1]Models were stratified by center. Systematic adjustment was undertaken for age at recruitment, BMI and height, former drinking, time since alcohol quitting, smoking status, duration of smoking, age at start smoking, educational attainment, and energy intake. In women adjustment was undertaken for menopausal status, ever use of replacement hormones, and number of full term pregnancies; [2]Models included interaction terms between lifetime alcohol use and a smoking indicator (0=never smokers; 1=current smokers), keeping the reference category the group of moderate alcohol users (0.1-4.9 g/day) among never smokers, whereas former smokers and subjects with unknown smoking status were excluded; [3]$p_{heterogeneity}$: difference in HRs assessed comparing the log-likelihood of models with and without interaction terms between alcohol and smoking status to a 4 and 5 degrees of freedom (dof) chi-square distribution, in women and men respectively; [4]p_{Wald}: determined using a Wald test for contrasts according to a chi-square distribution with 4 and 5 dof, in women and men respectively; [5]Models on wine and beer uses were mutually adjusted, and also included spirits/liquors use; [6]$p_{difference}$ expresses the difference of associations between wine and beer use, determined evaluating the significance of the parameter estimate γ_2 in a model that included, other than the list of confounders, the terms $\gamma_1 \cdot (X_1+X_2)/2 + \gamma_2 \cdot (X_1-X_2)/2$, with X_1=log(wine use+1) and X_2=log(beer use+1).

For peer review only

Figure 2. Sex-specific plots displaying cumulative probabilities of death due to overall mortality, for heavy (≥30 g/day in women and ≥60 g/day in men, *continuous* line) and moderate lifetime use (0.1-4.9 g/day) (*dotted* line), in smokers (*black* line) and never smokers (*grey* line), for study subjects aged 60 years.

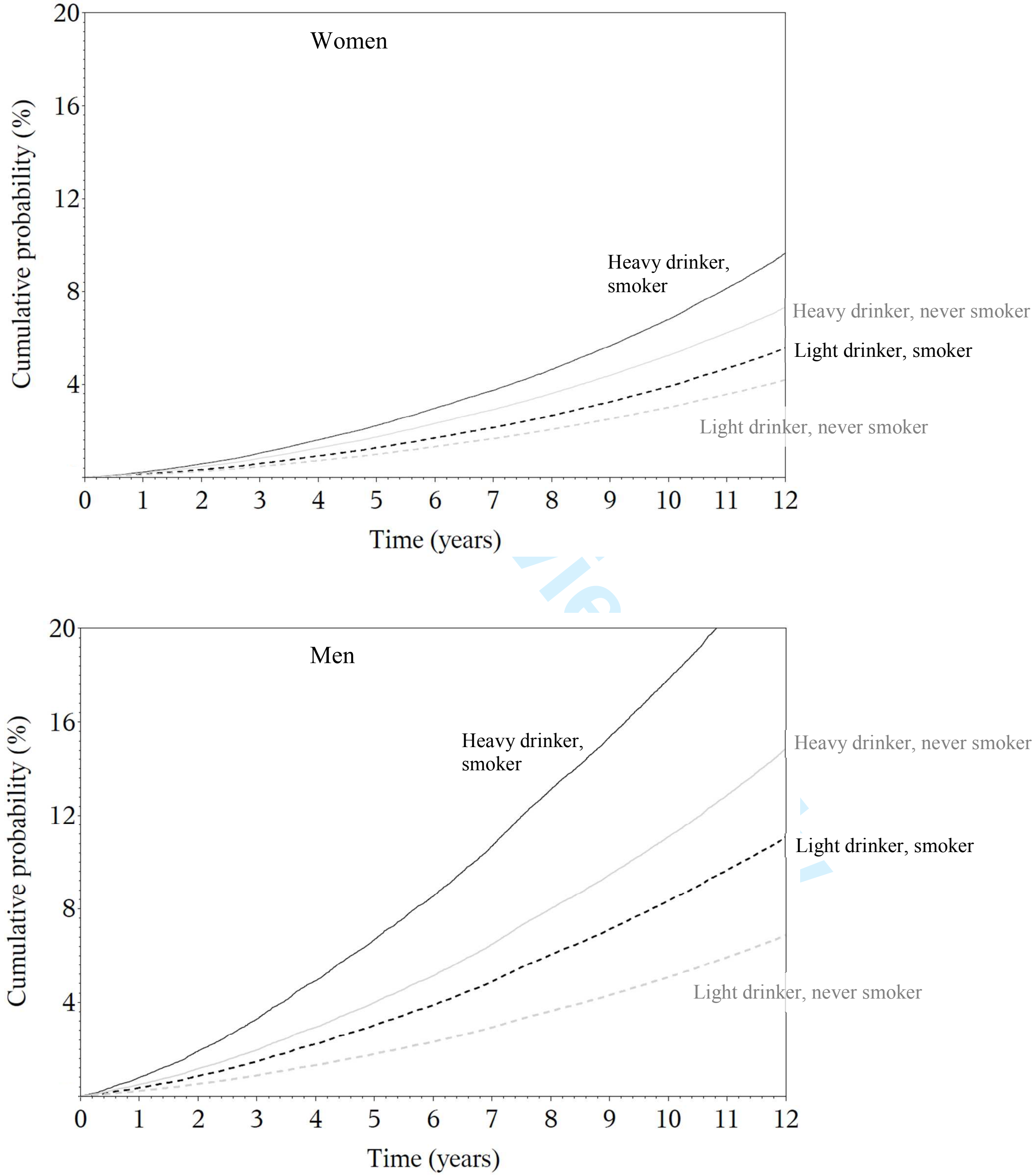

Figure 3. In competing risks analyses, sex-specific plots displaying cumulative probabilities of death due to CVD/CHD (red), alcohol related cancers (blue), and violent death and injuries (green), for study subjects aged 60 years according to heavy (≥30 g/day in women and ≥60 g/day in men, *continuous* line) and moderate (0.1-4.9 g/day, *dotted* lines) lifetime alcohol use in current and never smokers in the EPIC study.

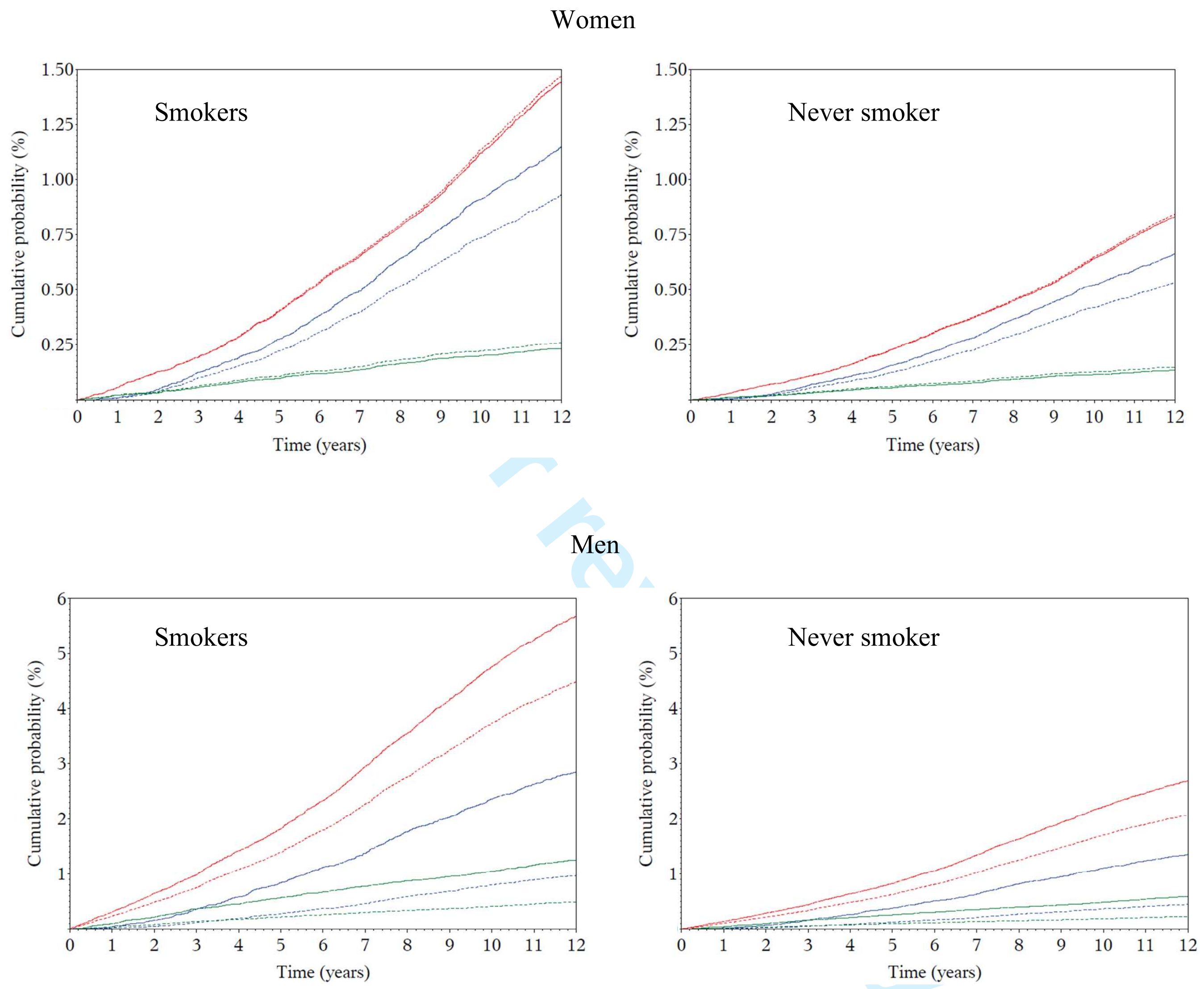

For peer review only - http://bmjopen.bmj.com/site/about/guidelines.xhtml

Table 4. Sex-specific estimates of rate advancement period (RAP) and associated 95%CI for overall and mortality due to ARCs, CVD/CHD and injuries and violent deaths, related to two scenarios of lifetime alcohol use. RAP estimates express the impact of a given exposure on the risk of death, by determining the time (in years) by which the risk of death is anticipated for study subjects exposed, e.g. all drinkers more than the threshold (5 or 15 g/day in Scenario I and II, respectively), compared to non-exposed, i.e. individuals drinking between 0.1 g/day and the threshold.

		Scenario I		Scenario II	
		Threshold: 5 g/day		Threshold: 15 g/day	
		RAP (years)	95%CI	RAP (years)	95%CI
Women	Overall	0.36	-0.05, 0.76	0.83	0.26, 1.39
	CVD/CHD	0.23	-0.46, 0.92	0.08	-0.96, 1.14
	Alcohol related cancers	1.28	-0.86, 3.41	1.90	-1.00, 4.81
	Injuries and violent deaths	-2.69	-6.85, 1.47	-0.20	-5.85, 5.46
Men	Overall	0.15	-0.48, 0.76	1.42	0.96, 1.89
	CVD/CHD	-0.53	-1.57, 0.50	-0.01	-0.82, 0.81
	Alcohol related cancers	2.59	-0.30, 5.49	5.03	3.07, 7.00
	Injuries and violent deaths	7.59	-2.82, 18.02	11.83	3.92, 18.17

[1]Never lifetime alcohol users did not enter into the estimation process.

For peer review only

MS: "Lifetime alcohol use and overall and cause-specific mortality in the European Prospective Investigation into Cancer and nutrition (EPIC) study" by Ferrari et al.

STROBE statement checklist

(Pages of the submitted manuscript where each point of the checklist is addressed are indicated)

	Item No	Recommendation
**Title and abstract**	1	(*a*) Indicate the study's design with a commonly used term in the title or the abstract **(Pages 1, 4)**
		(*b*) Provide in the abstract an informative and balanced summary of what was done and what was found **(Page 4)**
**Introduction**		
Background/rationale	2	Explain the scientific background and rationale for the investigation being reported **(Page 6)**
Objectives	3	State specific objectives, including any prespecified hypotheses **(Page 6)**
**Methods**		
Study design	4	Present key elements of study design early in the paper **(Pages 7, 8)**
Setting	5	Describe the setting, locations, and relevant dates, including periods of recruitment, exposure, follow-up, and data collection **(Pages 7, 8)**
Participants	6	(*a*) Give the eligibility criteria, and the sources and methods of selection of participants. Describe methods of follow-up **(Pages 7, 8)**
		(*b*) For matched studies, give matching criteria and number of exposed and unexposed **(NA)**
Variables	7	Clearly define all outcomes, exposures, predictors, potential confounders, and effect modifiers. Give diagnostic criteria, if applicable **(Pages 8,9)**
Data sources/ measurement	8*	For each variable of interest, give sources of data and details of methods of assessment (measurement). Describe comparability of assessment methods if there is more than one group **(Pages 8, 9)**
Bias	9	Describe any efforts to address potential sources of bias **(Pages 10,11)**
Study size	10	Explain how the study size was arrived at **(Page 8)**
Quantitative variables	11	Explain how quantitative variables were handled in the analyses. If applicable, describe which groupings were chosen and why **(Pages 8,9)**
Statistical methods	12	(*a*) Describe all statistical methods, including those used to control for confounding **(Pages 8, 9, 10, 11)**
		(*b*) Describe any methods used to examine subgroups and interactions **(Pages 10,11)**
		(*c*) Explain how missing data were addressed **(Page 9)**
		(*d*) If applicable, explain how loss to follow-up was addressed **(Page 9)**
		(*e*) Describe any sensitivity analyses **(Page 10)**
**Results**		
Participants	13*	(a) Report numbers of individuals at each stage of study—eg numbers potentially eligible, examined for eligibility, confirmed eligible, included in the study, completing follow-up, and analysed **(Pages 7, 8, 12)**
		(b) Give reasons for non-participation at each stage **(8. For the EPIC study, details are included in Reference 11, Riboli et al, PHN, 2002)**
		(c) Consider use of a flow diagram **NO FLOW DIAGRAM**
Descriptive data	14*	(a) Give characteristics of study participants (eg demographic, clinical, social) and information on exposures and potential confounders **(Pages 12, 13, Table 2)**
		(b) Indicate number of participants with missing data for each variable of interest

For peer review only - http://bmjopen.bmj.com/site/about/guidelines.xhtml

		**(Page 9)**
		(c) Summarise follow-up time (eg, average and total amount) **(Page 12, Tables 1 and 2)**
Outcome data	15*	Report numbers of outcome events or summary measures over time **(Page 12, Table 1)**
Main results	16	(*a*) Give unadjusted estimates and, if applicable, confounder-adjusted estimates and their precision (eg, 95% confidence interval). Make clear which confounders were adjusted for and why they were included **(Page 13)**
		(*b*) Report category boundaries when continuous variables were categorized **(Pages 13, 14)**
		(*c*) If relevant, consider translating estimates of relative risk into absolute risk for a meaningful time period **(Pages 14, 15)**
Other analyses	17	Report other analyses done—eg analyses of subgroups and interactions, and sensitivity analyses **(Pages 13, 14, 15)**
**Discussion**		
Key results	18	Summarise key results with reference to study objectives **(Page 15)**
Limitations	19	Discuss limitations of the study, taking into account sources of potential bias or imprecision. Discuss both direction and magnitude of any potential bias **(Page 16)**
Interpretation	20	Give a cautious overall interpretation of results considering objectives, limitations, multiplicity of analyses, results from similar studies, and other relevant evidence **(Pages 16, 17)**
Generalisability	21	Discuss the generalisability (external validity) of the study results **(Pages 17, 18)**
**Other information**		
Funding	22	Give the source of funding and the role of the funders for the present study and, if applicable, for the original study on which the present article is based **(Pages 19, 20)**

*Give information separately for exposed and unexposed groups.

Note: An Explanation and Elaboration article discusses each checklist item and gives methodological background and published examples of transparent reporting. The STROBE checklist is best used in conjunction with this article (freely available on the Web sites of PLoS Medicine at http://www.plosmedicine.org/, Annals of Internal Medicine at http://www.annals.org/, and Epidemiology at http://www.epidem.com/). Information on the STROBE Initiative is available at http://www.strobe-statement.org.

For peer review only

For peer review only - http://bmjopen.bmj.com/site/about/guidelines.xhtml

Figure 1. Number of deaths, person-years (PY) and multivariable hazard ratios[1] (HR) with 95% confidence intervals (CI) and p-value of the Wald test for statistical significance for overall and cause-specific mortality by categories of lifetime alcohol use, in women and men.

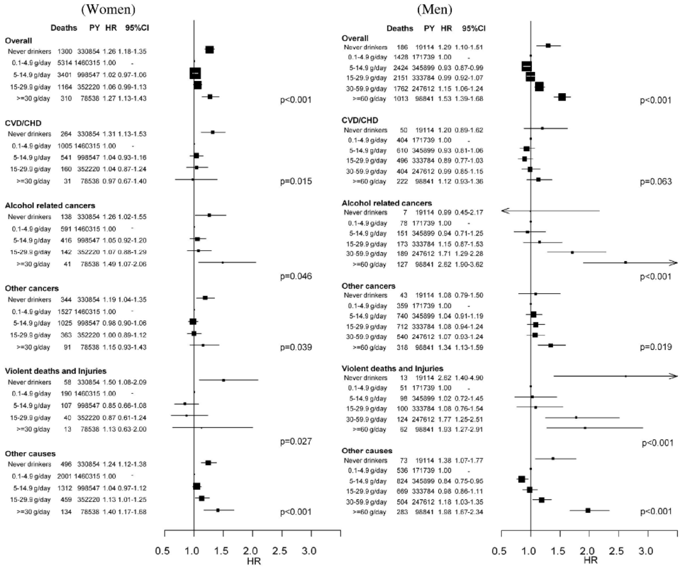

[1]Models were stratified by center. Systematic adjustment was undertaken for age at recruitment, BMI and height, former drinking, time since alcohol quitting, smoking status, duration of smoking, age at start smoking, educational attainment, and energy intake. In women adjustment was undertaken for menopausal status, ever use of replacement hormones, and number of full term pregnancies.

297x210mm (300 x 300 DPI)

review only

Figure 2. Sex-specific plots displaying cumulative probabilities of death due to overall mortality, for heavy (≥30 g/day in women and ≥60 g/day in men, continuous line) and moderate lifetime use (0.1-4.9 g/day) (dotted line), in smokers (black line) and never smokers (grey line), for study subjects aged 60 years.

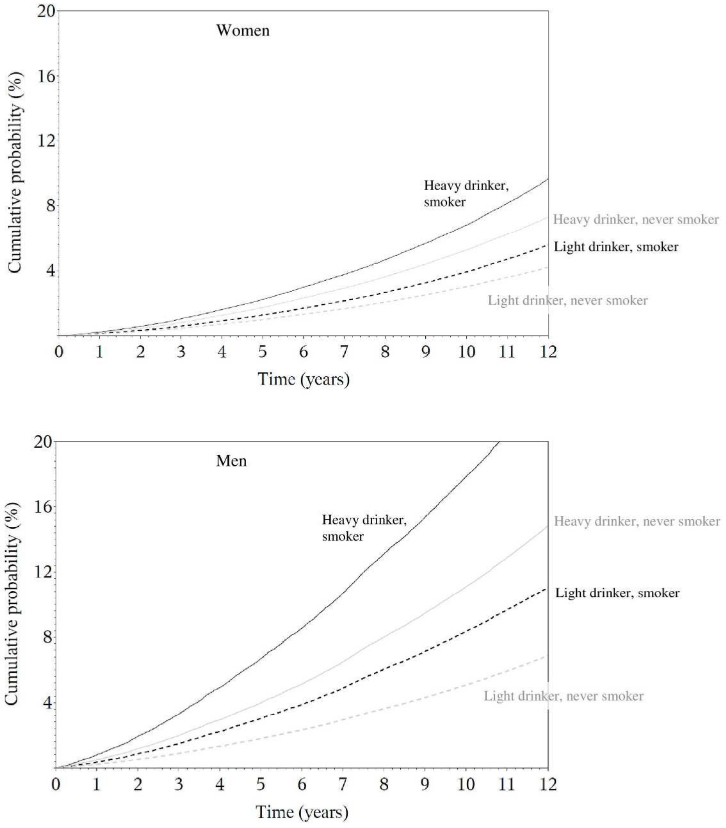

210x297mm (300 x 300 DPI)

Figure 3. In competing risks analyses, sex-specific plots displaying cumulative probabilities of death due to CVD/CHD (red), alcohol related cancers (blue), and violent death and injuries (green), for study subjects aged 60 years according to heavy (≥30 g/day in women and ≥60 g/day in men, continuous line) and moderate (0.1-4.9 g/day, dotted lines) lifetime alcohol use in current and never smokers in the EPIC study.

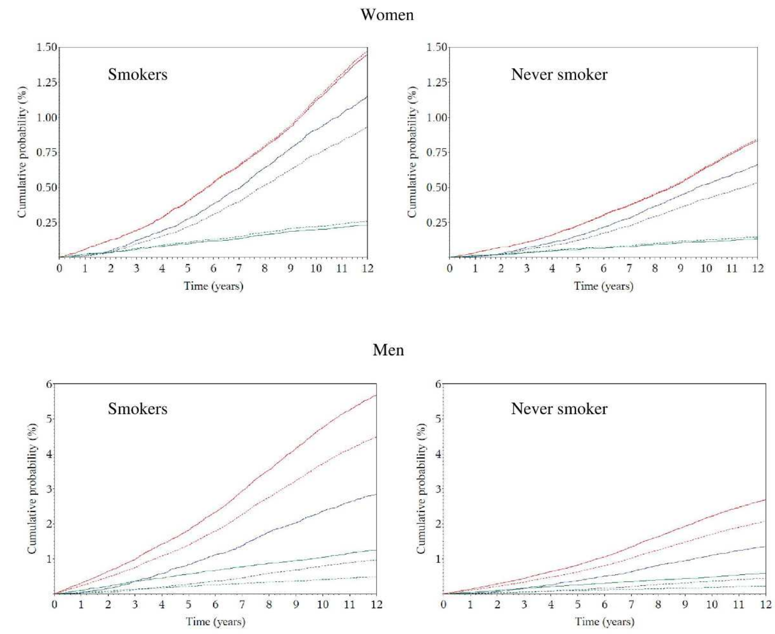

210x297mm (300 x 300 DPI)

Supplementary figure 1. Number of deaths, person-years (PY) and multivariable hazard ratios[1] (HR) with 95% confidence intervals (CI) and p-value of the Wald test for statistical significance for overall and cause-specific mortality by categories of lifetime alcohol use, in women and men.

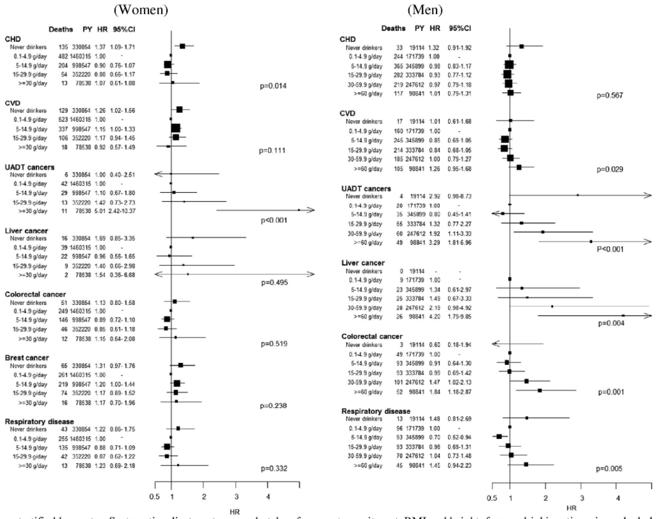

[1]Models were stratified by center. Systematic adjustment was undertaken for age at recruitment, BMI and height, former drinking, time since alcohol quitting, smoking status, duration of smoking, age at start smoking, educational attainment, and energy intake. In women adjustment was undertaken for menopausal status, ever use of replacement hormones, and number of full term pregnancies.

297x210mm (300 x 300 DPI)

For peer review only

Supplementary figure 2. Relationship between lifetime alcohol use (g/day) and overall and cause specific risk of death (and associated 95% CI) obtained by using fractional polynomials of order two. P-linearity was obtained by comparing the log-likelihood difference of models with fractional polynomials and a linear term to a chi-square distribution with 3 degrees of freedom. **Women.**

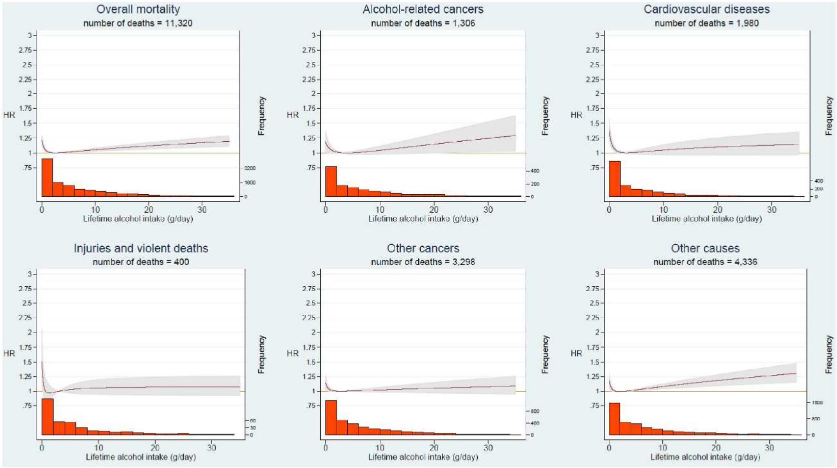

297x210mm (300 x 300 DPI)

view only

Supplementary figure 3. Relationship between lifetime alcohol use (g/day) and overall and cause specific risk of death (and associated 95% CI) obtained by using fractional polynomials of order two. P-linearity was obtained by comparing the log-likelihood difference of models with fractional polynomials and a linear term to a chi-square distribution with 3 degrees of freedom. **Men.**

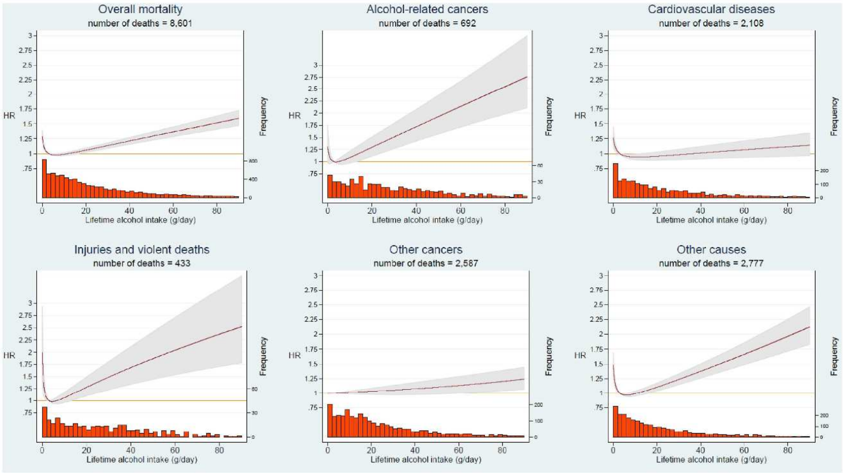

297x210mm (300 x 300 DPI)

For peer review only

Supplementary figure 4. Associations between average alcohol use (g/day) and overall mortality (and associated 95% CI) by country of origin. $P_{heterogeneity}$ was equal to 0.511, and was obtained by comparing the log-likelihood difference of models with and without interaction terms to a chi-square statistics with 32 degrees of freedom (dof). For each country, p_{Wald} was obtained comparing the Wald test statistics to a chi-square distribution with 4 dof. Country-specific geometric means for total and subtypes of alcohol use (W=wine, B=beer, Sp=spirits/liquors in g/day) are also displayed. **Women.**

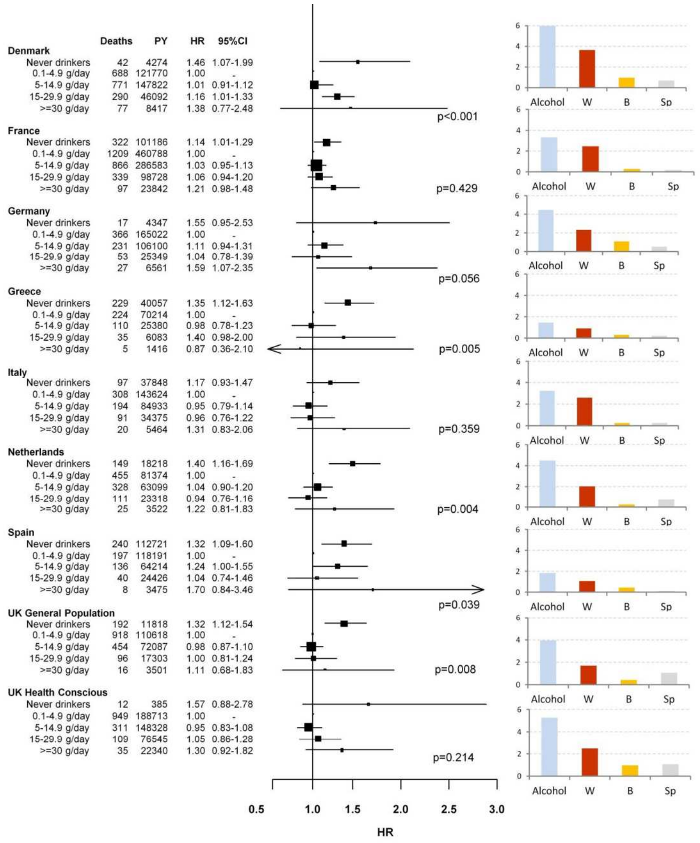

210x297mm (300 x 300 DPI)

Supplementary figure 5. Associations between average lifetime alcohol use (g/day) and overall and cause specific risk of death (and associated 95% CI) by country of origin. $P_{heterogeneity}$ was equal to 0.012, and was obtained by comparing the log-likelihood difference of models with and without interaction terms to a chi-square statistics with 25 degrees of freedom (dof). For each country, p_{Wald} was obtained comparing the Wald test statistics to a chi-square distribution with 5 dof. Country-specific geometric means for total and subtypes of alcohol use (W=wine, B=beer, Sp=spirits/liquors in g/day) are also displayed. **Men.**

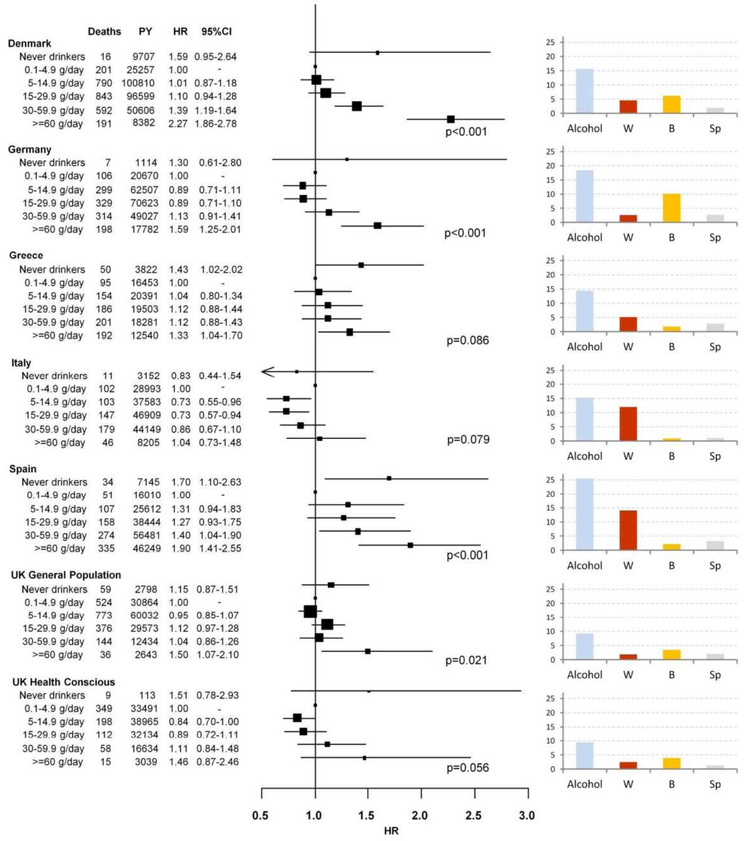

210x297mm (300 x 300 DPI)

Supplementary figure 6. HRs and survival differences for heavy versus moderate drinkers as a function of attained age (40 - 90 years). Solid lines represent predictions from the model and shaded areas depict delta-method 95% CIs for the predictions. Estimates are derived from flexible parametric survival models with age as the time-scale, with the origin of the time-scale set to 30 years. Interactions between alcohol intake and the time-scale were fitted to allow for age-varying HR.

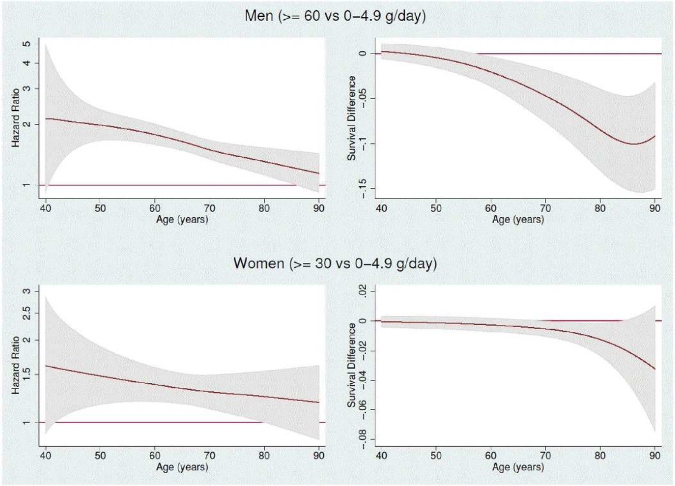

297x210mm (300 x 300 DPI)

view only
